# Mutually stabilizing interactions between proto-peptides and RNA

**DOI:** 10.1038/s41467-020-16891-5

**Published:** 2020-06-19

**Authors:** Moran Frenkel-Pinter, Jay W. Haynes, Ahmad M. Mohyeldin, Martin C, Alyssa B. Sargon, Anton S. Petrov, Ramanarayanan Krishnamurthy, Nicholas V. Hud, Loren Dean Williams, Luke J. Leman

**Affiliations:** 1 NSF/NASA Center for Chemical Evolution, Atlanta, GA USA; 2 School of Chemistry & Biochemistry, Georgia Institute of Technology, Atlanta, GA 30332 USA; 3 NASA Center for the Origins of Life, Georgia Institute of Technology, Atlanta, GA USA; 4 Department of Chemistry, The Scripps Research Institute, La Jolla, CA 92037 USA

**Keywords:** Origin of life, Coevolution, Origin of life

## Abstract

The close synergy between peptides and nucleic acids in current biology is suggestive of a functional co-evolution between the two polymers. Here we show that cationic proto-peptides (depsipeptides and polyesters), either produced as mixtures from plausibly prebiotic dry-down reactions or synthetically prepared in pure form, can engage in direct interactions with RNA resulting in mutual stabilization. Cationic proto-peptides significantly increase the thermal stability of folded RNA structures. In turn, RNA increases the lifetime of a depsipeptide by >30-fold. Proto-peptides containing the proteinaceous amino acids Lys, Arg, or His adjacent to backbone ester bonds generally promote RNA duplex thermal stability to a greater magnitude than do analogous sequences containing non-proteinaceous residues. Our findings support a model in which tightly-intertwined biological dependencies of RNA and protein reflect a long co-evolutionary history that began with rudimentary, mutually-stabilizing interactions at early stages of polypeptide and nucleic acid co-existence.

## Abstract (web summary)

Cooperative relationships are widespread among different classes of biopolymers and are predicted to have existed during emergence of life. This study shows that proto-peptides engage in mutually stabilizing interactions with RNA, providing support for the co-evolution of these molecules.

## Introduction

Cooperative relationships between different classes of biopolymers are a hallmark of biology. RNA makes protein in the ribosome and protein makes RNA in polymerases^1^. Rudimentary forerunners of such interactions must have influenced prebiotic chemical evolution. Interactions between diverse classes of molecules might have attenuated rates of degradation, promoted folding or solubility, supported ligand binding, or promoted catalysis. Cooperative interactions between molecules also could have expanded the possible mechanisms for the buildup and persistence of certain molecules in a prebiotic environment^2^. For example, in an environment of periodic, random (non-coded) oligomer synthesis via condensation processes fueled by condensing agents or wet–dry cycles, intermolecular interactions that imparted reduced rates of hydrolysis for one or more of the oligomers involved could naturally lead to a buildup of those sequences. Understanding the nature and genesis of productive molecular interactions among prebiotic molecules is a central issue in exploring the origin of life.

A prevailing idea in origins-of-life research is that there was once an RNA World in which RNA served dual roles as genetic polymer and catalyst^3–8^. Over time the pure RNA World incrementally gave rise to proteins and DNA on the path to the current RNA/DNA/protein biological system. An alternative theory is that the evolution of nucleic acids and polypeptides was intimately connected from the beginning (i.e., a Ribonucleopeptide World)^9–17^. In either scenario, it appears that productive relationships between nucleic acids and peptides (or between proto-nucleic acids and proto-peptides) would be required during maturation of the extant RNA/DNA/protein system. A few studies have investigated how peptides could promote folding or functions of RNA^18–22^, or vice versa^23,24^, but there are few examples of interactions that mutually benefit both partners^25^. Given the importance that molecular cooperation may have played in the origins of peptides and RNA, and recent descriptions of prebiotic pathways by which amino acids, peptides, and nucleotides can simultaneously be produced^26–28^, we aimed to evaluate the possibility of mutually stabilizing interactions between RNA and proto-peptides (polyesters and depsipeptides).

Polyesters and depsipeptides, which contain backbone ester linkages in place of some or all amide bonds, have been proposed as the chemical ancestors of present-day proteins (i.e., as proto-peptides). Polyesters and depsipeptides are prebiotically plausible. Not only can α-hydroxy acids be incorporated ribosomally during translation to generate depsipeptide and polyester^29,30^, but these proto-peptides also form more readily than pure peptides under mild dry-heat conditions^31–36^. Ester linkages enable the formation of amide bonds through a process of ester-amide exchange^32–34^. Hydroxy acids are produced together with amino acids in model prebiotic reactions^37^, are found together in some meteorites^37,38^, and can combine to form oligomers >20 residues in length in mild dry-down reaction conditions^31–36^. We recently demonstrated that cationic depsipeptides form robustly in dry-down reactions of mixtures of α-hydroxy acids and cationic α-amino acids^39^.

Given that electrostatics are key elements of protein–nucleic acid interactions in extant life, we hypothesized that cationic proto-peptides might functionally interact with nucleic acids. Here we show that cationic polyesters and depsipeptides, generated either as heterogeneous mixtures from dry-down reactions of α-hydroxy acid monomers alone or in combination with α-amino acids, or synthetically prepared in homogeneous pure form, can interact directly with RNA in mutually stabilizing partnerships. These interactions prolong the lifetimes of proto-peptides due to lower rates of backbone ester bond hydrolysis, and render RNA duplexes more stable against thermal denaturation. Proto-peptides containing Arg, His, or Lys adjacent to backbone ester bonds generally increase RNA duplex thermal stability to a greater extent than do analogous sequences containing non-proteinaceous ornithine (Orn), 2,4-diaminobutyric acid (Dab), or 2,3-diaminopropionic acid (Dpr). Thus, mutually stabilizing interactions appear to be a natural outcome when cationic proto-peptides and RNA co-exist in mixtures. The results of our study support the idea that the intermolecular interactions between RNA and peptides in extant biology have ancient origins and reflect a long co-evolutionary history.

## Results

### Proto-peptides produced in dry-downs increase the RNA *T*_m_

**Fig. 1:**
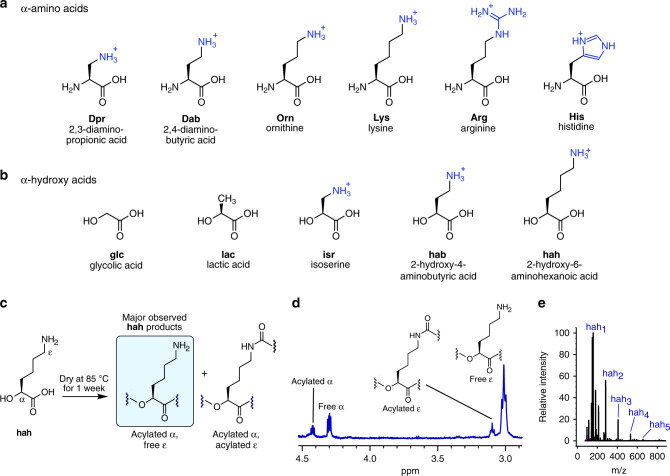
Cationic depsipeptides and polyesters are generated in dry-down reactions. Depsipeptide and polyester mixtures used in this study were generated via dry-down reactions, in the absence of condensing agents, from binary mixtures of **a** α-amino acids and **b** α-hydroxy acids. Cationic side chain moieties are blue. **c** Scheme showing some potential products of a dry-down reaction of the cationic α-hydroxy acid monomer hah. The major species observed by ^1^H-NMR were acylated at the α-hydroxy position and free at the ε-amine position, corresponding to linear oligomers having a backbone topology similar to biological proteins, but with ester bonds in place of amide bonds. **d** ^1^H-NMR spectrum of the product mixture resulting from a hah dry-down at 85 °C for 7 days. Integration of the free α-proton indicated that 56% of hah was incorporated into oligomers. The downfield ε-protons at ~3.1 ppm (12% by integration) likely correspond to ε-amidation, in analogy to chemical shift patterns observed upon ε-amidation of Lys in dry-down reactions^39^. Some resonances corresponding to acylated α-species are obscured by the water peak and are not shown, but can be observed by COSY analysis (Supplementary Fig. 8). **e** Positive-mode ESI-MS spectra showing the production of cationic polyesters via dry-down reaction of hah at 85 °C for 7 days. Labeled species correspond to [M + H]^+^ ions.

We recently reported the formation of cationic depsipeptides generated from binary mixtures of an α-hydroxy acid (glycolic acid, glc; or lactic acid, lac) (Fig. 1) and a cationic α-amino acid (lysine, Lys; arginine, Arg; histidine, His; ornithine, Orn; 2,4-diaminobutyric acid, Dab; or 2,3-diaminopropionic acid, Dpr) (Fig. 1)^39^. Based on those results, we hypothesized that dry-down reactions involving only a cationic α-hydroxy acid containing a side chain amino group should yield cationic polyesters, whereas drying them in the presence of an amino acid would yield cationic depsipeptide mixtures. We chose isoserine (isr), 2-hydroxy-4-aminobutyric acid (hab, α-hydroxy analog of Dab), or 2-hydroxy-6-aminohexanoic acid (hah, α-hydroxy analog of Lys) (Fig. 1). Indeed, oligomers were observed to form by all three cationic α-hydroxy (β/γ/ε)-amino acids examined after one week of drying at 85 °C under unbuffered, acidic conditions, as indicated by nuclear magnetic resonance (NMR) and liquid chromatography–mass spectrometry (LC–MS) analysis (Fig. 1c–e and Supplementary Figs. 1–8). Analysis by NMR indicated that isr condensed less efficiently than hab and hah, whereas hab readily cyclized into lactams (Supplementary Figs. 2 and 4–8), similar to what was previously observed for Dab^39^. While drying of these cationic α-hydroxy acid monomers could potentially form linear or branched structures (Fig. 1c), NMR analysis of the hah product mixture indicated that the majority of hah incorporated into products contained free ε-amines, indicative of a linear, protein-like backbone topology with esters in place of the peptide bonds (Fig. 1d).

**Fig. 2:**
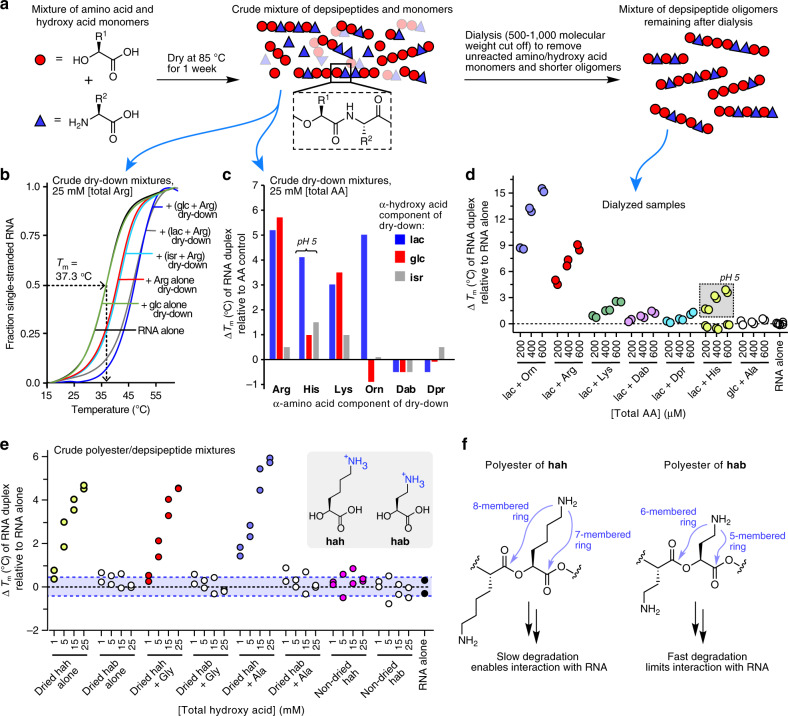
Cationic proto-peptide mixtures generated in dry-down reactions stabilize duplex RNA. Dry-down reaction products were dissolved in deionized water to give a 100 mM stock solution based on the amount of monomers used at the start of the dry-down. The mixtures contain diverse oligomers at varying abundances, so the concentration of a given oligomer in the mixture would be substantially lower. Control dry-down reactions contained the amino acid alone, with no hydroxy acid (AA control)^39^. **a** Schematic depicting the process used here to generate proto-peptide mixtures. RNA stability studies used either crude dry-down product mixtures, or dialyzed mixtures from which unreacted monomers and short oligomers had been removed with a 500–1000 Da cut-off membrane. For panels **b**–**d**, the experiments employed a 10-mer RNA duplex (5′-6-FAM-rCrGrCrUrArArArUrCrG-3′ & 5′-rCrGrArUrUrUrArGrCrG-3IABkFQ-3′, 2.5 μM strand) in buffer (100 mM MES-TEA, 2.5 mM NaCl, pH 7.5). The final pH of the samples was between 5.6 and 6.8, unless otherwise noted. **b** Thermal denaturation curves for the RNA duplex in the presence of various Arg-containing dry-down reaction mixtures or control mixtures. **c** Changes in RNA duplex *T*_m_ relative to the corresponding amino acid control (dry-down reaction of the amino acid without a hydroxy acid) for crude dry-down mixtures. **d** Changes in RNA duplex *T*_m_ upon addition of dialyzed depsipeptide oligomers. Data are shown as a scatter plot of duplicate experiments. The non-cationic glc + Ala oligomers were included as a control. For the RNA alone condition, three technical replicates from each of the duplicate experiments are shown, for a total of six data points. **e** Changes in RNA duplex *T*_m_ values upon addition of crude polyester and depsipeptide mixtures obtained by drying hab or hah, either alone or with Gly or Ala (at a 1:1 molar ratio), or non-dried controls. Data are shown as a scatter plot of two independent experiments. **f** Structures of polyesters derived from drying hab or hah, showing potential routes of oligomer degradation via intramolecular O,N acyl transfer.

We hypothesized that the mixtures of cationic proto-peptides now in hand might engage in interactions with nucleic acids. Accordingly, we determined the effect of cationic proto-peptides on RNA duplex stability by monitoring changes in the melting temperature (*T*_m_) of a short 10mer RNA duplex in the presence or absence of individual proto-peptide samples (Fig. 2). The complementary RNA strands were 5′- or 3′-labeled with either a fluorophore or a quencher so that the degree of RNA hybridization could be monitored by fluorescence in rtPCR instrumentation (Supplementary Fig. 9). As controls, we used (i) dry-down reactions containing the amino acid alone (no hydroxy acid), for which no oligomerization was observed for any amino acid (AA control); (ii) depsipeptide mixtures generated from a dry-down reaction of glc+Ala, which produced non-cationic oligomers; (iii) dry-down reactions of glc or lac alone (no amino acid), which produced non-cationic polyesters; and (iv) cationic α-hydroxy acids such as hah or hab that were not dried, and thus not oligomerized. For the *T*_m_ measurements, the proto-peptide samples were diluted to a final concentration of 25 mM (based on the amount of cationic amino acid used at the start of the dry-down) and mixed with the RNA duplex (2.5 μM each strand). After measuring the thermal denaturation curve for each condition (Fig. 2b), the observed *T*_m_ was corrected using the *T*_m_ observed for the corresponding AA control, and plotted as change in *T*_m_ (Fig. 2c).

Under these conditions, the presence of depsipeptide product mixtures that contained the amino acids Arg, His, and Lys, generally caused increases in the *T*_m_ of the RNA duplex, whereas product mixtures that contained Dab or Dpr did not affect the *T*_m_ (Fig. 2c). The Orn product mixtures had variable effects depending on the hydroxy acid that was present in the dry-down; Orn + lac depsipeptides increased the RNA duplex *T*_m_, whereas the Orn + glc depsipeptide mixture caused a slight reduction in the *T*_m_ compared with the AA control. In general, the depsipeptide mixtures containing isr affected the *T*_m_ less than the analogous lac or glc mixtures (Fig. 2c); this may be due to the possibility in these sequences for intramolecular O,N acyl transfer rearrangements that would reduce the cationic charge of the oligomer. We prepared two additional replicate series of dry-down reactions for the lac mixtures to confirm the reproducibility of the observed effects on *T*_m_ (Supplementary Fig. 10). The observation that the presence of proteinaceous amino acids (Arg, His, and Lys) in the proto-peptides increased the *T*_m_ of the RNA duplex more effectively than the non-proteinaceous analogs Dab and Dpr can be explained by our previous findings that the Arg, His, and Lys oligomers were longer and more cationic than the Dab and Dpr dry-down products^39^, and thereby offer more stabilizing non-covalent points of contact with the RNA duplex. Moreover, depsipeptides containing Arg, His, and Lys are more stable than those containing Orn or Dab (vide infra).

We speculated that longer depsipeptides would be more effective than shorter ones in increasing the *T*_m_ of the RNA duplex due to their higher charge. To confirm that the observed effects on RNA *T*_m_ stemmed from the oligomeric products, we dialyzed the lac- or glc-containing mixtures (Fig. 2a). Following the dialysis, the resulting mixtures were enriched in longer oligomers and largely free of monomeric amino acids and hydroxy acids (Supplementary Fig. 11). In general, similar trends on RNA duplex *T*_m_ were observed for the dialyzed samples as for the crude dry-down mixtures (Fig. 2d, Supplementary Fig. 12). The magnitude of effects on *T*_m_ with the dialyzed samples were greater than those for the crude samples even though the total amino acid concentrations of 200–600 μM was lower, consistent with the assumption that longer oligomers would have greater impact on *T*_m_. Because neutral depsipeptides are not expected to interact strongly with RNAs, dialyzed dry-down mixtures of glc with alanine (Ala) served as a negative control for the dialysis experiments. Indeed, the non-cationic glc + Ala depsipeptides did not affect the *T*_m_ of the RNA duplex (Fig. 2d). Oligomers containing His increased the RNA duplex *T*_m_ at pH 5, but not at pH 7 (Fig. 2d), consistent with the expectation that His-containing oligomers would be cationic only at pH values below the His pKa of ~6.0.

We found that cationic polyesters and depsipeptides containing hah, but not hab, increased RNA duplex stability in a concentration-dependent fashion (Fig. 2e, Supplementary Fig. 13). The striking difference in effects on *T*_m_ for hah vs. hab is consistent with the more facile potential routes of oligomer degradation available to hab via intramolecular O,N acyl transfer. Whereas hab could potentially degrade via intramolecular 5- or 6-membered ring transition states during the course of the RNA *T*_m_ measurements at neutral pH, the analogous intramolecular reactions in hah would require less favorable 7- or 8-membered ring transition states (Fig. 2f). As negative controls, we used samples of hah and hab that had not been subjected to the dry-down reaction (non-dried), and indeed these samples had no effect on the *T*_m_ of the RNA duplex (Fig. 2e).

### Depsipeptide/RNA structure–function relationship

**Fig. 3:**
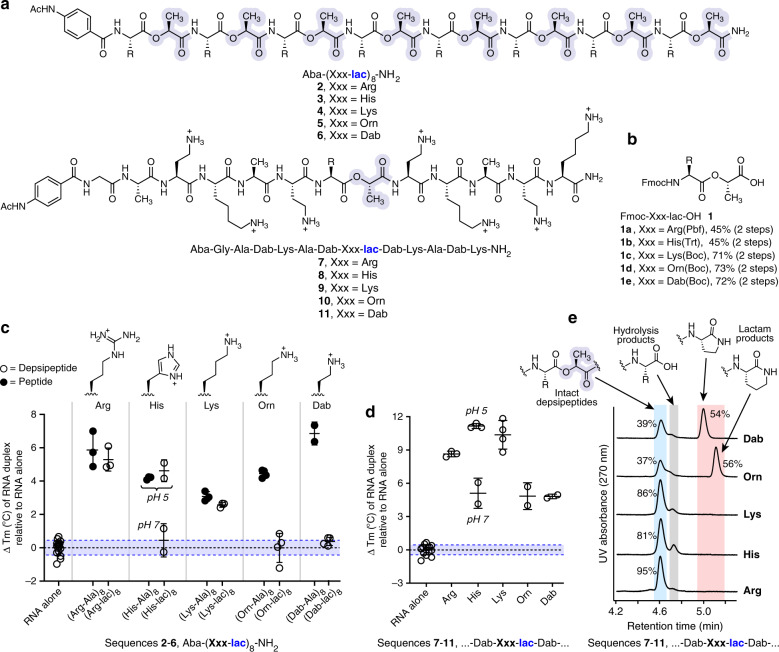
Structure–function studies of cationic depsipeptides in stabilizing an RNA duplex. Each sample contained RNA **duplex 1** (5′-rCrGrCrUrArArArUrCrG-3′ and 5′-rCrGrArUrUrUrArGrCrG-3′, 2.5 μM strand) and peptide/depsipeptide (100 μM) in buffered solution (10 mM phosphate, 100 mM NaCl, pH 7.0 or 10 mM acetate, 100 mM NaCl, pH 5.0). **a** Structures of depsipeptides used to systematically characterize cationic side chain effects on RNA duplex stability. Lactic acid residues are highlighted. Ac acetyl, Aba acetamidobenzoic acid, which was appended to the N-terminus to increase UV absorbance. **b** Structures of the Fmoc-didepsipeptide building blocks used for solid-phase synthesis of depsipeptide sequences. Fmoc fluorenylmethoxycarbonyl. **c** Comparative effects of oligo-didepsipeptide sequences **2–6** and analogous oligo-dipeptide and on the *T*_m_ of the RNA duplex (number of independent measurements: *n* = 3 for Arg and Lys sequences; *n* = 2 for His and Dab sequences; *n* = 4 for Orn sequences). The shaded areas correspond to the mean ± SD *T*_m_ measured for the RNA duplex alone (*n* = 16 independent measurements). Data are shown as a scatter plot with mean ± SD. **d** Comparative effects on RNA duplex *T*_m_ of depsipeptide sequences containing a single backbone ester (number of independent measurements: *n* = 3 for Arg; *n* = 2 for His, Orn, and Dab; *n* = 4 for Lys), relative to RNA duplex alone (*n* = 16 independent measurements). Data are shown as a scatter plot with mean ± SD. **e** To assess the impact of different cationic side chains on depsipeptide degradation rates, depsipeptides **7–11** (40 μM) were incubated at 37 °C in buffer (100 mM HEPES, 10 mM NaCl, pH 7.3). After 5 min, the reactions were quenched by the addition of 3% TFA, and samples were analyzed by HPLC. Consistent with the hypothesis that sequences containing Orn or Dab adjacent to a backbone ester bond would undergo facile intramolecular O,N acyl transfer, only 37–39% of the starting depsipeptide remained, predominantly due to the formation of lactam products. In contrast, intramolecular degradation products were not observed for the sequences containing Arg, His, or Lys adjacent to the ester bond, and >80% of the intact depsipeptide were observed in these cases.

To facilitate a more controlled structure–function relationship study for depsipeptide interactions with RNA than is possible with the complex oligomer mixtures described above, we synthesized a library of cationic depsipeptides and peptides using solid-phase protocols. The sequences varied in the cationic side chains and in the number and location of ester linkages within the depsipeptide backbone (Fig. 3, Table 1, Supplementary Table 1). To incorporate the ester bonds during solid phase synthesis, Fmoc-protected didepsipeptide building blocks **1a**–**1e** were synthesized for each of the required amino acids (Fig. 3b)^40^.

**Table 1:** Effects of synthetic peptides and depsipeptides on RNA duplex thermal stability.

Entry	Sequence	# Cationic residues	RNA duplex 1 Δ*T*_m_ (°C)	RNA duplex 2 Δ*T*_m_ (°C)	RNA duplex 3 Δ*T*_m_ (°C)	H26a RNA Δ*T*_m_ (°C)
1	Ac-Lys-NH_2_ (1 mM)	+1	0.0			
2	Ac-Tyr-Gly-Ala-Dab-Lys-NH_2_ (400 μM)	+2	−0.1	−0.2	−1.1	1.3
3	Ac-Tyr-Gly-Ala-Dab-Lys-Ala-Dab-Lys-NH_2_ (200 μM)	+4	0.7			
4	Ac-Tyr-Gly-Ala-Dab-Lys-Ala-Dab-Lys-Ala-Dab-Lys-NH_2_ (133 μM)	+6	3.4			
5	Ac-Tyr-Gly-Ala-Dab-Lys-Ala-Dab-Lys-Ala-Dab-Lys-Ala-Dab-Lys-NH_2_ (100 μM)	+8	8.1			9.5
6	Ac-Tyr-Gly-Ala-Dab-Lys-**lac**-Dab-Lys-**lac**-Dab-Lys-**lac**-Dab-Lys-NH_2_ (**14**)	+8	8.6	9.1	10.0	10.4
7	Ac-Tyr-Gly-Ala-Dab-Lys-Ala-Dab-Lys-Ala-Dab-Lys-Ala-Dab-Lys-Ala-Dab-Lys NH_2_ (80 μM)	+10	9.6			
8	Aba-Gly-Ala-Dab-Lys-Ala-Dab-Arg-**lac**-Dab-Lys-Ala-Dab-Lys-NH_2_ (**7**)	+8	8.7	8.1	12.0	11.4
9	Aba-Gly-Ala-Dab-Lys-Ala-Dab-His-**lac**-Dab-Lys-Ala-Dab-Lys-NH_2_ (**8**) (pH 5.0)	+8	11.1			
10	Aba-Gly-Ala-Dab-Lys-Ala-Dab-His-**lac**-Dab-Lys-Ala-Dab-Lys-NH_2_ (**8**) (pH 7.0)	+8	5.1			
11	Aba-Gly-Ala-Dab-Lys-Ala-Dab-Lys-**lac**-Dab-Lys-Ala-Dab-Lys-NH_2_ (**9**)	+8	10.4	8.2	9.9	11.5
12	Aba-Gly-Ala-Dab-Lys-Ala-Dab-Orn-**lac**-Dab-Lys-Ala-Dab-Lys-NH_2_ (**10**)	+8	4.9	8.2	2.5	3.5
13	Aba-Gly-Ala-Dab-Lys-Ala-Dab-Dab-**lac**-Dab-Lys-Ala-Dab-Lys-NH_2_ (**11**)	+8	4.9			
14	Aba-Gly-Ala-Asp-Glu-Ala-Asp-Lys-**lac**-Asp-Glu-Ala-Asp-Glu-NH_2_	−6	0.3	0.7	−1.5	1.1
15	Aba-Arg-Ala-Arg-Ala-Arg-Ala-Arg-Ala-Arg-Ala-Arg-Ala-Arg-Ala-Arg-Ala-NH_2_	+8	5.9			
16	Aba-Arg-**lac**-Arg-**lac**-Arg-**lac**-Arg-**lac**-Arg-**lac**-Arg-**lac**-Arg-**lac**-Arg-**lac**-NH_2_ (**2**)	+8	5.3			
17	Aba-His-Ala-His-Ala-His-Ala-His-Ala-His-Ala-His-Ala-His-Ala-His-Ala-NH_2_ (pH 5.0)	+8	4.2			
18	Aba-His-**lac**-His-**lac**-His-**lac**-His-**lac**-His-**lac**-His-**lac**-His-**lac**-His-**lac**-NH_2_ (**3**) (pH 5.0)	+8	4.6			
19	Aba-His-**lac**-His-**lac**-His-**lac**-His-**lac**-His-**lac**-His-**lac**-His-**lac**-His-**lac**-NH_2_ (**3**) (pH 7.0)	+8	0.5			
20	Ac-Tyr-Gly-Lys-Ala-Lys-Ala-Lys-Ala-Lys-Ala-Lys-Ala-Lys-Ala-Lys-Ala-Lys-Ala-NH_2_	+8	3.1	3.0	4.5	4.1
21	Aba-Lys-**lac**-Lys-**lac**-Lys-**lac**-Lys-**lac**-Lys-**lac**-Lys-**lac**-Lys-**lac**-Lys-**lac**-NH_2_ (**4**)	+8	2.6	1.5	3.9	6.3
22	Ac-Tyr-Gly-Orn-Ala-Orn-Ala-Orn-Ala-Orn-Ala-Orn-Ala-Orn-Ala-Orn-Ala-Orn-Ala-NH_2_	+8	4.5	7.6	8.3	9.0
23	Aba-Orn-**lac**-Orn-**lac**-Orn-**lac**-Orn-**lac**-Orn-**lac**-Orn-**lac**-Orn-**lac**-Orn-**lac**-NH_2_ (**5**)	+8	0.0	1.0	0.5	−1.1
24	Ac-Tyr-Gly-Dab-Ala-Dab-Ala-Dab-Ala-Dab-Ala-Dab-Ala-Dab-Ala-Dab-Ala-Dab-Ala-NH_2_	+8	6.9	8.0	11.0	10.3
25	Aba-Dab-**lac**-Dab-**lac**-Dab-**lac**-Dab-**lac**-Dab-**lac**-Dab-**lac**-Dab-**lac**-Dab-**lac**-NH_2_ (**6**)	+8	0.4	2.6	−0.4	0.5
26	Ac-Tyr-Gly-Dpr-Ala-Dpr-Ala-Dpr-Ala-Dpr-Ala-Dpr-Ala-Dpr-Ala-Dpr-Ala-Dpr-Ala-NH_2_	+8	0.9			
27	5-FAM-Gly-Ala-Dab-Lys-Ala-Dab-Lys-Ala-Dab-Lys-Ala-Dab-Lys-Ala-Dab-Lys NH_2_ (**12**) (80 μM)	+10	10.6			
28	5-FAM-Gly-Ala-Dab-Lys-Ala-Dab-Lys-Ala-Dab-Lys-**lac**-Dab-Lys-Ala-Dab-Lys NH_2_ (**13**) (80 μM)	+10	12.2			
29	Ac-Tyr-Gly-Ala-Lys-Lys-Ala-Ala-Lys-Lys-Ala-Ala-Lys-Lys-Ala-Ala-Lys-Lys-Ala-NH_2_	+8	3.4			
30	Ac-Tyr-Gly-Ala-Dab-Dab-Ala-Ala-Dab-Dab-Ala-Ala-Dab-Dab-Ala-Ala-Dab-Dab-Ala-NH_2_	+8	6.8	8.9	9.9	8.4
31	Ac-Tyr-Gly-Ala-Dab-Dab-Ala-**lac**-Dab-Dab-Ala-Ala-Dab-Dab-Ala-**lac**-Dab-Dab-Ala-NH_2_	+8	7.1	7.2	10.6	10.9
32	Ac-Tyr-Gly-Gly-Lys-Lys-Gly-Lys-Lys-Gly-Lys-Lys-Gly-Lys-Lys-NH_2_	+8	5.8			
33	Ac-Tyr-Gly-Gly-Lys-Lys-Gly-Lys-Lys-Gly-Lys-Lys-Gly-Lys-Lys-NH_2_	+8	5.8			
34	Ac-Tyr-Gly-Gly-Lys-Lys-Gly-Lys-Lys-Gly-Lys-Lys-Gly-Lys-Lys-NH_2_	+8	6.3			

RNA **duplex 1** = 5′-rCrGrCrUrArArArUrCrG-3’ & 5’-rCrGrArUrUrUrArGrCrG-3′; RNA **duplex 2** = 5′-rArArArArUrUrUrArUrArUrUrArUrUrA-3′ and 5′-rUrArArUrArArUrArUrArArArUrUrUrU-3′; RNA **duplex 3** = 5′-rArArCrGrUrArUrArCrGrUrU-3′ (palindromic); **H26a RNA** = 5′-rArUrGrArGrUrArArCrCrGrUrArArGrGrUrGrArArArUrU-3′. RNA was present in each assay at a final concentration of 2.5 μM of the folded structure (2.5 μM each strand for **duplex 1** and **duplex 2**, 5.0 μM palindromic strand for **duplex 3**, 2.5 μM strand for **H26a RNA**). The observed *T*_m_ values of the RNA structures alone at pH 7.0 were 43.8 ± 0.4 °C for **duplex 1**, 33.7 ± 0.7 °C for **duplex 2**, 48.6 ± 0.7 °C for **duplex 3**, and 53.4 ± 0.5 °C for **H26a RNA**. The observed *T*_m_ values of RNA **duplex 1** alone at pH 5.0 was 41.5 ± 0.3 °C. Unless otherwise noted, the concentration of peptide/depsipeptide was 100 μM and the pH of the measurement was 7.0. Data represent the mean ± SD of 2–4 replicate experiments. The buffers contained 10 mM phosphate, 100 mM NaCl at pH 7.0; or 10 mM acetate, 100 mM NaCl at pH 5.0. Aba = acetamidobenzoic acid, which was appended to the N-terminus of some sequences for improved UV absorbance. Underlined residues denote d-chirality. Lactic acid residues are shown in bold. Δ*T*_m_ values are the difference in *T*_m_ for a given entry compared to the RNA alone at the matched pH value.

We prepared two series of depsipeptides, **2**–**6** and **7**–**11** (Fig. 3a), to systematically study the effect of the cationic side chain on RNA duplex stabilization^41,42^. Sequences **2**–**6** each contained 8 ester bonds in the context of an oligo-didepsipeptide repeat, whereas **7**–**11** contained a single ester bond directly adjacent to a variable cationic amino acid. For depsipeptides **2**–**6**, we observed that the proteinaceous amino acids His (at pH 5, where the imidazole side chain tends to be protonated), Arg, and Lys significantly increased the duplex *T*_m_, whereas Orn and Dab did not (Fig. 3c, Table 1, Supplementary Table 1). In contrast, Orn and Dab, in the context of the analogous all-peptide backbone, substantially increased the RNA duplex *T*_m_ (Fig. 3c, Table 1, Supplementary Table 1), indicating that Orn and Dab are not inherently deficient in stabilizing RNA duplexes. Similar results were observed with depsipeptides **7**–**11**, where Arg, His, and Lys promoted greater increases in the duplex *T*_m_ than Orn or Dab (Fig. 3d). The ineffectiveness of sequences containing Orn and Dab adjacent to ester bonds can be explained by more facile intramolecular O,N-acyl transfer reactions in these structures compared to Arg, Lys, or His (Fig. 3e), leading to the degradation of Orn- and Dab-containing sequences during the course of the thermal denaturation experiment (Supplementary Fig. 14). In contrast to Orn and Dab, the presence/number of ester bonds did not impact the observed effect on RNA duplex *T*_m_ in depsipeptides containing Arg, His, or Lys adjacent to an ester bond (for examples, compare Table 1 entries #5 vs. #6, #15 vs. #16, #27 vs. #28, or #30 vs. #31).

Several lines of evidence support the importance of electrostatic interactions in the observed stabilizations of RNA duplexes. First, as would be expected, higher numbers of cationic residues present in the depsipeptide/peptide led to greater increases in RNA duplex *T*_m_ (Table 1, entries #1–7). Second, the effect of His-containing depsipeptides was pH-dependent (Figs. 2d and 3c, d), as expected based on the His pKa of ~6.0. Along the same lines, a Dpr-containing sequence did not increase the *T*_m_ of the RNA duplex (Table 1, entry #28). The ineffectiveness of this sequence is probably due to the low pKa of ~6.3 for the side chain β-amine of Dpr when incorporated into oligomeric sequences^43^. In addition, as a negative control, we verified that a negatively charged depsipeptide containing Asp and Glu residues did not affect the thermal stability of the RNA duplex (Table 1, entry #14). Furthermore, peptides composed of all d-amino acids or mixed d- and l-stereochemistry exerted the same effects on *T*_m_ values as an all l-peptide (Table 1, entries #32-34), implying that long-range electrostatics play the more dominant role than peptide stereochemistry/conformation.

To establish the generality of thermal stabilization of folded RNA structures by cationic depsipeptides, we measured the effect of a number of cationic peptides and depsipeptides on three additional RNA sequences (Table 1, Supplementary Table 1). Whereas RNA **duplex 1** is composed of two complementary 10mer strands containing 50% GC-content, RNA **duplex 2** consists of two 16mer strands with 0% GC-content, RNA **duplex 3** consists of a 12mer palindromic strand with 33% GC-content, and H26a RNA is a 23mer hairpin with 35% GC-content. H26a RNA, which is derived from ribosomal RNA, adopts a more complex, hairpin-like fold compared to the duplexes. All RNAs showed similar *T*_m_ increases by a given peptide/depsipeptide sequence (Table 1, Supplementary Table 1), suggesting that RNA stabilization by cationic proto-peptides is a general characteristic of folded RNA, which would be modulated by proto-peptide sequence, side chains, and backbone composition.

### Binding studies for cationic depsipeptides and RNA

**Fig. 4:**
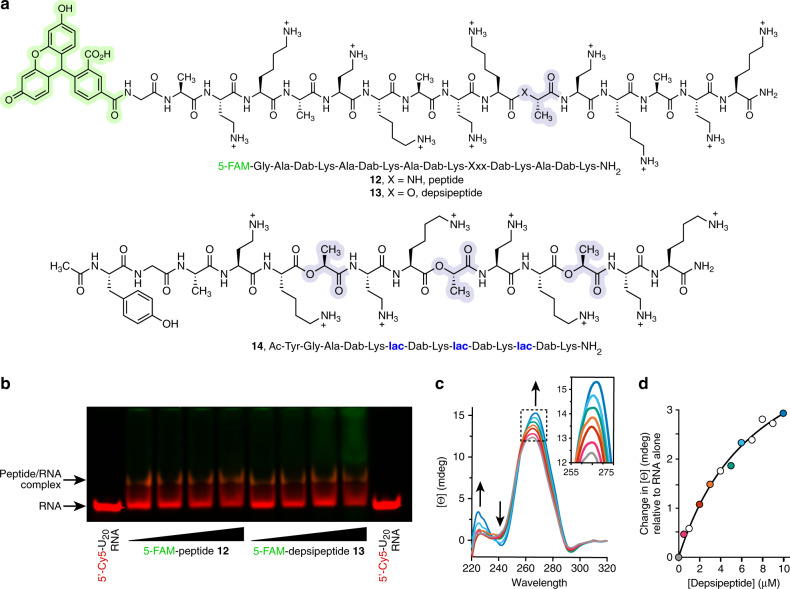
Cationic depsipeptides directly interact with RNA. **a** Structures of cationic peptide **12** and depsipeptide **13** used for gel mobility shift assays, and depsipeptide **14** used for circular dichroism studies. **b** Gel mobility shift assay with increasing concentrations (66.6–530 µM) of the FAM-labeled sequences **12** or **13** (green fluorescence) and a 5′-Cy5-U_20_ RNA (26.6 µM, red fluorescence) in MES-TEA buffer (pH 6). A physical association between the RNA and cationic oligomers is evident as a less mobile band in the gel, which appears as orange due to co-localization of the green and red dyes, and as loss of intensity of the free RNA band. The gel was cropped at the edges for clarity. The image shown is representative of two independent experiments giving similar results. **c** CD spectra of RNA **duplex 1** (5 μM each strand) with increasing concentrations of depsipeptide **14** (0–10 μM), indicating a concentration-dependent association. Spectra were recorded in 100 mM MES-TEA buffer (pH 6). **d** Plot of the change in CD signal at 266 nm as a function of increasing concentrations of depsipeptide **14**. Red and blue colors of the filled circles correspond to curves of the same color in panel (**c**). Data were fit to a simple one-site binding model (black line) yielding an apparent *K*_d_ of ~3 μM.

To establish that depsipeptides were directly associating with the RNA duplex, we used a gel mobility shift assay and circular dichroism (CD) spectroscopy. Using 5-FAM-labeled peptide **12** (Table 1, entry #27) or analogous depsipeptide **13** (Table 1, entry #28) (Fig. 4a), we observed a concentration-dependent band shift for the single-stranded RNA 5′-Cy5-U_20_ (Fig. 4b). The observed affinity for RNA inferred from the gel shift assay was similar for the depsipeptide vs. the peptide. Band shifts were also detected for other cationic peptides and depsipeptides (Supplementary Fig. 15). In agreement with the gel mobility shift assay, changes were observed in the CD spectra of the 10mer RNA duplex upon addition of the depsipeptide **14** or its corresponding peptide analog Ac-Tyr-Gly-(Ala-Dab-Lys)_4_-NH_2_, further supporting direct association between the cationic oligomers and RNA (Fig. 4c, d, Supplementary Fig. 16). Since the CD spectra were recorded at 5 °C, below the *T*_m_ of the RNA duplex (29.5 ± 0.6 °C), we attribute the changes in the spectra to alterations in RNA duplex conformation upon binding of the cationic depsipeptide/peptides. Fitting of the CD data using a one-site binding model yielded a *K*_d_ value of ~3 μM (Fig. 4d).

### RNA prolongs the lifetime of cationic depsipeptides

**Fig. 5:**
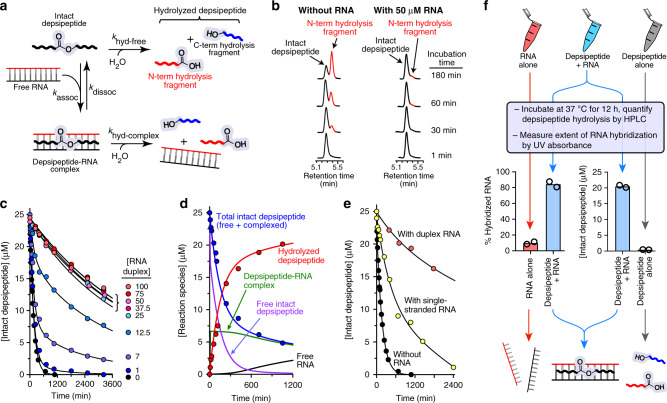
RNA increases the hydrolytic lifetime of a cationic depsipeptide. **a** A schematic of depsipeptide-RNA interactions leading to increased depsipeptide lifetimes. The free depsipeptide is hydrolyzed with a pseudo-first rate constant, *k*_hyd-free_. Binding of the depsipeptide to RNA is governed at equilibrium by *k*_assoc_/*k*_dissoc_. The pseudo-first rate constant for of depsipeptide hydrolysis within the depsipeptide–RNA complex is *k*_hyd-complex_. Under conditions where complex formation is favorable and *k*_hyd-complex_ < *k*_hyd-free_, the presence of RNA will increase the depsipeptide lifetime. **b** HPLC traces (270 nm) showing hydrolysis of depsipeptide **9** (25 μM) at various time points in the presence or absence of RNA **duplex 1** at 37 °C in pH 7.3 buffer. The C-terminal fragment of the hydrolyzed depsipeptide is not observed because it lacks the Aba chromophore. (**c**) Time courses for hydrolysis of depsipeptide **9** (25 μM) with varying concentrations of the RNA **duplex 1**. The curves shown are from simultaneous fits of data to the model given in panel **a** using SimFit^44^. During fitting, we fixed *k*_assoc_ = 1 × 10^5^ M^−1^ s^−1^. Therefore, three rate constants were fit, with values obtained by the fitting of: *k*_hyd-free_ = 1.1 × 10^−4^ s^−1^, *k*_hyd-complex_ = 3.2 × 10^−6^ s^−1^, and *k*_dissoc_ = 8.3 × 10^−3^ s^−1^. **d** Kinetic profile of the hydrolysis reaction of depsipeptide **9** (25 μM) in the presence of 7 μM RNA **duplex 1**. Observed data are shown as filled circles, while the curves represent concentrations of molecular species predicted by SimFit modeling. **e** Comparison of depsipeptide hydrolysis in the absence of RNA, or in the presence of single-stranded RNA (100 μM 5′-rCrGrArUrUrUrArGrCrG-3′) or RNA **duplex 1** (50 μM each strand). **f** To illustrate the mutually increased depsipeptide lifetime and RNA duplex *T*_m_, three samples were prepared in parallel: one containing only RNA **duplex 1** (25 μM each complementary strand), one containing only depsipeptide **9** (25 μM), and one containing both the RNA and **9** (at a 1:1 molar ratio). The extent of depsipeptide hydrolysis and RNA hybridization were then measured. Data are shown as a scatter plot of two independently repeated experiments.

We suspected that the formation of RNA-depsipeptide complexes could slow the hydrolysis rate of backbone ester bonds by a number of possible mechanisms. For instance, formation of a complex could sterically restrict access of water to the ester bond, might alter the bond geometries and conformational flexibility of the depsipeptide, or could reduce the extent of general base catalysis of ester hydrolysis by engaging the cationic side chains in electrostatic interactions with the RNA. To test the hypothesis that proto-peptide backbone hydrolysis would be slowed in the presence of RNA, we incubated depsipeptide **9** in the absence or presence of varying concentrations of RNA **duplex 1** or single-stranded RNA at 37 °C in pH 7.3 buffer (Fig. 5). High-performance liquid chromatography (HPLC) was used to monitor the concentrations of intact depsipeptide and its degradation products over time (Fig. 5b). Indeed, the presence of the RNA duplex increased the observed lifetime of the depsipeptide by up to ~30-fold (Fig. 5c, d). At all tested RNA duplex concentrations equal to or greater than 25 μM (equivalent to the initial concentration of depsipeptide **9**), the observed kinetics of depsipeptide hydrolysis were essentially identical (Fig. 5c). When only a single strand of RNA was present (no duplex), the single-stranded 10mer RNA (5′-rCrGrArUrUrUrArGrCrG-3′, 100 µM) increased the depsipeptide lifetime, but to a lesser extent (lifetime increased by roughly fivefold) compared to duplex RNA (Fig. 5e).

Data from the nine independent depsipeptide hydrolysis reactions carried out with different concentrations of RNA **duplex 1** (Fig. 5c) were simultaneously fit using SimFit^44^ to the reaction model shown in Fig. 5a. In the model, the free depsipeptide is subject to hydrolysis at a pseudo-first order rate governed by *k*_hyd-free_. Binding of the depsipeptide to RNA according to an equilibrium determined by *k*_assoc_/*k*_dissoc_ yields a depsipeptide–RNA complex, for which the pseudo-first order rate of ester hydrolysis is described by *k*_hyd-complex_. Under conditions where depsipeptide–RNA complex formation is favorable and *k*_hyd-RNA_ < *k*_hyd-free_, the presence of RNA will reduce the observed rate of depsipeptide hydrolysis. During fitting, we fixed *k*_assoc_ = 1 × 10^5^ M^−1^ s^−1^, on the lower end of rates observed and expected for protein-RNA association^45^. Therefore, three parameters were variable during the fitting, and the final rate constants determined by the fitting were: *k*_hyd-free_ = 1.1 × 10^−4^ s^−1^, *k*_hyd-complex_ = 3.2 × 10^−6^ s^−1^, and *k*_dissoc_ = 8.3 × 10^−3^ s^−1^. Based on these values, the rate constant for backbone ester hydrolysis in the depsipeptide–RNA complex (*k*_hyd-complex_) was ~34-fold lower than for the free depsipeptide (*k*_hyd-free_). Given the fixed value of *k*_assoc_ and the calculated value of *k*_dissoc_, the predicted dissociation constant of the depsipeptide **9**-RNA complex is 0.1 μM, somewhat lower than the *K*_d_ of ~3 μM measured by CD for depsipeptide sequence **14** binding to the same RNA duplex.

To demonstrate the simultaneous mutual stabilization that RNA and cationic depsipeptides gain through their non-covalent interactions, three parallel samples were prepared: one sample containing only RNA **duplex 1** (25 μM each complementary strand), one containing only depsipeptide **9** (25 μM), and the last sample containing both **duplex 1** and depsipeptide **9** (Fig. 5f). After incubation at 37 °C for 12 h, the extent of depsipeptide hydrolysis was measured by HPLC and/or the degree of RNA hybridization was separately measured by UV absorbance. The reaction containing both RNA and depsipeptide exhibited substantially higher levels of both RNA hybridization (due to increased *T*_m_) and intact depsipeptide **9** (due to reduced backbone ester hydrolysis) (Fig. 5f). Thus, non-covalent interactions between the RNA and the depsipeptide had increased the lifetime of the covalent depsipeptide backbone and increased the stability of the duplex form of RNA relative to single strands.

## Discussion

We are exploring the hypothesis that the relationship between RNA and peptide extends back into early chemical evolution, to an era when proto-peptides with heterogeneous backbones (polyesters and/or depsipeptides) interacted with proto-nucleic acids. Here, we have studied the potential for cationic proto-peptide mixtures generated in plausibly prebiotic dry-down reactions to engage in direct interactions with RNA. We found that such interactions can increase the lifetimes of the proto-peptides by decreasing their rate of degradation, and can stabilize the RNA by increasing the melting temperature of folds and assemblies. Cationic depsipeptides^39^ and polyesters can form robustly via simple dry-down reactions in the absence of condensing agents (Fig. 1). We also synthesized a library of model cationic depsipeptides containing one to eight ester bonds to facilitate mechanistic and structure–function relationship studies (Table 1). Proto-peptides containing proteinaceous amino acids Arg, Lys, and His adjacent to ester bonds generally promoted RNA duplex thermal stability to a greater magnitude than did analogous sequences containing the non-proteinaceous residues Orn, Dab, or Dpr (Figs. 2 and 3 and Table 1). In turn, interactions between RNA and a depsipeptide could dramatically increase the hydrolytic stability of the depsipeptide backbone (Fig. 5). Thus, RNA–depsipeptide interactions can mutually prolong the lifetime of the depsipeptide backbone and stabilize the fold adopted by the RNA.

Undoubtedly, many factors would have influenced the selection processes that eventually led to the set of proteinaceous amino acids. These factors would include availabilities of various building blocks on the ancient Earth^46^, efficiencies of non-enzymatic incorporation into growing oligomers, and the relative abilities of different side chains to impart functions within the context of the oligomer^47^. It is generally believed that the cationic amino acids found in proteins (Lys, Arg, and His) were not abundant on prebiotic Earth, although Lys has been observed in meteorites^48,49^, and potentially-prebiotic routes for both Arg and His have been proposed^26,50^. Amino acids with shorter cationic side chains, such as Orn, Dab, and Dpr, have also been observed in meteorites and model prebiotic reactions, and are thought to have been more abundant on prebiotic Earth^51–55^. It is intriguing that proto-peptide oligomers containing Arg, His, and Lys generally promoted greater increases in RNA duplex thermal stability than those containing Orn, Dab, or Dpr, both as product mixtures from dry-down reactions (Fig. 2) and as pure, synthetic molecules (Fig. 3, Table 1, Supplementary Table 1). We recently showed that the proteinaceous cationic amino acids Arg, His, and Lys also oligomerized with higher efficiencies and regioselectivities than non-proteinaceous analogs Orn, Dab, and Dpr^39^. Thus, not only are the proteinaceous cationic amino acids chemically predisposed to produce longer linear oligomers than Orn, Dab, or Dpr, they can also generate depsipeptides that are superior in terms of stabilizing RNA structures, even in otherwise identical sequences differing only in the cationic side chain. Mechanistically, it appears that the same underlying factors lead to both the different efficiencies of oligomerization and the different degrees of stabilization of RNA duplexes. The side chains of Arg, His, and Lys have an inherently lower likelihood of undergoing intramolecular reactions via O,N acyl transfer compared to Orn, Dab, or Dpr, reducing the chances of chain-terminating side reactions during oligomerization^39^ and degradation of the product oligomer in aqueous solution, which erodes the effectiveness of RNA binding/stabilization.

Mutually stabilizing interactions of the type described here might have provided important benefits to certain prebiotic systems. In a background of periodic, random (non-coded and non-templated) condensation processes, mutually stabilizing interactions could naturally lead to a buildup of those oligomers that possessed increased lifetimes due to the interactions. Shielding RNA against denaturation could increase its robustness and the range of environments in which it was folded and functional. Additional mechanisms have been put forth by which interactions between peptides and RNA could promote synthesis, stabilization, or localization of prebiotic oligomers. In one example, cationic poly(Leu-Lys) peptides were reported to encourage the oligomerization of activated mononucleotide diphosphates, and were especially effective in increasing the abundance of the longer RNA oligomers^56,57^. RNA can template the native chemical ligation of cationic peptide fragments derived from a biological protein-binding partner^23,58^. In the context of ribonucleotide-amino acid copolymers, the phosphoramidate and ester linkages within the co-oligomer backbone can be mutually stabilized against hydrolysis^59^. Short, cationic, amphiphilic peptides can localize RNA to the surface of protocell membranes^60^. Collectively, these findings suggest that interactions between RNA and cationic peptides/proto-peptides could mutually support synthesis and stabilization of both classes of molecules.

In addition to the vast span of known beneficial interactions between RNA and cationic peptides, it is worth mentioning that certain interactions between cationic oligomers and RNA could have a reverse effect and could have negatively impacted the lifetimes or functions of molecules involved in the interaction. For example, it is known that interactions between cationic peptides and RNA can accelerate RNA degradation^61–65^ or cause RNA aggregation^66^. For instance, Brack and colleagues studied a series of (Leu-Lys)_*n*_ or (Leu-Lys-Lys-Leu)_*n*_ peptides of varying lengths and degrees of stereochemical purity, and found that they exhibited RNA hydrolysis activities that correlated with the degree of β-sheet or α-helical character in the peptides. Considering that depsipeptides and polyesters should have less β-sheet or α-helical character compared to pure peptides (due to missing hydrogen bond donors and weakened hydrogen bond acceptors), it is possible that catalysis of RNA hydrolysis by proto-peptides would be attenuated compared to peptides. Another consideration is that the formation of complexes between proto-peptides and RNA could impair chemical processes that required conformational changes or unfolding of RNA structures, such as RNA catalysis or replication. An important caveat when considering interactions based primarily on electrostatics is that the strength of the molecular interactions would be modulated by changes in the ionic strength of the solution.

Investigations of biopolymer origins from a co-evolutionary perspective might afford valuable insights into early chemical evolution. The origins and evolution of biopolymers would have occurred amongst significant molecular heterogeneity, and productive cooperative interactions between molecules were almost certainly involved in the emergence, selection, and persistence of certain sets of molecules out of the clutter^67–69^. In principle, primordial molecular partnerships could have increased the lifetimes of certain molecules by numerous mechanisms. Our findings demonstrate that proto-peptides and early nucleic acids could have interacted in mutually stabilizing ways, but the processes demonstrated here involving increased thermal stability of noncovalent assemblies and increased backbone ester hydrolytic stability are only two possibilities out of many. Mutually stabilizing interactions provide a potential avenue for chemical selection—in mixtures containing multiple proto-peptide and RNA sequences, molecules with higher binding affinities could selectively associate in solution, persist, and adopt better-folded or more stable structures. We are currently investigating this possibility. Further studies of chemical systems in which productive interactions between different types of molecules could occur will likely lead to important insights in prebiotic chemistry and will be necessary for solving some of the enduring problems in the origin of life.

## Methods

### Peptide and depsipeptide synthesis

Peptides and depsipeptides were synthesized by using standard Fmoc chemistry with an Advanced Chemtech Apex 396 peptide synthesizer. A typical synthesis was performed on 0.09-mmol scale using Rink amide MBHA resin (~0.6 mmol/g). Standard side chain protecting groups included Lys(Boc), Orn(Boc), diaminopropionic acid(Boc), diaminobutyric acid(Boc), His(Trt), Arg(Pbf), Tyr(tBu), Glu(OtBu), and Asp(OtBu). Ester bonds were incorporated into the depsipeptides by coupling separately synthesized Fmoc-didepsipeptide building blocks that were appropriately protected on the side chain^40^. Chain elongations were carried out using 1,3-diisopropylcarbodiimide (DIC) and ethyl 2‐cyano‐2‐(hydroxyimino)acetate (oxyma) in *N*-methylpyrrolidin-2-one (NMP) with 75-min couplings. Fmoc deprotection was achieved using 2 × 8 min treatments with 25% 4-methylpiperidine in dimethylformamide (DMF). Washing steps involved 6 × 1 min treatments with DMF. Sequences were cleaved from the resin with concomitant side chain deprotection by agitation in a solution of 95:2.5:2.5 TFA:triisopropylsilane (TIS):water for 3 h. The crude products were precipitated with diethyl ether, centrifuged, and washed three additional times with ether. The crude peptides/depsipeptides were purified by preparative reverse-phase (RP)-HPLC on a Vydac 218TP C18 or Thermo BioBasic C18 column. Purity was confirmed by analytical RP-HPLC. Purified peptides were characterized by analytical HPLC and LC–MS. Analytical RP-HPLC was performed using a Zorbax 300-SB C-18 column connected to a Hitachi D-7000 HPLC system. Binary gradients of solvent A (99% H_2_O, 0.9% acetonitrile, 0.1% TFA) and solvent B (90% acetonitrile, 9.9% H_2_O, 0.07% TFA) were employed for HPLC.

In certain depsipeptide sequence contexts, such as…Dab-Ala-lac-Dab…, extensive truncation/termination was observed during the solid-phase synthesis, presumably due to context-dependent DKP formation. Accordingly, in these problematic sequences, the Dab residue located at the second position following incorporation of the ester bond (…**Dab**-Ala-lac-Dab…) was incorporated as a Bsmoc-protected amino acid rather than being Fmoc-protected, to enable its deprotection with lower concentrations of base^70^. After coupling Bsmoc-Dab(Boc)-OH to the growing depsipeptide, the Bsmoc group was removed using 3 × 1 min treatment with 2% 4-methylpiperidine/DMF. The subsequent DMF washing steps were limited to only 2 × 30 s to reduce the time that the free amine was present at N-terminus prior to the following coupling step^70^. These modified procedures eliminated observation of deletion/truncation products during the synthesis.

Stock solutions of peptides and depsipeptides were prepared in deionized water at 2 mM based on UV absorbance of Aba (*ε*_270_ = 17,394 M^−1^ cm^−1^) or Tyr (*ε*_280_ = 1,280 M^−1^ cm^−1^). RNA strands were purchased from Integrated DNA Technologies with standard desalting or HPLC-purification, and were dissolved in deionized water at 200 μM for stock solutions based on UV absorbance using the manufacturer-provided extinction coefficient.

### *T*_m_ analyses

Prior to *T*_m_ analyses, RNA samples were annealed in the appropriate buffer by heating to 75 °C and slowly cooling to room temperature. Depsipeptide/peptide was added to the annealed RNA and incubated at 4 °C for 20 min prior to carrying out the analysis. For analyses involving dry-down reaction mixtures, the *T*_m_ was determined by monitoring fluorescence increase upon melting of a fluorophore/quencher-labeled RNA duplex (5′-rCrGrArUrUrUrArGrCrG-/3IABkFQ/-3′ and 3′-rGrCrUrArArArUrCrGrC-FAM-5′) using a BioRad CFX Connect rtPCR instrument with a heating ramp of 1 °C/min and 20 μL final sample volumes. For analyses involving pure, synthetic compounds, the *T*_m_ of the RNA was determined by monitoring UV hyperchromicity at 260 nm using a Varian Cary Bio-100 spectrophotometer, with a heating ramp of 1 °C/min and 0.5- or 1-cm pathlength cells. The following RNA sequences were used: **RNA duplex 1** = 5′-rCrGrCrUrArArArUrCrG-3′ and 5′-rCrGrArUrUrUrArGrCrG-3′; **RNA duplex 2** = 5′-rArArArArUrUrUrArUrArUrUrArUrUrA-3′ and 5′-rUrArArUrArArUrArUrArArArUrUrUrU-3′; **RNA duplex 3** = 5′-rArArCrGrUrArUrArCrGrUrU-3′ (palindromic); and **H26a RNA** = 5′-rArUrGrArGrUrArArCrCrGrUrArArGrGrUrGrArArArUrU-3′. RNA was present in each assay at a final concentration of 2.5 μM of the folded structure (2.5 μM each strand for **duplex 1** and **duplex 2**, 5.0 μM palindromic strand for **duplex 3**, 2.5 μM strand for **H26a RNA**). *T*_m_ values were determined by nonlinear fitting of the melting curves using GraphPad Prism 8.2.1 for **duplex 1** and **H26a**, or by finding the maximum of the first derivative of the curve using the Varian Cary Bio-100 software for **duplex 2** and **duplex 3**. Both methods of determining the *T*_m_ gave similar values for a given denaturation curve.

### Dry-down reactions

Cationic depsipeptides were prepared by drying mixtures of hydroxy acids with cationic amino acids^39^. Aqueous solutions of either glc, lac, or isr with a single amino acid at a 5:1 molar ratio (in favor of the hydroxy acid) were allowed to dry at 85 °C under unbuffered, mildly acidic conditions (initial pH of ∼3) for 1 week. The amino acids were all used in their HCl form, and no additional salt was added to the reactions. Control reactions contained either a hydroxy acid alone or an amino acid alone. For formation of cationic polyesters and depsipeptides from cationic hydroxy acid monomers, aqueous solutions of either isr, hab or hah (100 μmol) were allowed to dry at 85 °C under unbuffered, mildly acidic conditions (initial pH of ∼3) for 1 week, either alone or in a binary mixture with Gly, Ala, lac, or glc. Before analysis, dry-down reaction mixtures were resuspended in ultrapure water to 100 mM concentration (based on original cationic hydroxy acid concentration or on original cationic amino acid concentration for co-dry-downs samples), vortexed, sonicated in ice, and centrifuged at 15,294 × *g* for 5 min. The supernatant was collected and diluted to the specified concentration. For dialysis, the lac- or glc-containing dry-down reaction mixtures were placed in a 500–1000 Da cut-off membrane (Micro Float-A-Lyzer, VWR #89219-388) and dialyzed against water.

### NMR spectroscopy

NMR spectra were recorded on a Bruker Avance II-500. To ensure quantitative integration of the resonances, relaxation delay of 15 s was used for dry-down reaction mixtures. Data were processed and spectra were plotted with MestReNova software package. The overall conversion of monomers into products was estimated from integration of the free, non-reacted α-proton ^1^H-NMR resonance. The extent of amidation at side-chain amines was quantified by integration of the resonance corresponding to methylene protons adjacent to the side-chain amine.

### LC–MS

LC–MS data were collected on an Agilent 1260 HPLC coupled to an Agilent 6130 single quadrupole mass spectrometer and an inline Agilent UV absorbance detector (210 nm) using a 3.0- or 3.5-kV electrospray ionization (ESI) capillary voltage. Samples were either directly infused into the mass spectrometer or separated using a Zorbax C18 column with a binary solvent system of water/acetonitrile/formic acid. Processing of MS data were conducted using a suite of macros using Igor Pro-8.0^39^.

### Band shift assay

For band shift analyses, 2.5 µL samples were prepared containing 40 µM of a 5′FAM-labeled 20-mer of polyuridylic acid (U20), a variable concentration of peptide or depsipeptide, and 100 mM MES-TEA, pH 6. An addition of 1.25 µL of glycerol brought the sample volume to a total of 3.75 µL. Samples were loaded onto a 12% native polyacrylamide gel, run at 100 V for 90 min, and imaged using Azure Biosystems c430.

### CD analysis

The CD spectra were collected at 4 °C using a Jasco J-815 CD instrument. Scans were from 320 nm to 220 nm with a bandwidth of 5 nm, data pitch of 0.2 nm, and scan rate of 50 nm/min with three accumulations. The 1 cm cuvette contained 5 μM RNA duplex (5′-rCrGrArUrUrUrArGrCrG-3′ and 3′-rGrCrUrArArArUrCrGrC-5′) and varying concentrations of the peptides/depsipeptides (0–10 μM) in a final volume of 600 μL in 60 mM MES-TEA buffer. The peptides/depsipeptides were added to the RNA sample and incubated for 10 min in ice prior to collection of the CD spectra. The consecutive addition of the peptides/depsipeptides did not result in any significant change in volume (the maximal change in overall volume was less than 2%). Data were collected in independent triplicates and the data were averaged. Finally, data were smoothed and plotted using Matlab.

### Depsipeptide hydrolysis assays and modeling

To determine the extent of depsipeptide hydrolysis, the depsipeptide (25 μM) was incubated at 37 °C in buffer (100 mM HEPES, 10 mM NaCl, pH 7.3) in the presence or absence of 10mer RNA **duplex 1** (5′-rCrGrArUrUrUrArGrCrG-3′ and 3′-rGrCrUrArArArUrCrGrC-5′) at various concentrations, or with single-stranded RNA (5′-rCrGrArUrUrUrArGrCrG-3′, 100 μM), for up to 100 h. Prior to adding depsipeptide to the reaction from its stock solution, the RNA was annealed by heating to 75 °C for 5 min, and then slowly cooling to room temperature. Reaction aliquots were quenched using 3% TFA and frozen or immediately analyzed by HPLC (270 nm). The relative concentrations of intact and hydrolyzed depsipeptide were quantified by integrating the corresponding peaks in HPLC traces. The kinetic profiles of depsipeptide hydrolysis were fit using SimFit-32 (provided by G. von Kiedrowski) according to the reaction scheme shown in Fig. 5a.

### Fitting of depsipeptide hydrolysis using SimFit

Data from the nine independent depsipeptide hydrolysis reactions carried out with different concentrations of RNA duplex were simultaneously fit using SimFit to the reaction model shown in Fig. 5a. In the model, the free depsipeptide is subject to hydrolysis at a pseudo-first order rate governed by *k*_hyd-free_. Binding of the depsipeptide to RNA according to an equilibrium determined by *k*_assoc_/*k*_dissoc_ yields a depsipeptide-RNA complex, for which the pseudo-first order rate of ester hydrolysis is described by *k*_hyd-complex_. During fitting, we fixed *k*_assoc_ = 1 × 10^5^ M^−1^ s^−1^, on the lower end of rates observed and expected for protein–RNA association^45^. Therefore, three parameters were variable during the fitting, and the final rate constants determined by the fitting were: *k*_hyd-free_ = 1.1 × 10^−4^ s^−1^, *k*_hyd-complex_ = 3.2 × 10^−6^ s^−1^, and *k*_dissoc_ = 8.3 × 10^−3^ s^−1^. It should be noted that the fixed value used for *k*_assoc_ did not affect the final values calculated for *k*_hyd-free_ or *k*_hyd-complex_ as long as *k*_assoc_ was greater than 1 × 10^3^ M^−1^ s^−1^, because the *k*_dissoc_ value adjusted accordingly during the data fitting to compensate for different *k*_assoc_ values. When *k*_assoc_ was fixed at values below 1 × 10^3^ M^−1^ s^−1^, the root mean square error values for the fit began to increase, indicating that the actual *k*_assoc_ > 1 × 10^3^ M^−1^ s^−1^. Our model assumes a 1:1 stoichiometry for depsipeptide:RNA in the complex, and assumes the RNA is present only as a duplex (ignores steps involving RNA strand hybridization to form the duplex). The final RMS error of the fit was 5.4%. The output file obtained from the fitting is provided in the Supplementary Note.

### Statistics and reproducibility

The experiments described in this paper were repeated independently at least two times. All attempts at experimental replication were successful. Graphical data are shown as scatter plots with all data points included. No data were excluded from the analyses.

### Reporting summary

Further information on research design is available in the Nature Research Reporting Summary linked to this article.

## Supplementary information

Supplementary Information

Reporting Summary

## Data Availability

All the data supporting the findings of this study are available within the main text and the Supplementary Information. Data are also available from the corresponding authors upon request.
