# Supplementary material for: Mutually stabilizing interactions between proto-peptides and RNA
Source: Nat Commun. 2020 Jun 19;11:3137. doi: 10.1038/s41467-020-16891-5 (PMC7305224; doi:10.1038/s41467-020-16891-5)
Supplement: Supplementary file 1 — Supplementary Information [file 41467_2020_16891_MOESM1_ESM.pdf]

Supplementary Information

Mutually Stabilizing Interactions Between Proto-Peptides and RNA

Moran Frenkel-Pinter[a,b,c], Jay W. Haynes[a,b], Ahmad M. Mohyeldin[a,b], Martin C[a,b], Alyssa B. Sargon[a,b], Anton S. Petrov[a,b,c], Ramanarayanan Krishnamurthy[a,d], Nicholas V. Hud[a,b], Loren Dean Williams[a,b,c]* and Luke J. Leman[a,d]*

[a] NSF/NASA Center for Chemical Evolution (USA)

[b] School of Chemistry & Biochemistry, Georgia Institute of Technology, Atlanta, GA 30332 (USA)

[c] NASA Center for the Origins of Life, Georgia Institute of Technology, Atlanta, GA (USA)

[d] Department of Chemistry, The Scripps Research Institute, La Jolla, CA 92037 (USA)

*Co-corresponding authors

*Corresponding Authors:
Prof. Loren Dean Williams
School of Chemistry and Biochemistry
Georgia Institute of Technology
315 Ferst Drive NW
Atlanta, GA 30332-0400
Ph: (+1) (404) 385-6258
Fax: (+1) (404) 894-2295
loren.williams@chemistry.gatech.edu

Prof. Luke J. Leman
Department of Chemistry
The Scripps Research Institute
10550 North Torrey Pines Road
La Jolla, CA 92037
Ph: (+1) (858) 784-2711
Fax: (+1) (858) 784-2798
lleman@scripps.edu

Synthesis and characterization of protected building blocks

Fmoc-Lys(Boc)-lac-OBn

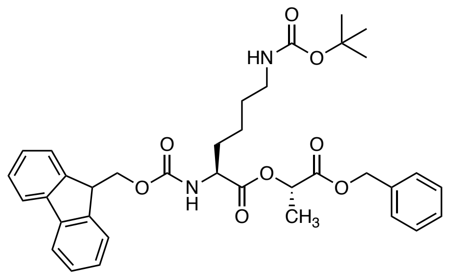

This compound was prepared with modification of a reported procedure[1]. Fmoc-Lys(Boc)-OH (10 mmol, 4.69 g, Aapptec) and Lac-OBn (11 mmol, 1.98 g, Alfa-Aesar) were dissolved in dry DCM (50 mL) and chilled in an ice bath. DMAP (1 mmol, 0.12 g) and oxyma (12 mmol, 1.72 g) were added to the cold mixture, followed by EDC (12 mmol, 2.3 g). The pale yellow solution was stirred and allowed to warm to room temperature overnight. TLC analysis indicated the reaction was complete by 24 h. The reaction mixture was diluted with 100 mL DCM and then extracted 3x with saturated $NaHCO_3$, 2x with saturated $KHSO_4$, and 1x with brine. The organic layer was dried over anhydrous $MgSO_4$ and evaporated to a yellow oil (7.9 g). The crude product was purified by flash chromatography using 200 g silica in a 60 cm diameter column, with 30% EtOAc/hexanes as the eluent. A clear oil (4.9 g, 78%) was obtained after evaporation of the solvent. NMR and MS matched previously reported values[1].

Fmoc-Lys(Boc)-lac-OH

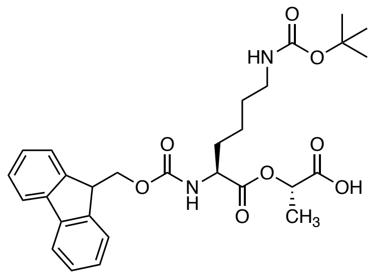

This compound was prepared by a modification of a reported procedure[1]. Fmoc-Lys(Boc)-lac-OBn (4.9 g, 7.77 mmol) was dissolved in 100 mL EtOH (200 proof) with Pd/C (1.0 g, 5 wt%, wet support, Degussa E101 NOW, Aldrich). Hydrogen gas was bubbled into the flask from a balloon. The progress of the reaction was tracked by TLC (5% MeOH/DCM) and was complete at 30 min. To prevent possible removal of the Fmoc group under the conditions of hydrogenation, the reaction was immediately filtered through celite. After evaporating the solvent, the residue was taken up in 100 mL EtOAc and extracted 4x with pH 4 water (prepared by mixing sat. $KHSO_4$ and sat. $NaHCO_3$) to remove residual lactic acid. The resulting organic layer was dried over anhydrous $MgSO_4$ and evaporated to a white solid (3.85 g, 91%). NMR and MS matched previously reported values[1].

Fmoc-Orn(Boc)-lac-OBn

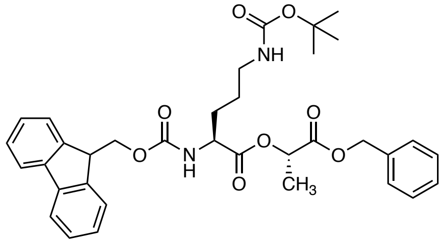

Fmoc-Orn(Boc)-OH (10 mmol, 4.54 g, Aapptec) and Lac-OBn (11 mmol, 1.98 g, Alfa-Aesar) were dissolved in dry DCM (50 mL) and chilled in an ice bath. DMAP (1 mmol, 0.12 g) and oxyma (12 mmol, 1.72 g) were added to the cold mixture, followed by EDC (14 mmol, 2.68 g). The pale reddish solution was stirred and allowed to warm to room temperature overnight. TLC analysis indicated the reaction was complete by 24 h. The reaction mixture was diluted with 100 mL DCM and then extracted 4x with saturated $NaHCO_3$, 2x with saturated $KHSO_4$, and 1x with brine. The organic layer was dried over anhydrous $MgSO_4$ and evaporated to a yellow oil. The crude product was purified by flash chromatography using 200 g silica in a 60 cm diameter column, with 30% EtOAc/hexanes as the eluent. A white solid (5.07 g, 82%) was obtained after evaporation of the solvent.

^{1}H NMR (500 MHz, DMSO-d6): δ 7.91-7.89 (d, 2H), 7.84-7.82 (d, 1H), 7.73-7.71 (d, 2H), 7.44-7.31 (m, 9H), 6.78-6.76 (t, 1H), 5.16-5.09 (m, 3H), 4.30-4.28 (m, 2H), 4.24-4.20 (m, 1H), 4.10-4.05 (m, 1H), 2.92-2.88 (m, 2H), 1.78-1.73 (m, 1H), 1.66-1.38 (m, 15H).

^{13}C NMR (126 MHz, DMSO-d6): δ 172.53, 170.44, 156.60, 156.07, 144.28, 144.20, 141.20, 135.99, 128.94, 128.68, 128.40, 128.33, 128.12, 127.54, 125.72, 120.61, 77.89, 69.20, 66.78, 66.19, 53.86, 47.08, 28.74, 28.25, 26.48, 17.13.

ESI-MS (m/z): $[MNa]^+$ calcd. for $C_{35}H_{40}N_2O_8Na$, 639.27; found, 639.3.

Fmoc-Orn(Boc)-lac-OH

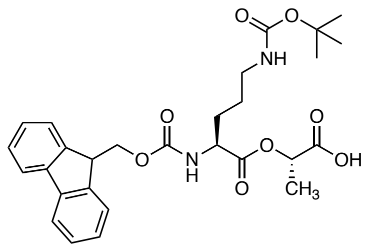

Fmoc-Orn(Boc)-lac-OBn (5.07 g, 8.22 mmol) was suspended in 80 mL EtOH (200 proof). Gentle heating and addition of ~10 mL EtOAc was required to aid dissolution. The solution was stirred with Pd/C (0.8 g, 5 wt%, wet support, Degussa E101 NOW, Aldrich) under an atmosphere of hydrogen gas bubbled into the flask from a balloon. The starting material was not completely soluble in the reaction solvent, but the solid disappeared over the course of the reaction as it was converted to product. The progress of the reaction was tracked by TLC (5% MeOH/DCM) and was complete at 30 min. To prevent possible removal of the Fmoc group under the conditions of hydrogenation, the reaction was immediately filtered

through celite. After evaporating the solvent, the residue was taken up in 100 mL EtOAc and extracted 4x with pH 4 water (prepared by mixing sat. $KHSO_4$ and sat. $NaHCO_3$) to remove residual lactic acid. The resulting organic layer was dried over anhydrous $MgSO_4$ and evaporated to a white solid (3.84 g, 89%).

^{1}H NMR (500 MHz, DMSO-d6): δ 13.02 (br s, 1H), 7.91-7.89 (d, 2H), 7.82-7.80 (d, 1H), 7.74-7.71 (m, 2H), 7.44-7.41 (m, 2H), 7.36-7.31 (m, 2H), 6.79-6.77 (br t, 1H), 4.96-4.92 (q, 1H), 4.33-4.22 (m, 3H), 4.10-4.05 (m, 1H), 2.96-2.90 (m, 2H), 1.84-1.74 (m, 1H), 1.63-1.45 (m, 3H), 1.41-1.38 (m, 12H).

^{13}C NMR (126 MHz, DMSO-d6): δ 172.45, 172.03, 156.61, 156.08, 144.31, 144.21, 141.20, 128.12, 127.55, 125.74, 120.60, 77.89, 69.15, 66.18, 53.87, 47.09, 28.75, 28.23, 26.50, 17.18.

HRMS (m/z): $[MH]^+$ calcd. for $C_{28}H_{35}N_2O_8$, 527.2393; found, 527.2391.

Fmoc-Dab(Boc)-lac-OBn

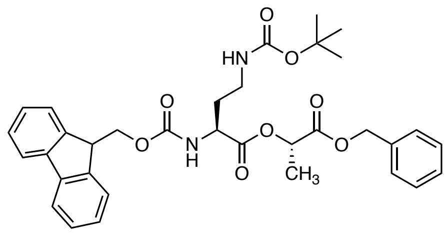

Fmoc-Dab(Boc)-OH (10 mmol, 4.40 g, Combi-Blocks) and Lac-OBn (11 mmol, 1.98 g, Alfa-Aesar) were dissolved in dry DCM (50 mL) and chilled in an ice bath. DMAP (1 mmol, 0.12 g) and oxyma (12 mmol, 1.72 g) were added to the cold mixture, followed by EDC (14 mmol, 2.68 g). The pale yellow solution was stirred and allowed to warm to room temperature overnight. TLC analysis indicated the reaction was complete by 24 h. The reaction mixture was diluted with 100 mL DCM and then extracted 4x with saturated $NaHCO_3$, 2x with saturated $KHSO_4$, and 1x with brine. The organic layer was dried over anhydrous $MgSO_4$ and evaporated to a yellow oil. The crude product was purified by flash chromatography using 200 g silica in a 60 cm diameter column, with 30% EtOAc/hexanes as the eluent. A white solid (4.70 g, 78%) was obtained after evaporation of the solvent.

^{1}H NMR (500 MHz, DMSO-d6): δ 7.91-7.86 (m, 3H), 7.74-7.71 (m, 2H), 7.44-7.31 (m, 9H), 6.89-6.87 (t, 1H), 5.16-5.10 (m, 3H), 4.29-4.20 (m, 3H), 4.16-4.11 (m, 1H), 3.08-2.98 (m, 2H), 1.99-1.94 (m, 1H), 1.71-1.68 (m, 1H), 1.44-1.42 (d, 3H), 1.38 (s, 9H).

^{13}C NMR (126 MHz, DMSO-d6): δ 172.49, 170.45, 156.60, 156.03, 144.28, 144.22, 141.19, 136.00, 128.94, 128.67, 128.36, 128.13, 127.56, 125.73, 120.60, 78.04, 69.24, 66.78, 66.26, 51.89, 47.07, 37.25, 31.11, 28.71, 17.11.

ESI-MS (m/z): $[MNa]^+$ calcd. for $C_{34}H_{38}N_2O_8Na$, 625.25; found, 625.3.

Fmoc-Dab(Boc)-lac-OH

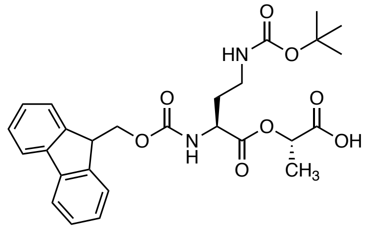

Fmoc-Dab(Boc)-lac-OBn (4.70 g, 7.80 mmol) was dissolved with gentle heating in 80 mL EtOH (200 proof). The solution was stirred with Pd/C (0.8 g, 5 wt%, wet support, Degussa E101 NOW, Aldrich) under hydrogen atmosphere bubbled into the flask from a balloon. The progress of the reaction was tracked by TLC (5% MeOH/DCM) and was complete at 30 min. To prevent possible removal of the Fmoc group under the conditions of hydrogenation, the reaction was immediately filtered through celite. After evaporating the solvent, the residue was taken up in 100 mL EtOAc and extracted 4x with pH 4 water (prepared by mixing sat. $KHSO_4$ and sat. $NaHCO_3$) to remove residual lactic acid. The resulting organic layer was dried over anhydrous $MgSO_4$ and evaporated to a white solid (3.68 g, 92%).

^{1}H NMR (500 MHz, DMSO-d6): δ 13.05 (br s, 1H), 7.91-7.89 (d, 2H), 7.85-7.84 (d, 1H), 7.74-7.72 (m, 2H), 7.44-7.41 (m, 2H), 7.36-7.32 (m, 2H), 6.91-6.88 (br t, 1H), 4.97-4.93 (q, 1H), 4.27-4.20 (m, 3H), 4.15-4.10 (m, 1H), 3.11-2.99 (m, 2H), 2.02-1.98 (m, 1H), 1.75-1.69 (m, 1H), 1.41-1.37 (m, 12H).

^{13}C NMR (126 MHz, DMSO-d6): δ 172.43, 172.06, 156.61, 156.04, 144.25, 141.19, 128.12, 127.56, 125.75, 120.60, 78.03, 69.18, 66.25, 51.92, 47.07, 37.31, 31.13, 28.71, 17.17.

HRMS (m/z): $[MH]^+$ calcd. for $C_{27}H_{33}N_2O_8$, 513.2237; found, 513.2239.

Fmoc-Arg(Pbf)-lac-OBn

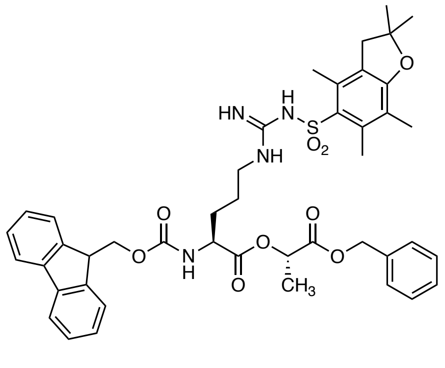

Fmoc-Arg(Pbf)-OH (10 mmol, 6.49 g, Aapptec) and Lac-OBn (33 mmol, 5.946 g, Alfa-Aesar) were dissolved in dry DCM (50 mL) and chilled in an ice bath. The excess amount of of Lac-OBn was necessary to reduce a side reaction involving intramolecular reaction of the Arg side chain with the activated carboxyl group. DMAP (1 mmol, 0.12 g) and oxyma (12 mmol, 1.72 g) were added to the cold mixture, followed by EDC (15.8 mmol, 3.03 g). The pale yellow solution was stirred and allowed to warm to room temperature overnight. TLC analysis indicated the reaction was complete by 16 h. The reaction mixture was diluted

with 100 mL DCM and then extracted 4x with saturated $NaHCO_3$, 2x with saturated $KHSO_4$, and 1x with brine. The organic layer was dried over anhydrous $MgSO_4$ and evaporated to a yellow oil. The crude product was purified by flash chromatography using 200 g silica in a 60 cm diameter column, with 60% EtOAc/hexanes as the eluent. Under these conditions, the undesired intramolecular side product (Rf = 0.6) could be cleanly separated from the desired product (Rf = 0.3), which was obtained as a white solid (4.10 g, 51%) after evaporation of the solvent.

^{1}H NMR (500 MHz, DMSO-d6): δ 7.90-7.84 (m, 3H), 7.72-7.70 (m, 2H), 7.44-7.30 (m, 10H), 5.15-5.10 (m, 3H), 4.34-4.28 (m, 2H), 4.24-4.20 (m, 1H), 4.09-4.04 (m, 1H), 3.03-2.97 (m, 2H), 2.94 (s, 2H), 2.45 (s, 3H), 2.01 (s, 3H), 1.78-1.72 (m, 1H), 1.58-1.44 (m, 3H), 1.43 (d, 3H), 1.39 (s, 6H).

^{13}C NMR (126 MHz, DMSO-d6): δ 172.37, 170.44, 157.94, 156.59, 144.26, 144.18, 141.20, 137.75, 135.92, 131.92, 128.93, 128.69, 128.42, 128.12, 127.54, 125.70, 124.80, 120.59, 116.75, 86.75, 69.26, 66.83, 66.20, 53.85, 47.09, 42.94, 28.73, 19.44, 18.08, 17.11, 12.74.

ESI-MS (m/z): $[MH]^+$ calcd. for $C_{44}H_{51}N_4O_9S$, 811.33; found, 811.2.

Fmoc-Arg(Pbf)-lac-OH

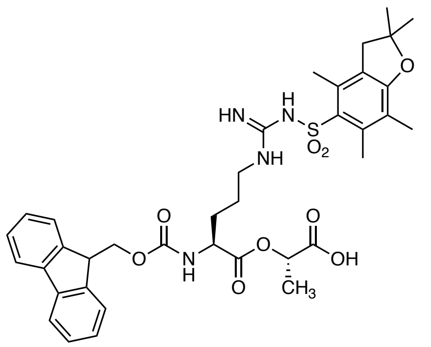

Fmoc-Arg(Pbf)-lac-OBn (4.10 g, 5.05 mmol) was dissolved with gentle heating in 70 mL of a 2:5 mixture of EtOAc/EtOH (200 proof). The solution was stirred with Pd/C (0.5 g, 5 wt%, wet support, Degussa E101 NOW, Aldrich) under hydrogen atmosphere bubbled into the flask from a balloon. The starting material was not completely soluble in the reaction solvent, but the solid disappeared over the course of the reaction as it was converted to product. The progress of the reaction was tracked by TLC (5% MeOH/DCM) and was complete at 55 min. To prevent possible removal of the Fmoc group under the conditions of hydrogenation, the reaction was immediately filtered through celite. The solvent was evaporated to a white foam (3.25 g, 89%).

^{1}H NMR (500 MHz, DMSO-d6): δ 7.90-7.89 (d, 2H), 7.84-7.82 (d, 1H), 7.73-7.70 (m, 2H), 7.44-7.31 (m, 5H), 4.96-4.92 (m, 1H), 4.34-4.21 (m, 3H), 4.08-4.03 (m, 1H), 3.08-3.04 (m, 2H), 2.95 (s, 2H), 2.50 (s, 3H), 2.44 (s, 3H), 2.01 (s, 3H), 1.83-1.78 (m, 1H), 1.61-1.46 (m, 3H), 1.41-1.40 (m, 9H).

^{13}C NMR (126 MHz, DMSO-d6): δ 172.28, 172.02, 157.92, 156.59, 144.29, 144.19, 141.20, 137.74, 131.90, 128.12, 127.54, 125.73, 124.80, 120.60, 116.73, 86.76, 69.21, 66.18, 53.83, 47.10, 42.93, 28.75, 19.43, 18.07, 17.17, 12.74.

HRMS (m/z): $[MH]^+$ calcd. for $C_{37}H_{45}N_4O_9S$, 721.2907; found, 721.2910.

Fmoc-His(Trt)-lac-OBn

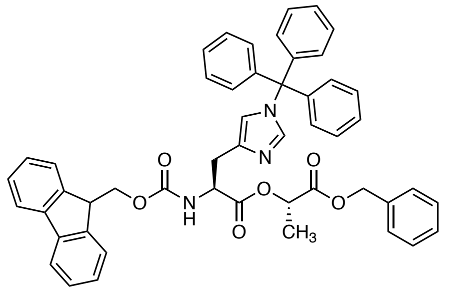

Fmoc-His(Trt)-OH (12 mmol, 7.44 g, Aapptec) and Lac-OBn (11 mmol, 1.98 g, Alfa-Aesar) were dissolved in dry DCM (25 mL) and chilled in an ice bath. DMAP (1.1 mmol, 0.13 g) was added to the cold mixture, followed by EDC (13 mmol, 2.49 g). The pale yellow solution was stirred and allowed to warm to room temperature overnight. TLC analysis indicated the reaction was complete by 16 h. The solvent was evaporated, and the crude reaction mixture was taken up in 150 mL EtOAc and then extracted 3x with saturated $NaHCO_3$, 3x with saturated $KHSO_4$, and 1x with brine. The organic layer was dried over anhydrous $MgSO_4$ and evaporated to a white solid. The crude product was purified by flash chromatography using 350 g silica in a 75 cm diameter column, with 2.5% MeOH/DCM as the eluent. The desired product was obtained as a white solid (6.66 g, 77%) after evaporation of the solvent.

^{1}H NMR (500 MHz, DMSO-d6): δ 7.91-7.89 (d, 2H), 7.84-7.82 (d, 1H), 7.67-7.66 (d, 2H), 7.43-7.26 (m, 18H), 7.07-7.04 (m, 7H), 6.82 (br s, 1H), 5.16-5.10 (m, 3H), 4.47-4.42 (m, 1H), 4.32-4.28 (m, 1H), 4.25-4.16 (m, 2H), 3.04-3.00 (m, 1H), 2.84-2.79 (m, 1H), 1.42-1.41 (d, 3H).

^{13}C NMR (126 MHz, DMSO-d6): δ 171.74, 170.38, 156.40, 144.16, 142.32, 141.19, 138.05, 135.91, 129.71, 128.93, 128.69, 128.59, 128.37, 128.33, 128.14, 127.56, 127.53, 125.65, 120.61, 120.30, 75.54, 69.27, 66.83, 66.28, 53.99, 47.06, 29.43, 17.13.

ESI-MS (m/z): $[MH]^+$ calcd. for $C_{50}H_{44}N_3O_6$, 782.3; found, 782.5

Fmoc-His(Trt)-lac-OH

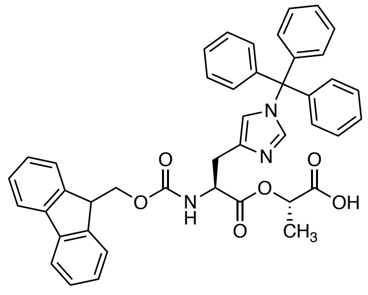

Fmoc-His(Trt)-lac-OBn (6.66 g, 8.52 mmol) was dissolved in 70 mL of a 2:5 mixture of EtOAc/EtOH (200 proof). The solution was stirred with Pd/C (0.6 g, 5 wt%, wet support, Degussa E101 NOW,

Aldrich) under a hydrogen atmosphere bubbled into the flask from a balloon. The progress of the reaction was tracked by TLC (5% MeOH/DCM) and was complete at 3 h. After evaporating the solvent, the residue was taken up in 100 mL EtOAc and extracted 4x with pH 5 water (prepared by mixing sat. $KHSO_4$ and sat. $NaHCO_3$) to remove residual lactic acid. The resulting organic layer was dried over anhydrous $MgSO_4$ and evaporated to a white solid. The product was purified by flash chromatography using 7% MeOH/DCM as eluent, which provided a white solid (3.42 g, 59%).

^{1}H NMR (500 MHz, DMSO-d6): δ 7.91-7.89 (d, 2H), 7.79-7.78 (d, 1H), 7.68-7.66 (m, 2H), 7.44-7.23 (m, 14H), 7.07-7.04 (m, 6H), 6.78-6.76 (m, 1H), 5.00-4.95 (q, 1H), 4.41-4.37 (m, 1H), 4.30-4.16 (m, 3H), 3.05-3.01 (dd, 1H), 2.86-2.81 (dd, 1H), 1.40-1.39 (d, 3H).

^{13}C NMR (126 MHz, DMSO-d6): δ 172.06, 171.90, 156.41, 144.19, 142.67, 141.18, 138.28, 136.74, 129.71, 128.61, 128.46, 128.12, 127.54, 125.68, 120.60, 119.94, 75.03, 69.29, 66.24, 55.38, 54.24, 47.07, 30.09, 17.24.

HRMS (m/z): $[MH]^+$ calcd. for $C_{43}H_{38}N_3O_6$, 692.2761; found, 692.2773.

Bsmoc-Dab(Boc)-OH

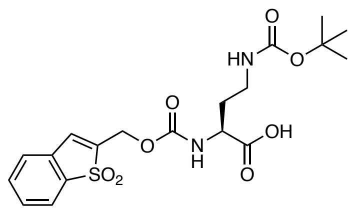

Synthesis of the title compound was adapted from a reported procedure[2]. H-Dab(Boc)-OH (1.0 g, 4.58 mmol, Combi-Blocks) was dissolved in 30 mL of a 1:1 mixture of water/acetonitrile. The pH was adjusted to ~9 using triethylamine. Bsmoc-OSu (1.568 g, 4.65 mmol, Alfa-Aesar) was added in a single portion, and additional triethylamine was added to keep the pH of the mixture ~9. The solid Bsmoc-OSu dissolved within a few minutes, and the pH stopped dropping after 10 min. The reaction was allowed to stir for an additional 40 min, after which the solution was acidified to pH 5 using 0.2 N HCl. The majority of solvent was removed (~5 mL remaining), and EtOAc (25 mL) was added. Additional 0.2 N HCl was added to lower the pH to ~2. The organic layer was removed, and the aqueous layer was extracted three additional times with EtOAc. The organic layers were combined, washed 3x with brine, and dried over anhydrous $MgSO_4$. Evaporation of the solvent, followed by flash chromatography using 4% MeOH/DCM containing 0.6% AcOH as eluent, provided a white solid (1.42 g, 70%).

^{1}H NMR (500 MHz, DMSO-d6): δ 12.41 (br s, 1H), 7.90-7.87 (m, 1H), 7.80-7.79 (d, 1H), 7.72-7.69 (m, 1H), 7.65-7.61 (m, 2H), 7.52-7.51 (m, 1H), 6.83-6.81 (br t, 1H), 5.05-4.97 (q, 2H), 4.00-3.96 (m, 1H), 3.03-2.98 (m, 2H), 1.91-1.85 (m, 1H), 1.73-1.66 (m, 1H), 1.37 (s, 9H).

^{13}C NMR (126 MHz, DMSO-d6): δ 174.03, 156.02, 155.87, 139.64, 136.99, 134.77, 131.32, 130.61, 130.49, 126.53, 121.82, 78.03, 56.18, 52.23, 37.43, 31.31, 28.71.

HRMS (m/z): $[MH]^+$ calcd. for $C_{19}H_{25}N_2O_8S$, 441.1332; found 441.1329

2-Hydroxy-6-aminohexanoic acid (hah)

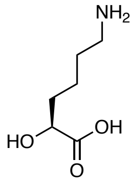

The title compound was synthesized based on a reported procedure[3]. L-Lysine·H_2O **1** (3.28 g, 20.0 mmol) was dissolved in 50 mL of 10% H_2SO_4. The solution was heated to 45 °C and a solution of $NaNO_2$ (5.18 g, 75.1 mmol) dissolved in 20 ml H_2O was added dropwise from a dropping funnel over a period of 2 h. The solution stirred an additional 17 h at 45 °C. TLC (7:2:1 isopropanol/ aq NH_4OH/H_2O, stained with ninhydrin) indicated the presence of remaining Lys, so an additional portion of $NaNO_2$ (2.0 g, 29.0 mmol) dissolved in 10 ml H_2O was added dropwise from a dropping funnel over a period of 1 h while the reaction was heated to 50 °C. TLC indicated complete consumption of Lys, and the reaction was quenched by addition of a solution of urea (7.0 g, 0.12 mol) dissolved in 20 ml of H_2O. The reaction mixture was stirred for 30 min, after which it was applied onto an ion exchange column of 75 mL amberlite IR-120 resin (H^+ form). The column was washed with 375 mL H2O, then product was eluted using a gradient of 5:1 to 2:1 H_2O/NH_4OH. Fractions containing the desired compound, as determined by TLC stained with ninhydrin, were concentrated under reduced pressure and lyophilized to yield a white solid (1.83 g) was obtained in 62% yield. NMR and MS matched the previously reported values[3].

^{1}H NMR (500 MHz, D_2O): δ 3.97-3.95 (dd, 1 H), 2.93-2.90 (t, 2H), 1.70-1.54 (m, 4 H), 1.43-1.26 (m, 2H).

^{13}C NMR (126 MHz, D_2O): δ 181.33, 71.85, 39.28, 33.19, 26.56, 21.24.

Supplementary References

1. Nguyen, M. M., Ong, N. & Suggs, L. A general solid phase method for the synthesis of depsipeptides. *Org. Biomolec. Chem.* **11**, 1167-1170 (2013).

2. Carpino, L. A. *et al.* The 1,1-dioxobenzo[b]thiophene-2-ylmethyloxycarbonyl (bsmoc) amino-protecting group. *J. Org. Chem.* **64**, 4324-4338 (1999).

3. Humenik, M., Huang, Y., Safronov, I. & Sprinzl, M. Simultaneous and site-directed incorporation of an ester linkage and an azide group into a polypeptide by in vitro translation. *Org. Biomolec. Chem.* **7**, 4218-4224 (2009).

Supplementary Note

Results output file from SimFit fitting (page 1 of 3):

```
**********************************************************************
* SimFit (PB/DLL6) 32-bit (10-Feb-2003)  (C) 1989-2001 G. v. Kiedrowski *
**********************************************************************
This version of SimFit comes with 2 GB variable space.
SF32.INI is currently set to:
63 species,63 reactions,24 iteratable rate constants,
16 observables,4 files,5 experiments/file,512 reaction times/experiment.

DEFINE (1,a,e,1)
SCALE (3,1)
a: K1 * FE
DEFINE (2,b,p,2)
SCALE (3,1)
b: K1 * FE
DEFINE (3,c,c,3)
SCALE (3,1)
c: K1 * FE
READ (hydrolysis_input_A.txt,hydrolysis_input_B.txt)
The following observables found in dep_hyd_1to5czero2.txt are DEFINEd:
a
b
c
Unit of time is not defined in data file.
SimFit assumes minutes as unit of time.
The following observables found in dep_hyd_6to9_2.txt are DEFINEd:
a
b
c
Unit of time is not defined in data file.
SimFit assumes minutes as unit of time.
SELECT (a,b,c,ac)
CHOOSE (expall)
TIME (min)
CONC (µm)
WIN (0,3600,1200,40,0,30,5,0.3)
REACTION (a --> b)
CONST (1,1.0e-04,1,1,100000)
REACTION (a + c ==> ac)
CONST (2,1.0e+5,0,1,100000)
CONST (3,7.9e-03,2,1,100000)
REACTION (ac --> b + c)
CONST (4,3.2e-6,3,1,100000)
REACTION (compile)
REACTION (show)
--------------------------------------
DGLCODER             Version 11.03.93
--------------------------------------
```

Results output file from SimFit fitting (page 2 of 3):

```
Reactions:
      A --> B
   A + C --> AC
     AC --> A + C
     AC --> B + C

Species found: A  B  C  AC

Rate equations:
   d[A]/dt =  - k1∑[A] - k2∑[A]∑[C] + k3∑[AC]
   d[B]/dt =  + k1∑[A] + k4∑[AC]
   d[C]/dt =  - k2∑[A]∑[C] + k3∑[AC] + k4∑[AC]
  d[AC]/dt =  + k2∑[A]∑[C] - k3∑[AC] - k4∑[AC]

Jacobian matrix: NJacobi& = 11 JacobiList& = 25
    d(d[A]/dt)/d[A] =  - k1 - k2∑[C]
    d(d[A]/dt)/d[C] =  - k2∑[A]
   d(d[A]/dt)/d[AC] =  + k3
    d(d[B]/dt)/d[A] =  + k1
   d(d[B]/dt)/d[AC] =  + k4
    d(d[C]/dt)/d[A] =  - k2∑[C]
    d(d[C]/dt)/d[C] =  - k2∑[A]
   d(d[C]/dt)/d[AC] =  + k3 + k4
   d(d[AC]/dt)/d[A] =  + k2∑[C]
   d(d[AC]/dt)/d[C] =  + k2∑[A]
  d(d[AC]/dt)/d[AC] =  - k3 - k4

ASSIGN (obs,a = a + ac)
ASSIGN (obs,b = b)
ASSIGN (obs,c = c + ac)
ASSIGN (spec,a = a)
ASSIGN (spec,b = b)
ASSIGN (spec,c = c)
DIM (3)
INTEGRATION (stiff,1e-6,3,0.1,300,1000)
PLOT (resi,spec)

SIMPLEX (plot)
k1_________ k2_________ k3_________ R.M.S.[%]
initial values:
  1.0000E-4   7.9000E-3   3.2000E-6    5.6387

  1.1056E-4   8.2072E-3   3.2533E-6    5.5600
  1.0233E-4   8.2889E-3   3.2370E-6    5.4988
  1.0272E-4   8.8804E-3   3.2432E-6    5.4939
  1.0698E-4   8.5715E-3   3.1202E-6    5.4451
  1.0728E-4   8.3937E-3   3.2267E-6    5.4278
```

<u>Results output file from SimFit fitting</u> (page 3 of 3):

1.0610E-4	8.5394E-3	3.1699E-6	5.4128
1.0543E-4	8.5106E-3	3.2037E-6	5.4117
1.0561E-4	8.5406E-3	3.2020E-6	5.4111
1.0604E-4	8.4977E-3	3.2017E-6	5.4102
1.0590E-4	8.5279E-3	3.1862E-6	5.4099
1.0611E-4	8.4873E-3	3.1900E-6	5.4097
1.0594E-4	8.5141E-3	3.1902E-6	5.4097
1.0592E-4	8.5059E-3	3.1943E-6	5.4096
1.0599E-4	8.5028E-3	3.1939E-6	5.4096
1.0603E-4	8.4975E-3	3.1914E-6	5.4096
1.0597E-4	8.5013E-3	3.1941E-6	5.4096
1.0601E-4	8.4953E-3	3.1947E-6	5.4096

sum squares = 8.8247E-11

NEWTON (1e9)
k1_________ k2_________ k3_________ R.M.S.[%]
Initial values:----------
1.0601E-4 8.4953E-3 3.1947E-6 5.4096
±0.0000E+0 ±0.0000E+0 ±0.0000E+0

1.0596E-4 8.2871E-3 3.1996E-6 5.4087
±1.1072E-6 ±7.2862E-4 ±4.5427E-8

1.0596E-4 8.2871E-3 3.1996E-6 5.4087
±1.1050E-6 ±7.1438E-4 ±4.5157E-8

sum squares = 8.8219E-11

Covariance Matrix:

	__k1__	__k2__	__k3__
k1	1.000	-0.217	0.047
k2	-0.217	1.000	-0.663
k3	0.047	-0.663	1.000

Supplementary Table 1. Observed RNA melting temperatures (T_m) in the presence of synthetic peptides and depsipeptides.

sequence	# cationic residues	RNA duplex 1 T_m (°C)	RNA duplex 2 T_m (°C)	RNA duplex 3 T_m (°C)	H26a RNA T_m (°C)
RNA alone (pH 7.0)	N/A	43.8 ± 0.4	33.7 ± 0.7	48.6 ± 0.7	53.4 ± 0.5
RNA alone (pH 5.0)	N/A	41.5 ± 0.3			
Ac-Lys-NH_2 (1 mM)	1	43.8 ± 0.1			
Ac-Tyr-Gly-Ala-Dab-Lys-NH_2 (400 mM)	2	43.7 ± 0.1	33.5 ± 0.3	47.5 ± 1.1	54.7 ± 0.4
Ac-Tyr-Gly-Ala-Dab-Lys-Ala-Dab-Lys-NH_2 (200 mM)	4	44.5 ± 0.9			
Ac-Tyr-Gly-Ala-Dab-Lys-Ala-Dab-Lys-Ala-Dab-Lys-NH_2 (133 mM)	6	47.2 ± 0.8			
Ac-Tyr-Gly-Ala-Dab-Lys-Ala-Dab-Lys-Ala-Dab-Lys-Ala-Dab-Lys-NH_2 (100 mM)	8	51.9 ± 0.2			62.9 ± 0.9
Ac-Tyr-Gly-Ala-Dab-Lys-**lac**-Dab-Lys-**lac**-Dab-Lys-**lac**-Dab-Lys-NH_2 (**14**)	8	52.4 ± 1.5	42.8 ± 0.6	58.6 ± 0.7	63.8 ± 0.6
Ac-Tyr-Gly-Ala-Dab-Lys-Ala-Dab-Lys-Ala-Dab-Lys-Ala-Dab-Lys-Ala-Dab-Lys NH_2 (80 mM)	10	53.4 ± 0.9			
Aba-Gly-Ala-Dab-Lys-Ala-Dab-Arg-**lac**-Dab-Lys-Ala-Dab-Lys-NH_2 (**7**)	8	52.5 ± 0.3	41.8 ± 0.6	60.6 ± 0.8	64.8 ± 0.1
Aba-Gly-Ala-Dab-Lys-Ala-Dab-His-**lac**-Dab-Lys-Ala-Dab-Lys-NH_2 (**8**) (pH 5.0)	8	52.6 ± 0.2			
Aba-Gly-Ala-Dab-Lys-Ala-Dab-His-**lac**-Dab-Lys-Ala-Dab-Lys-NH_2 (**8**) (pH 7.0)	8	48.9 ± 1.4			
Aba-Gly-Ala-Dab-Lys-Ala-Dab-Lys-**lac**-Dab-Lys-Ala-Dab-Lys-NH_2 (**9**)	8	54.2 ± 1.3	41.8 ± 0.6	58.5 ± 0.7	64.9 ± 0.7
Aba-Gly-Ala-Dab-Lys-Ala-Dab-Orn-**lac**-Dab-Lys-Ala-Dab-Lys-NH_2 (**10**)	8	48.7 ± 1.2	41.8 ± 0.5	51.1 ± 1.3	56.9 ± 0.1
Aba-Gly-Ala-Dab-Lys-Ala-Dab-Dab-**lac**-Dab-Lys-Ala-Dab-Lys-NH_2 (**11**)	8	48.7 ± 0.2			
Aba-Gly-Ala-Asp-Glu-Ala-Asp-Lys-**lac**-Asp-Glu-Ala-Asp-Glu-NH_2	-6	44.1 ± 0.1	34.3 ± 0.1	47.1 ± 1.4	54.5 ± 0.1
Aba-Arg-Ala-Arg-Ala-Arg-Ala-Arg-Ala-Arg-Ala-Arg-Ala-Arg-Ala-Arg-Ala-NH_2	8	49.7 ± 1.1			
Aba-Arg-**lac**-Arg-**lac**-Arg-**lac**-Arg-**lac**-Arg-**lac**-Arg-**lac**-Arg-**lac**-Arg-**lac**-NH_2 (**2**)	8	49.1 ± 0.7			
Aba-His-Ala-His-Ala-His-Ala-His-Ala-His-Ala-His-Ala-His-Ala-His-Ala-NH_2 (pH 5.0)	8	45.7 ± 0.1			
Aba-His-**lac**-His-**lac**-His-**lac**-His-**lac**-His-**lac**-His-**lac**-His-**lac**-His-**lac**-NH_2 (**3**) (pH 5.0)	8	46.1 ± 0.7			
Aba-His-**lac**-His-**lac**-His-**lac**-His-**lac**-His-**lac**-His-**lac**-His-**lac**-His-**lac**-NH_2 (**3**) (pH 7.0)	8	44.3 ± 1.0			
Ac-Tyr-Gly-Lys-Ala-Lys-Ala-Lys-Ala-Lys-Ala-Lys-Ala-Lys-Ala-Lys-Ala-Lys-Ala-NH_2	8	46.9 ± 0.3	36.6 ± 0.6	53.1 ± 0.1	57.5 ± 1.2
Aba-Lys-**lac**-Lys-**lac**-Lys-**lac**-Lys-**lac**-Lys-**lac**-Lys-**lac**-Lys-**lac**-Lys-**lac**-NH_2 (**4**)	8	46.4 ± 0.1	35.2 ± 0.1	52.5 ± 0.7	59.7 ± 0.6
Ac-Tyr-Gly-Orn-Ala-Orn-Ala-Orn-Ala-Orn-Ala-Orn-Ala-Orn-Ala-Orn-Ala-Orn-Ala-NH_2	8	48.3 ± 0.2	41.2 ± 0.2	56.9 ± 1.1	62.4 ± 1.6
Aba-Orn-**lac**-Orn-**lac**-Orn-**lac**-Orn-**lac**-Orn-**lac**-Orn-**lac**-Orn-**lac**-Orn-**lac**-NH_2 (**5**)	8	43.8 ± 0.9	34.6 ± 0.7	49.1 ± 0.1	52.3 ± 0.6
Ac-Tyr-Gly-Dab-Ala-Dab-Ala-Dab-Ala-Dab-Ala-Dab-Ala-Dab-Ala-Dab-Ala-Dab-Ala-NH_2	8	50.7 ± 0.7	41.6 ± 0.6	59.6 ± 0.6	63.6 ± 2.8
Aba-Dab-**lac**-Dab-**lac**-Dab-**lac**-Dab-**lac**-Dab-**lac**-Dab-**lac**-Dab-**lac**-Dab-**lac**-NH_2 (**6**)	8	44.2 ± 0.2	36.2 ± 0.1	48.2 ± 0.1	53.9 ± 0.2
Ac-Tyr-Gly-Dpr-Ala-Dpr-Ala-Dpr-Ala-Dpr-Ala-Dpr-Ala-Dpr-Ala-Dpr-Ala-Dpr-Ala-NH_2	8	44.7 ± 2.3			
5-FAM-Gly-Ala-Dab-Lys-Ala-Dab-Lys-Ala-Dab-Lys-Ala-Dab-Lys-Ala-Dab-Lys NH_2 (**12**) (80 mM)	10	54.4 ± 0.6			
5-FAM-Gly-Ala-Dab-Lys-Ala-Dab-Lys-Ala-Dab-Lys-**lac**-Dab-Lys-Ala-Dab-Lys NH_2 (**13**) (80 mM)	10	56.0 ± 0.8			
Ac-Tyr-Gly-Ala-Lys-Lys-Ala-Ala-Lys-Lys-Ala-Ala-Lys-Lys-Ala-Ala-Lys-Lys-Ala-NH_2	8	47.2 ± 0.2			
Ac-Tyr-Gly-Ala-Dab-Dab-Ala-Ala-Dab-Dab-Ala-Ala-Dab-Dab-Ala-Ala-Dab-Dab-Ala-NH_2	8	50.6 ± 0.4	42.5 ± 0.4	58.5 ± 0.5	61.8 ± 0.9
Ac-Tyr-Gly-Ala-Dab-Dab-Ala-**lac**-Dab-Dab-Ala-Ala-Dab-Dab-Ala-**lac**-Dab-Dab-Ala-NH_2	8	50.9 ± 0.1	40.8 ± 0.7	59.2 ± 0.2	64.2 ± 0.2
Ac-Tyr-Gly-Gly-Lys-Lys-Gly-Lys-Lys-Gly-Lys-Lys-Gly-Lys-Lys-NH_2	8	49.6 ± 0.4			
Ac-Tyr-Gly-Gly-Lys-Lys-Gly-Lys-Lys-Gly-Lys-Lys-Gly-Lys-Lys-NH_2	8	49.6 ± 0.1			
Ac-Tyr-Gly-Gly-Lys-Lys-Gly-Lys-Lys-Gly-Lys-Lys-Gly-Lys-Lys-NH_2	8	50.1 ± 1.0			

RNA **duplex 1** = 5'-rCrGrCrUrArArArUrCrG-3' & 5'-rCrGrArUrUrUrArGrCrG-3'; RNA **duplex 2** = 5'-rArArArArUrUrUrArUrArUrUrArUrUrA-3' and 5'-rUrArArUrArArUrArUrArArArUrUrUrU-3'; RNA **duplex 3** = 5'-rArArCrGrUrArUrArCrGrUrU-3' (palindromic); **H26a RNA** = 5'-rArUrGrArGrUrArArCrCrGrUrArArGrGrUrGrArArArUrU-3'. RNA was present in each assay at a final concentration of 2.5 μM of the folded structure (2.5 μM each strand for **duplex 1** and **duplex 2**, 5.0 μM palindromic strand for **duplex 3**, 2.5 μM strand for **H26a RNA**). Unless otherwise noted, the concentration of peptide/depsipeptide was 100 μM and the pH of the measurement was 7.0. Data represent the mean ± SD of 2-4 independent experiments. The buffers contained 10 mM phosphate, 100 mM NaCl at pH 7.0; or 10 mM acetate, 100 mM NaCl at pH 5.0. Aba = acetamidobenzoic acid, which was appended to the N-terminus of some sequences for improved UV absorbance. Underlined residues denote D-chirality.

Supplementary Figures

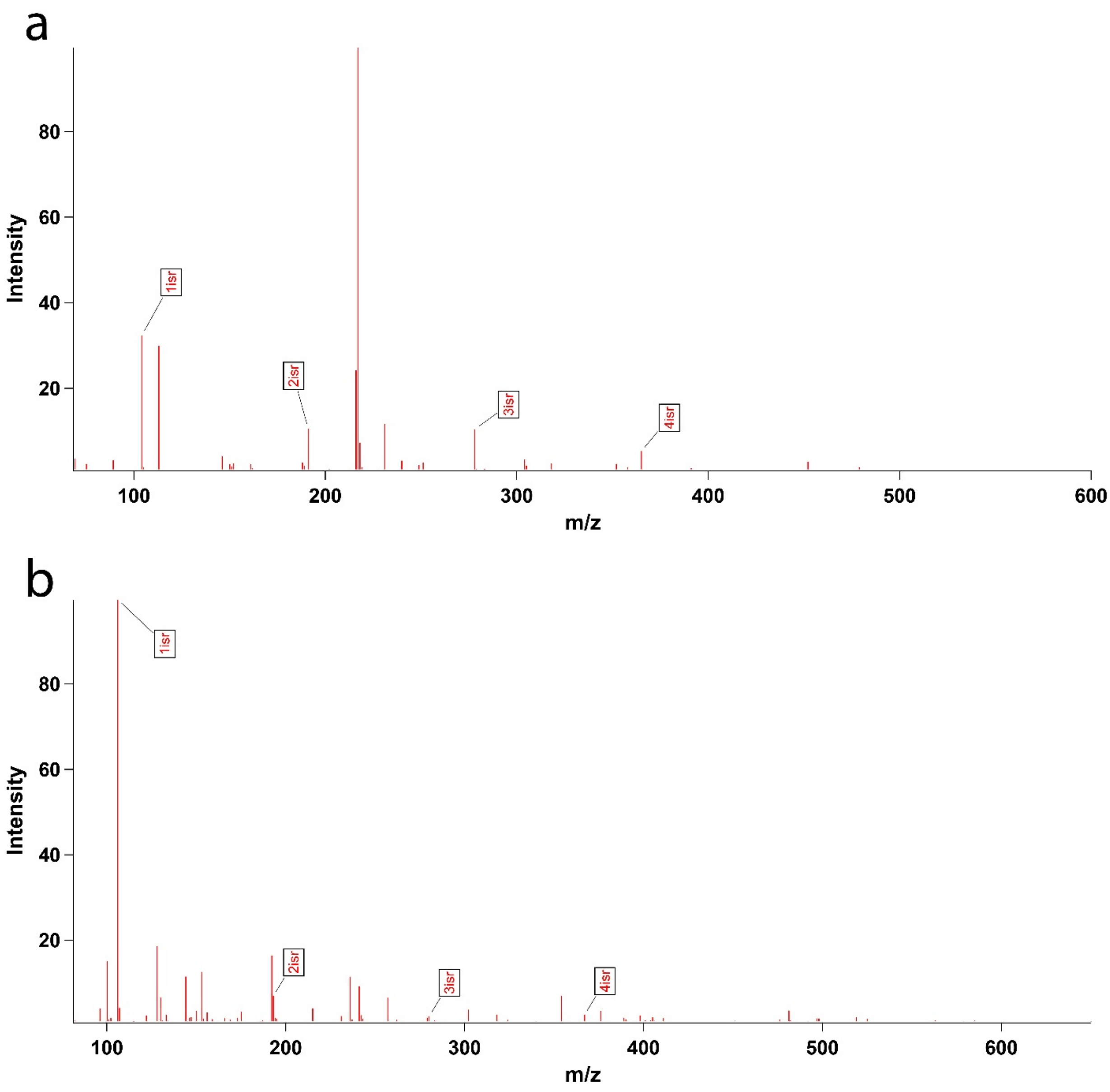

Supplementary Figure 1: ESI-MS of a dry-down reaction of isr supports the formation of polyesters/depsipeptides. **isr** was dried at 85 ˚C for seven days and the resulting polyesters/depsipeptides were analyzed by negative-mode ESI-MS (**a**) or positive mode ESI-MS (**b**), indicating a variety of product. Labeled species correspond to $[M-H]^-$ ions (**a**) or $[M+H]^+$ ions (**b**).

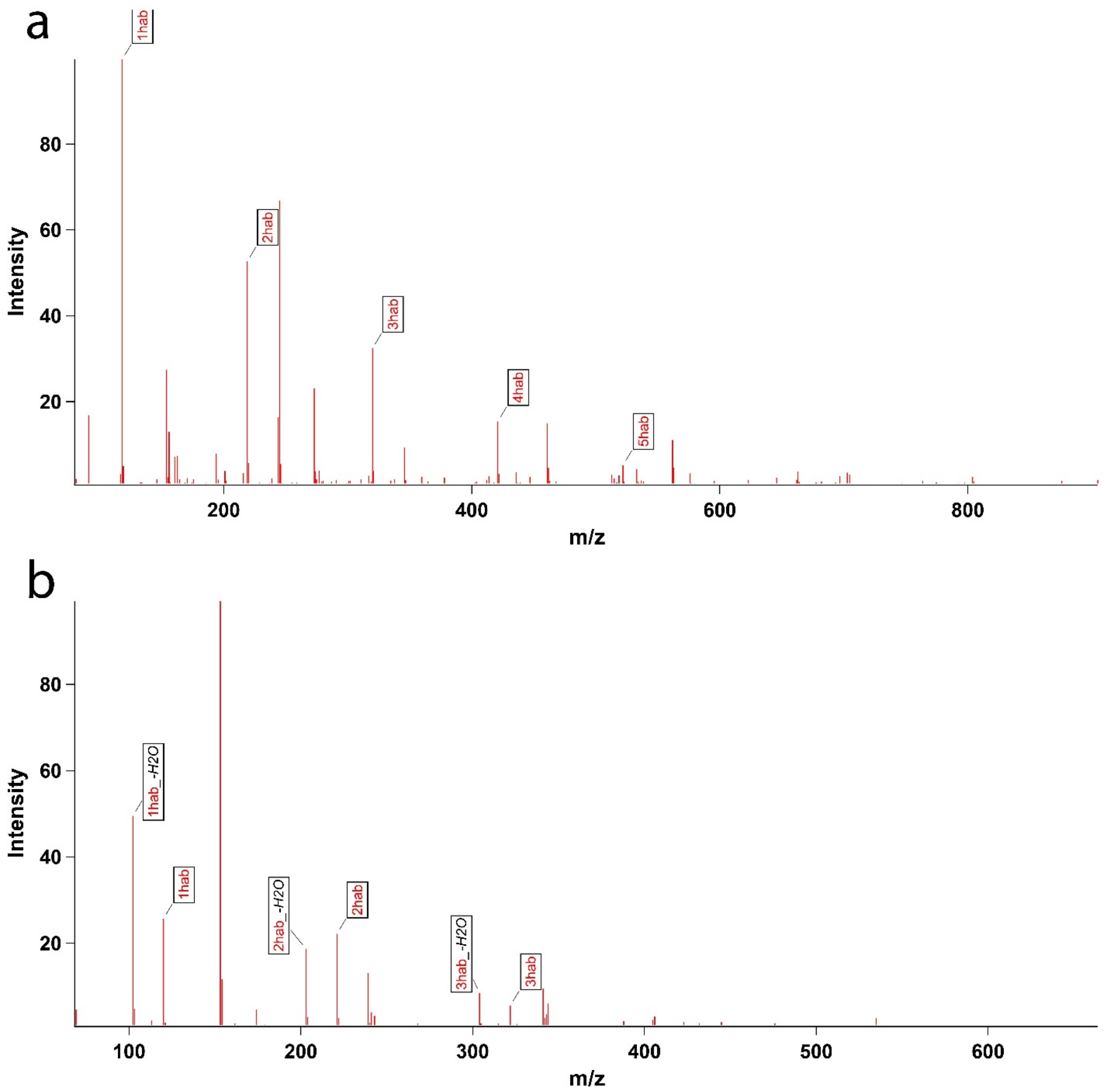

Supplementary Figure 2: ESI-MS of a dry-down reaction of hab supports the formation of polyesters/depsipeptides. **hab** was dried at 85 ˚C for seven days and the resulting polyesters/depsipeptides were analyzed by negative-mode ESI-MS (**a**) or positive mode ESI-MS (**b**), indicating a variety of product. Labeled species correspond to $[M-H]^-$ ions (**a**) or $[M+H]^+$ ions (**b**).

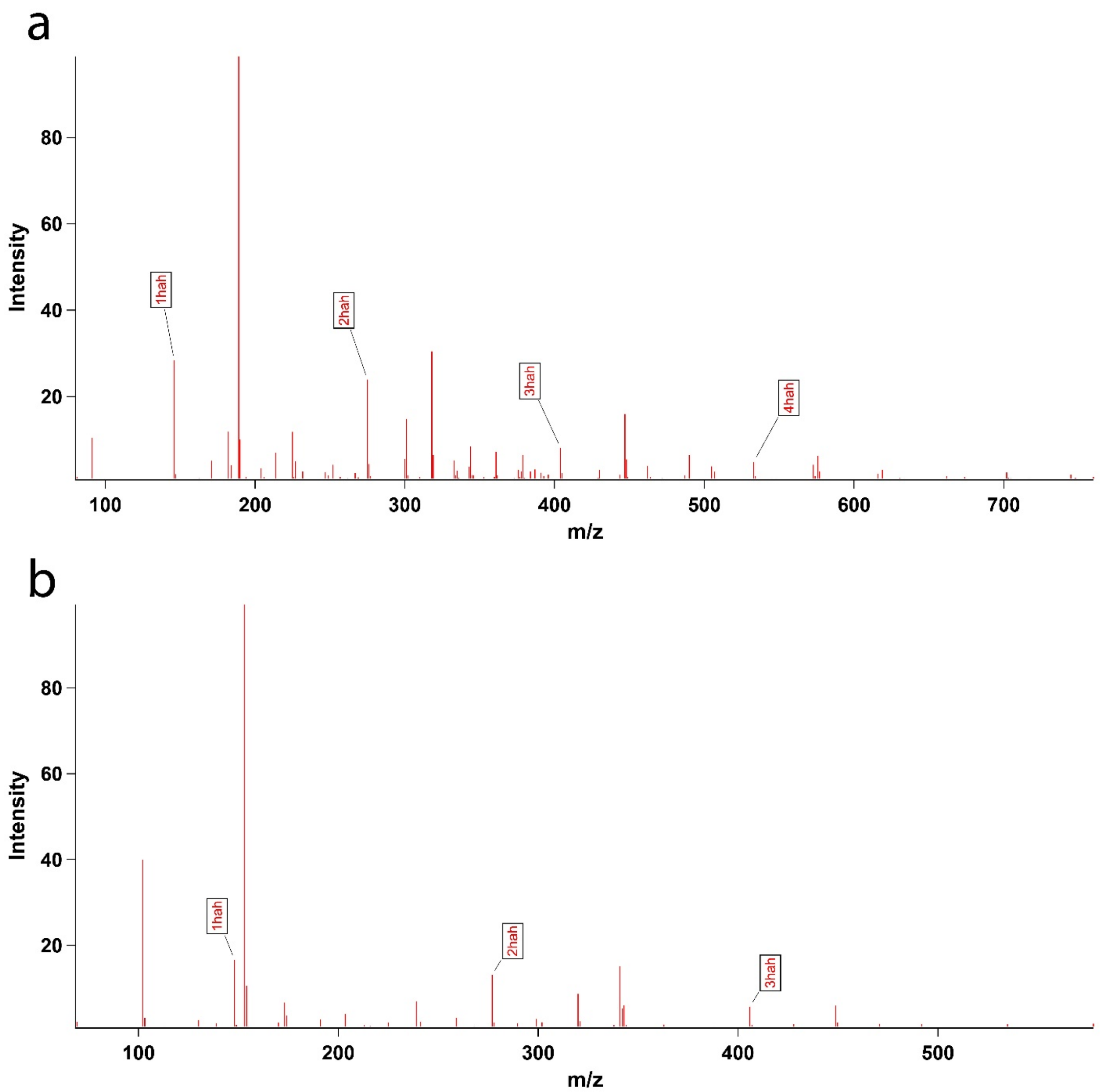

Supplementary Figure 3: ESI-MS of a dry-down reaction of hah supports the formation of polyesters/depsipeptides. **hah** was dried at 85 ˚C for seven days and the resulting polyesters/depsipeptides were analyzed by negative-mode ESI-MS (**a**) or positive mode ESI-MS (**b**), indicating a variety of product. Labeled species correspond to $[M{-}H]^-$ ions (**a**) or $[M{+}H]^+$ ions (**b**).

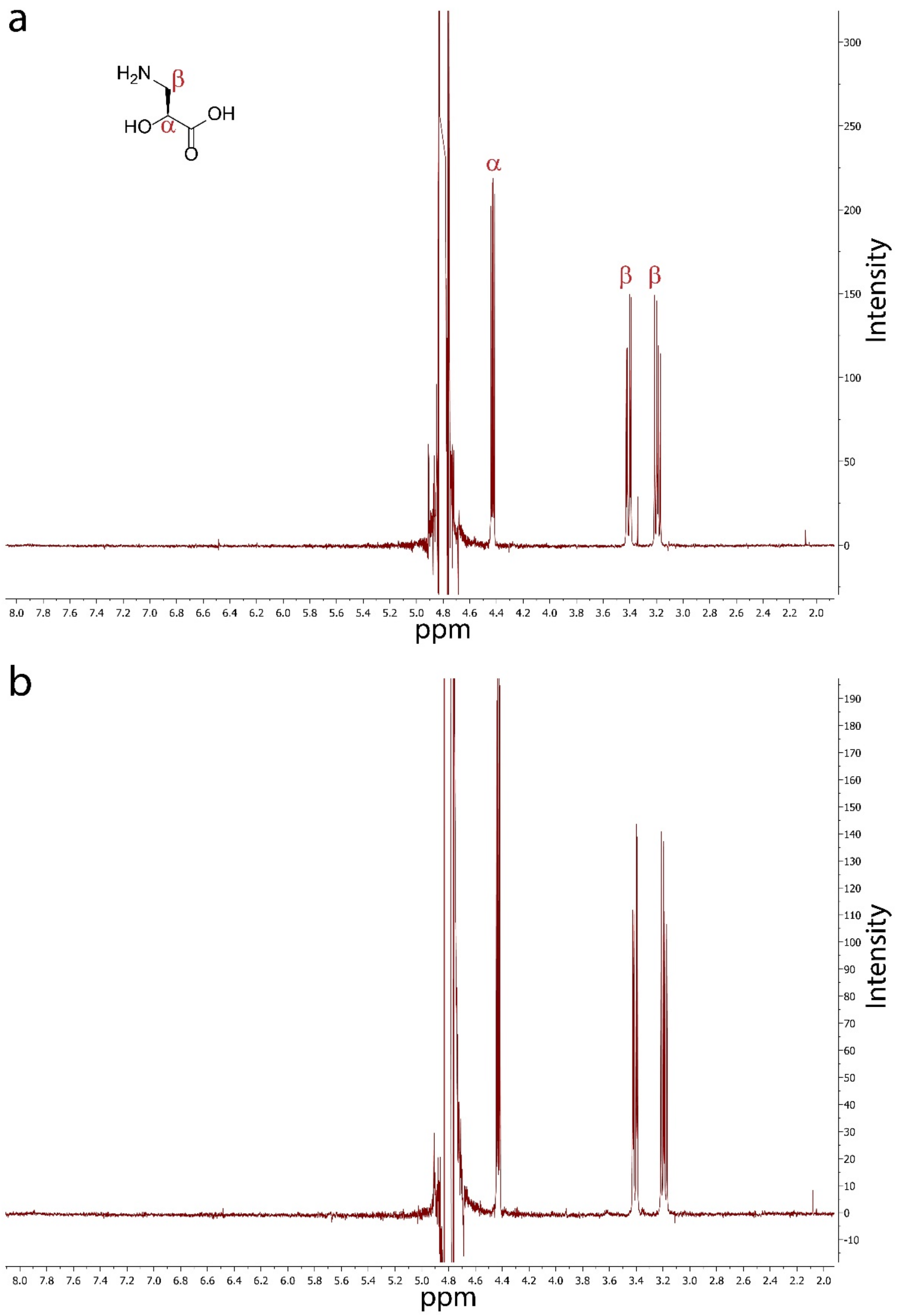

Supplementary Figure 4: ^{1}H NMR Spectrum of isr exhibits low conversion of isr into polymers. ^{1}H NMR spectrum of **isr** in D_2O before (**a**) and after (**b**) dry-down at 85 ˚C for seven days.

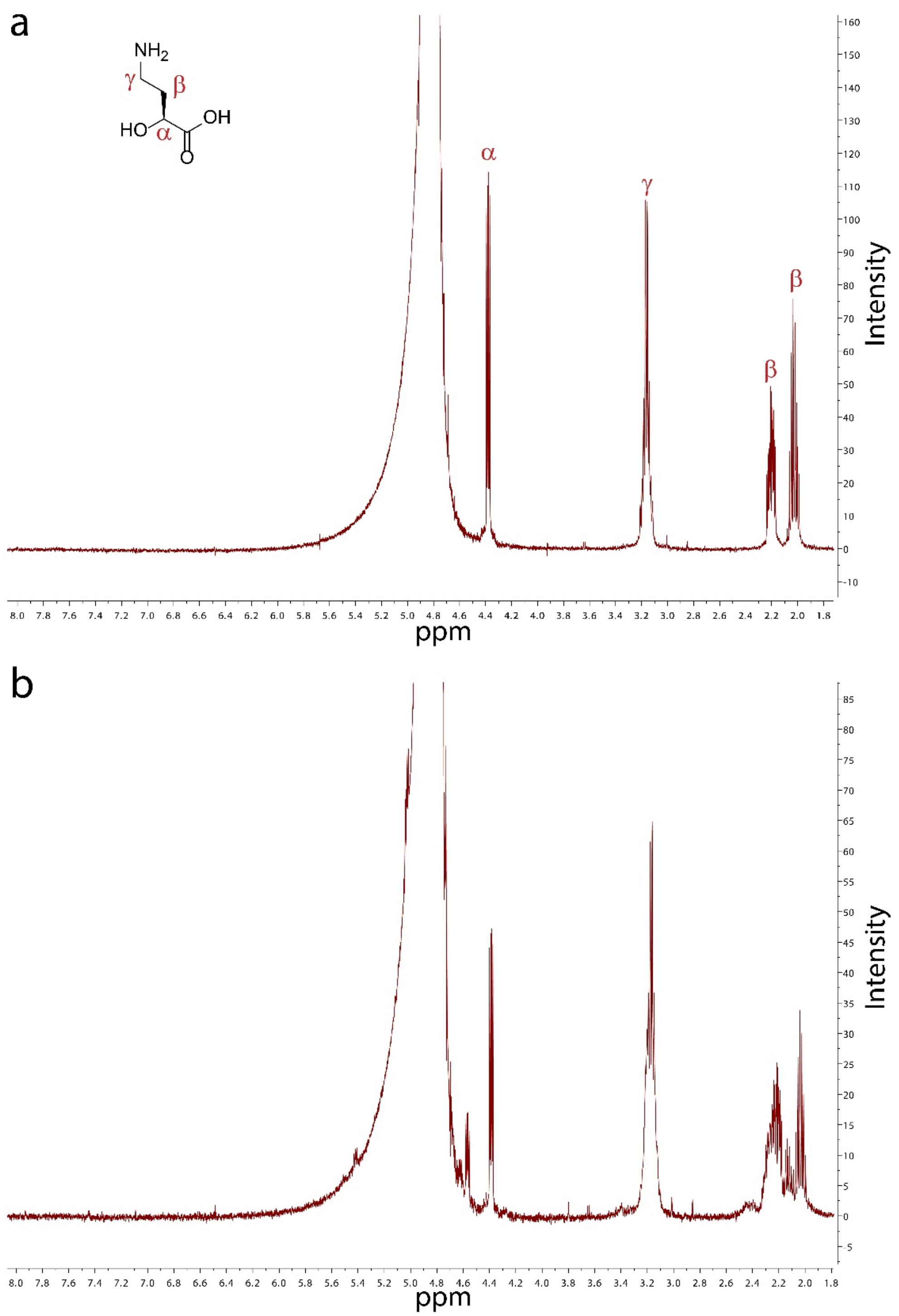

Supplementary Figure 5: ^{1}H NMR Spectrum supports the formation of products upon dry-down reactions of hab. ^{1}H NMR spectrum of **hab** in D_2O before (**a**) and after (**b**) dry-down at 85 ˚C for seven days. Integration of the free un-reacted α-proton of **hab** in the dried sample indicates that 54% of **hab** reacted to form polymers or lactams.

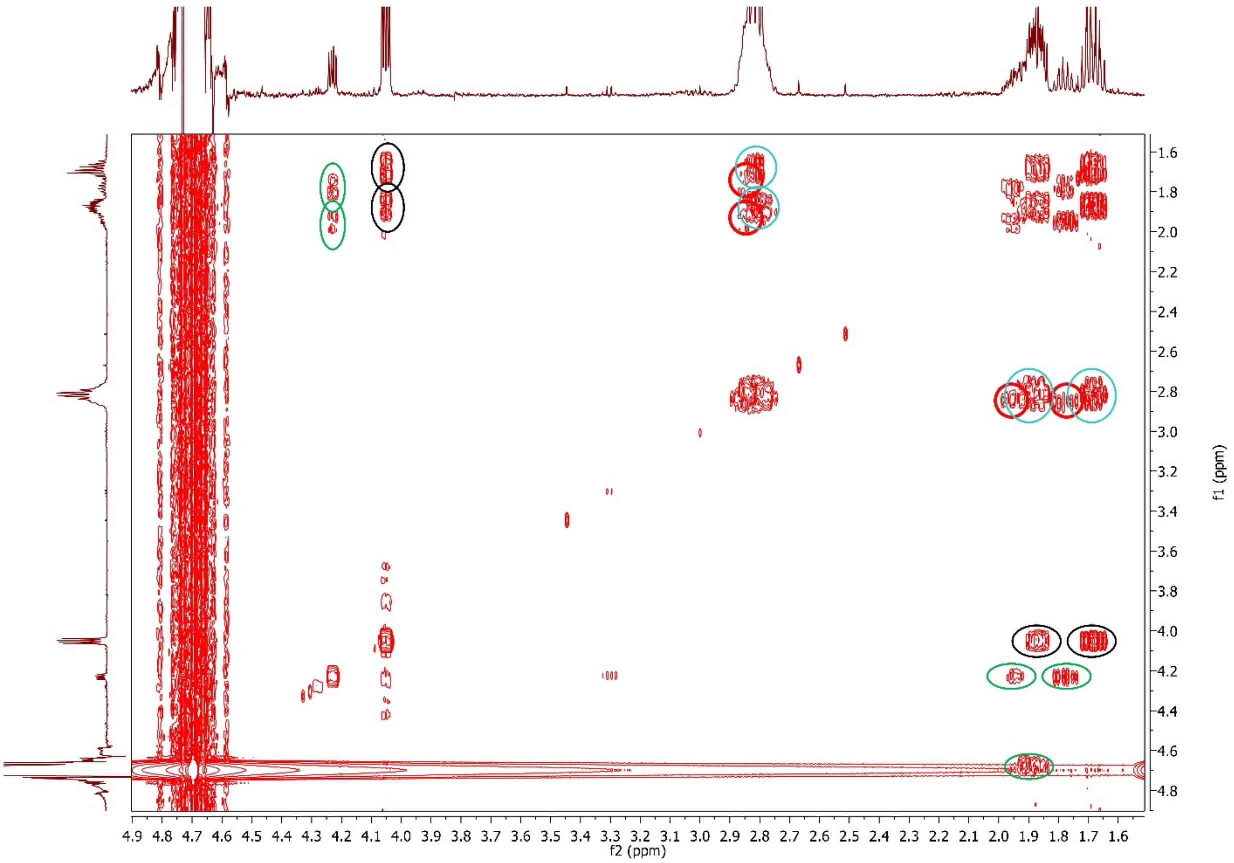

Supplementary Figure 6: ^{1}H-1H COSY NMR spectra of dry-down reactions of hab. **hab** was dried at 85 ˚C for seven days and the resulting product mixture was resuspended in D_2O and analyzed by ^{1}H-^{1}H COSY for peak assignments. Cross peaks of interest are circled: black - correlation between the α-proton and β protons of **hab** that has not been esterified; green - correlations between esterified α-protons and their corresponding β protons; turquoise/red - correlation between γ-protons of **hab** and their corresponding β protons. Based on previous work[6], we speculate that the red correlation indicates γ-protons of γ-amidated **hab** whereas the turquoise correlation indicates γ-protons of **hab** that has free γ-amine (both non-reacted and in polymers).

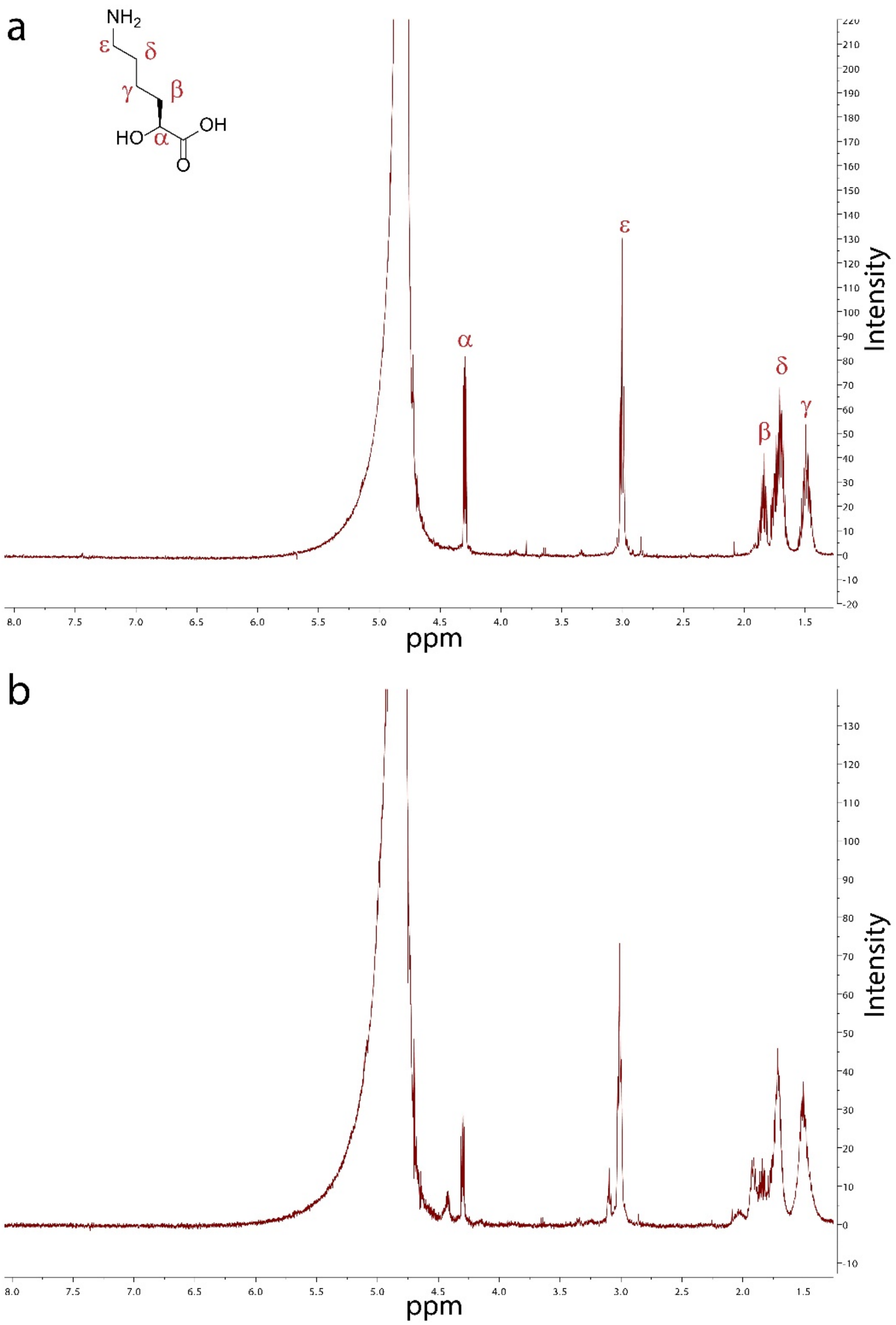

Supplementary Figure 7: ^{1}H NMR Spectrum supports the formation of products upon dry-down reactions of hah. ^{1}H NMR spectrum of **hah** in D_2O before (**a**) and after (**b**) dry-down at 85 ˚C for seven days. Integration of the free un-reacted α-proton of **hah** in the dried sample indicates that 56% of **hah** reacted to form polymers. Moreover, the new down-field ε-protons at ~3.0 ppm are likely a result of ε-amidation (12%), similarly to patterns observed upon ε-amidation of Lys in dry-down reactions[6].

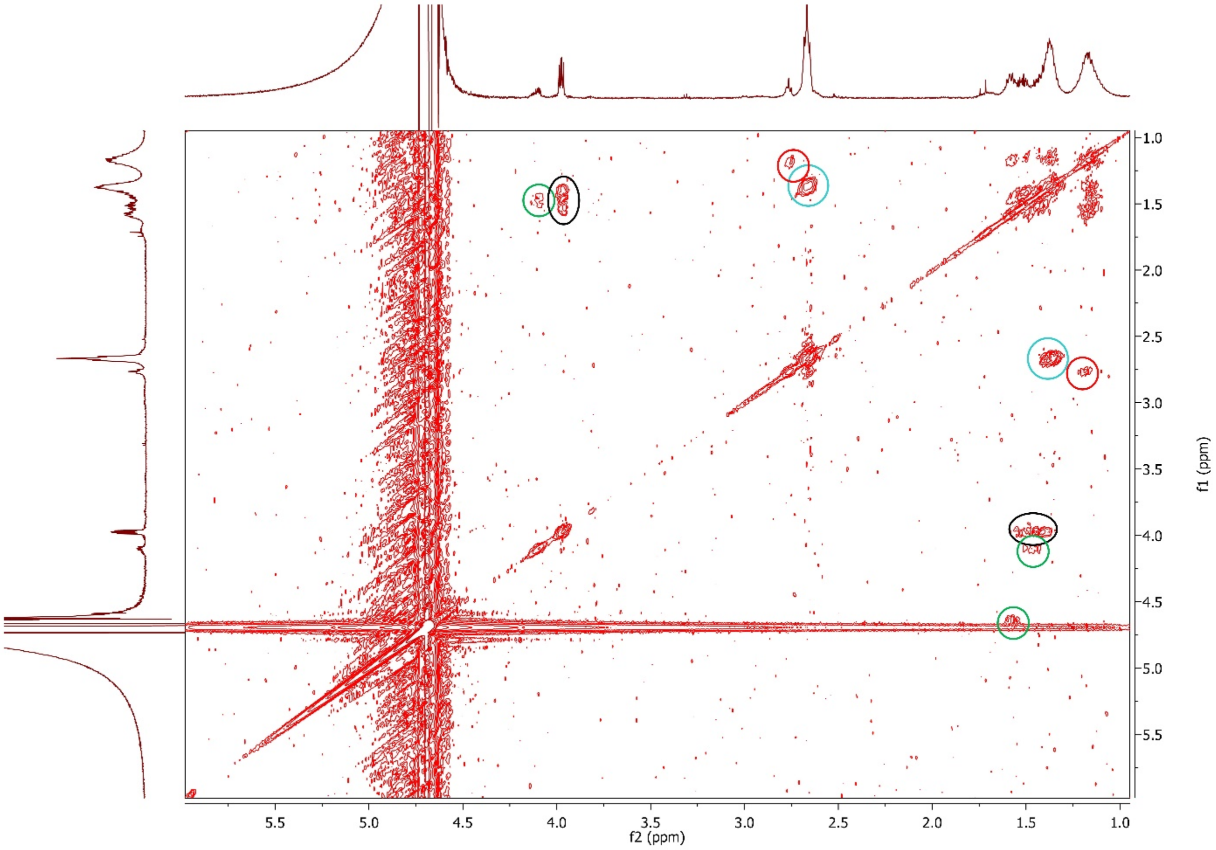

Supplementary Figure 8: ^{1}H-^{1}H COSY NMR spectra of dry-down reactions of hah. hah was dried at 85 ˚C for seven days and the resulting product mixture was resuspended in D_2O and analyzed by ^{1}H-^{1}H COSY for peak assignments. Cross peaks of interest are circled: black - correlation between the α-proton and β protons of **hah** that has not been esterified; green - correlations between esterified α-protons and their corresponding β protons; turquoise/red - correlation between ε-protons of **hah** and their corresponding δ protons. Based on previous work[6], we speculate that the red correlation indicates ε-protons of ε-amidated **hah** whereas the turquoise correlation indicates ε-protons of **hah** that has free ε-amine (both non-reacted and in polymers).

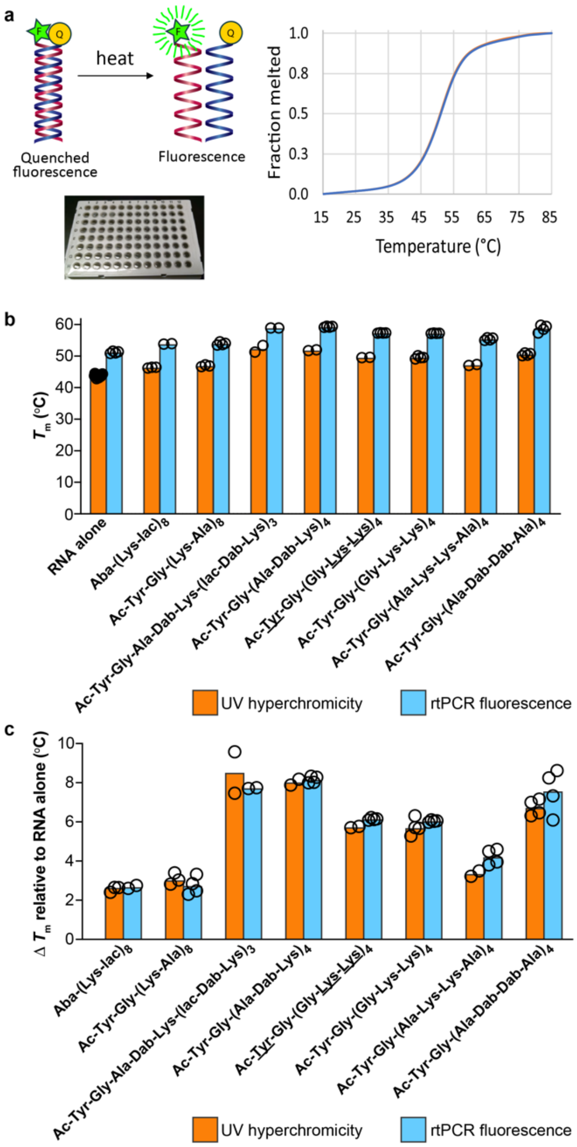

Supplementary Figure 9: Validation of T_m analysis using rtPCR instrumentation. **a**, Overview of the fluorescence-based assay involving rtPCR instrumentation, which was used to determine RNA duplex T_m values in conditions involving dry-down mixtures. **b**, Plot comparing observed RNA duplex T_m as determined by UV hyperchromicity or rtPCR fluorescence. Analyses on the Cary Varian UV spectrophotometer employed an unlabeled RNA duplex, whereas analyses on the BioRad rtPCR instrument employed an RNA duplex in which the strands were labelled with either 5'-FAM or 3'-IABkFQ. **c**, Plot of the data as the change in melting temperature for the various conditions relative to RNA alone. The observed changes in T_m were similar between the two methods of analysis. For panels **b**-**c**, data are shown as a scatter plot (number of independent measurements using UV hyperchromicity: n=16 for RNA alone, n=3 for Aba-(Lys-lac)$_8$, n=3 for Ac-Tyr-Gly-(Lys-Ala)$_8$, n=2 for Ac-Tyr-Gly-Ala-Dab-Lys-(lac-Dab-Lys)$_3$, n=2 for Ac-Tyr-Gly-(Ala-Dab-Lys)$_4$, n=2 for Ac-Tyr-Gly-(Gly-Lys-Lys)$_4$, n=4 for Ac-Tyr-Gly-(Gly-Lys-Lys)$_4$, n=2 for Ac-Tyr-Gly-(Ala-Lys-Lys-Ala)$_4$, n=4 for Ac-Tyr-Gly-(Ala-Dab-Dab-Ala)$_4$; number of independent measurements using fluorescence: n=4 for RNA alone, n=2 for Aba-(Lys-lac)$_8$, n=4 for Ac-Tyr-Gly-(Lys-Ala)$_8$, n=2 for Ac-Tyr-Gly-Ala-Dab-Lys-(lac-Dab-Lys)$_3$, n=4 for Ac-Tyr-Gly-(Ala-Dab-Lys)$_4$, n=4 for Ac-Tyr-Gly-(Gly-Lys-Lys)$_4$, n=4 for Ac-Tyr-Gly-(Gly-Lys-Lys)$_4$, n=4 for Ac-Tyr-Gly-(Ala-Lys-Lys-Ala)$_4$, n=4 for Ac-Tyr-Gly-(Ala-Dab-Dab-Ala)$_4$. Underlined residues denote D-chirality.

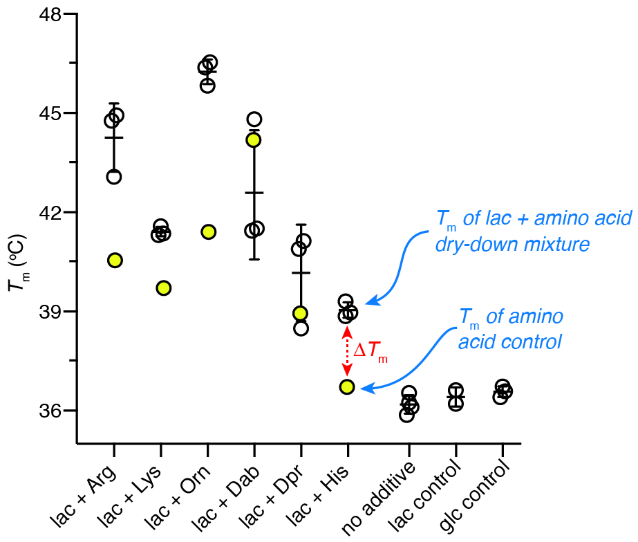

Supplementary Figure 10: Replicate dry-down reactions promote reproducible effects on RNA duplex T_m. Three independent series of replicate dry-down reactions for the lac-containing mixtures were produced. Two series of dry-down reactions were prepared and dried at the same time, while the third replicate was prepared on another day and dried separately. Analogous depsipeptide mixtures from the three sets of reactions (open circles) promoted similar effects on T_m, which were significantly different from amino acid controls (yellow filled circles) for reactions containing **Arg**, **Lys**, **Orn**, and **His**. "Lac control" and "glc control" refer to dry-down reactions containing only lac or glc, respectively. The T_m measurements shown in this figure was carried out in MES-TEA buffer (100 mM) containing 2.5 mM NaCl. The final measured pH of the samples ranged from 5.8-7.1. Data are shown as scatter plot with mean ± SD of the three independent experiments.

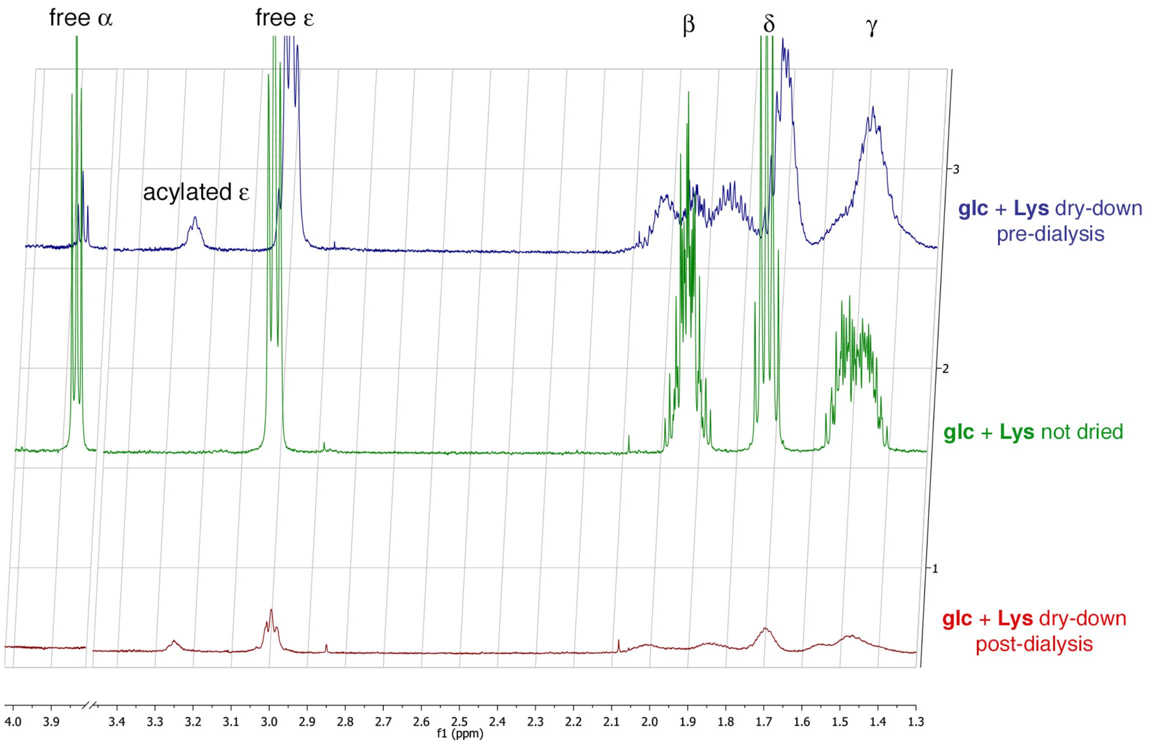

Supplementary Figure 11: Dialysis of dry-down depsipeptide mixtures removes free amino acid. The dry-down mixture containing **glc** and **Lys** was placed in a 500-1000 Da cut-off membrane and dialyzed against water. NMR was used to quantify the concentration of monomeric amino acid and amino acid contained within oligomers following dialysis. ^{1}H NMR spectra of **glc**+**Lys** mixtures are shown before dry-down (*green*), after dry-down at 85 ˚C for seven days but prior to dialysis (*purple*), and post-dialysis (*red*) in D_2O. No trace amount of **Lys** monomer (free α) was observed following dialysis, supporting that the resulting mixtures were largely free of monomeric amino acids and enriched in longer oligomers.

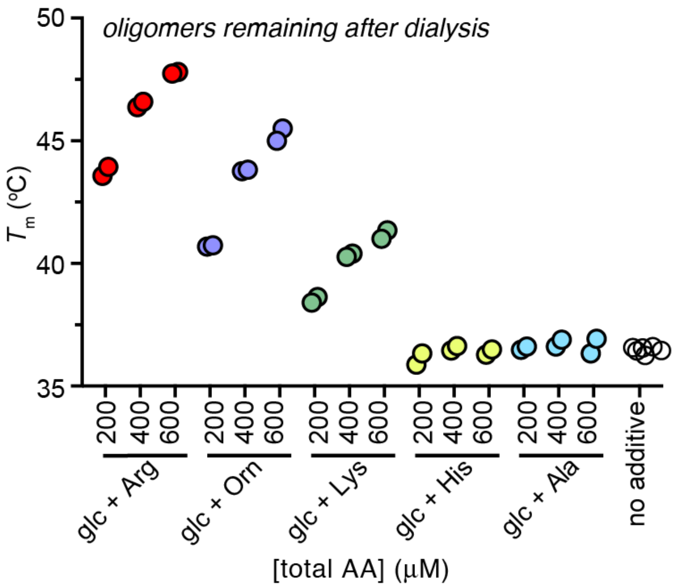

Supplementary Figure 12: Observed T_m values for RNA duplex 1 in the presence of glc-containing depsipeptide oligomers remaining after dialysis. Data are shown as a scatter plot from two independent experiments. The **glc** + **Ala** oligomers are not cationic and were included as a control. For the RNA alone condition, three technical replicates from each of the duplicate experiments are shown, for a total of six data points.

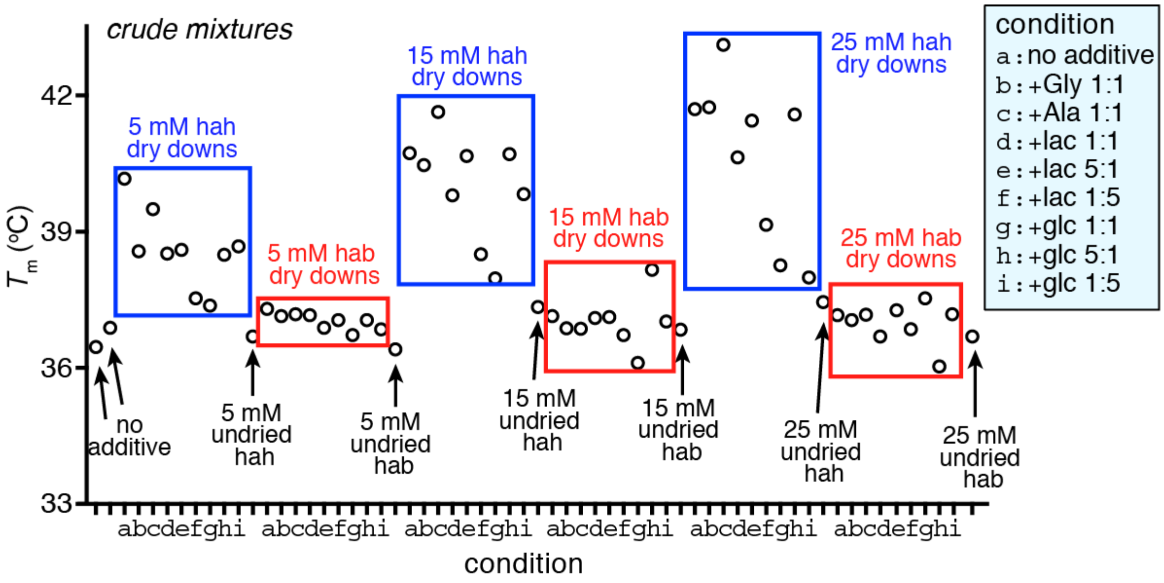

Supplementary Figure 13: Effect of dry-down mixtures of cationic hydroxy acids on the T_m of an RNA duplex. RNA duplex T_m values observed in the presence of undried controls or various crude polyester mixtures obtained by drying **hab** or **hah** with or without amino- or hydroxy-acid additives. The experiment was repeated two times independently with similar results.

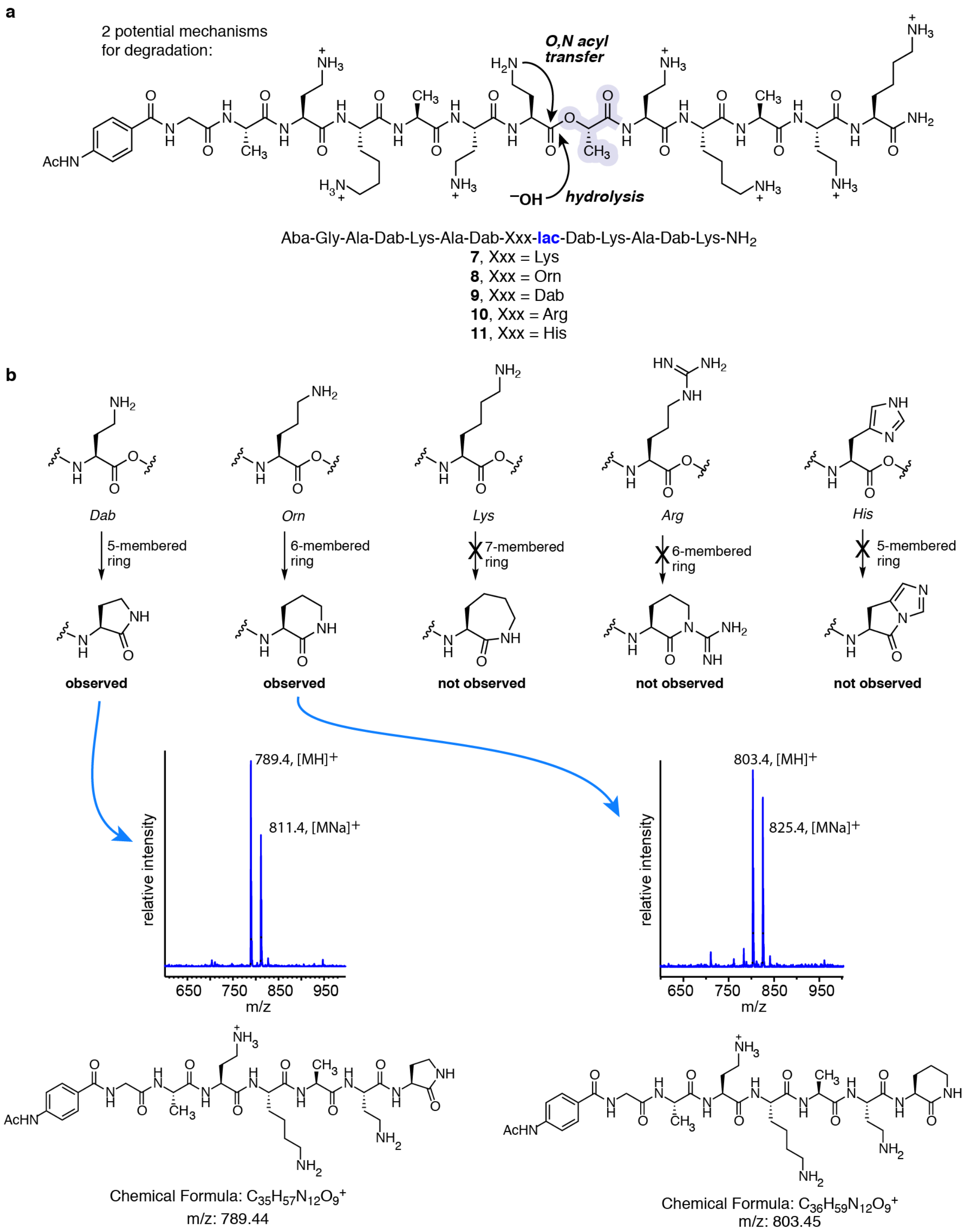

Supplementary Figure 14: Depsipeptides containing Orn and Dab adjacent to ester bonds degrade via O,N-acyl transfer. **a**, Two alternative pathways for depsipeptide degradation, involving direct hydrolysis or O,N acyl transfer. **b**, LCMS results indicated that **Orn**- and **Dab**-containing sequences underwent facile intramolecular O,N-acyl shift transformations, whereas **Lys-**, **Arg-**, and **His**-containing ones did not. All of the sequences were observed to undergo direct hydrolysis. The net result is that **Orn**- and **Dab**-containing depsipeptides degraded faster than the others, reducing their effects on RNA duplex thermal stabilization compared to analogous depsipeptides containing proteinogenic cationic residues.

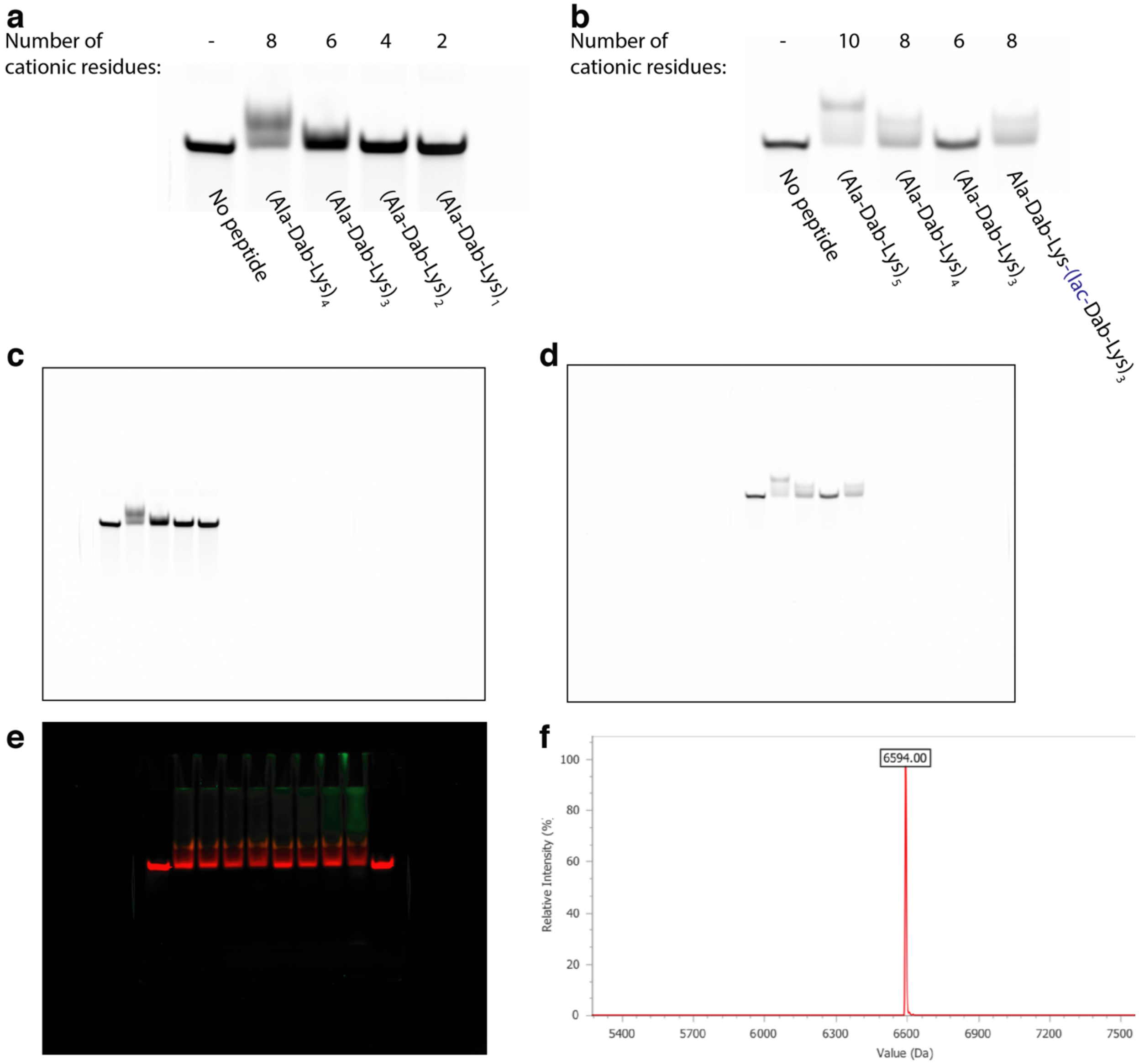

Supplementary Figure 15: Cationic depsipeptides and peptides associate with RNA. **a**, Gel mobility shift assay with cationic peptides (5.3 mM) and 5'-Cy5-U20 (213 µM) in MES-TEA buffer (66.6 mM) shows length-dependent physical association between the interacting molecules, evident as an upward smear of the RNA. The image is representative of two independent experiments giving similar results. **b**, Gel mobility shift assay with cationic peptides or depsipeptide (500 µM) with 5'-Cy5-U20 (26.6 µM) in MES-TEA buffer (66.6 mM) shows length-dependent physical association between the interacting molecules. The image is representative of two independent experiments giving similar results. **c**, Uncropped gel corresponding to image shown in panel '**a**'. **d**, Uncropped gel corresponding to image shown in panel '**b**'. **e**, Uncropped gel corresponding to image shown in Figure 4b. **f**, Quality control mass spectra for 5'-Cy5-U20 oligo used in the gel shift assays, provided by the supplier (IDT).

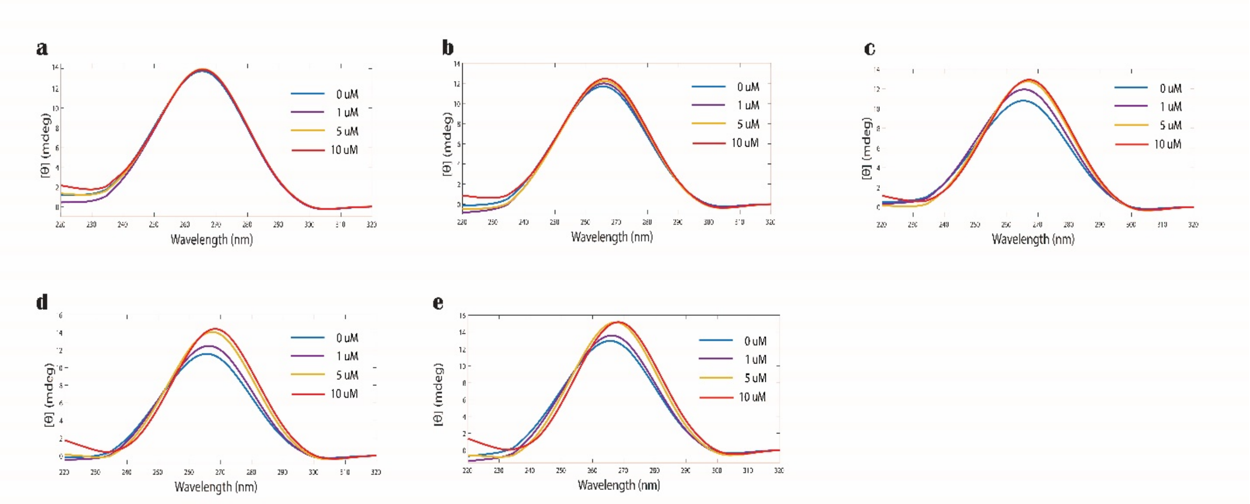

Supplementary Figure 16: CD spectra changes upon addition of cationic peptides. CD spectra of an RNA duplex (5’-rCrGrArUrUrUrArGrCrG-3’ and 3’-rGrCrUrArArArUrCrGrC-5’) at a constant concentration (5 μM) following incubation with varying concentrations (0 – 10 μM) of the following cationic peptides: **a**, Ac-Tyr-Gly-Ala-Dab-Lys-NH_2, **b**, Ac-Tyr-Gly-(Ala-Dab-Lys)$_2$-NH_2, **c**, Ac-Tyr-Gly-(Ala-Dab-Lys)$_3$-NH_2, **d**, Ac-Tyr-Gly-(Ala-Dab-Lys)$_4$-NH_2 and **e**, Ac-Tyr-Gly-(Ala-Dab-Lys)$_5$-NH_2 in MES-TEA buffer (60 mM) shows physical association between various cationic peptides and RNA in a dose-dependent manner.

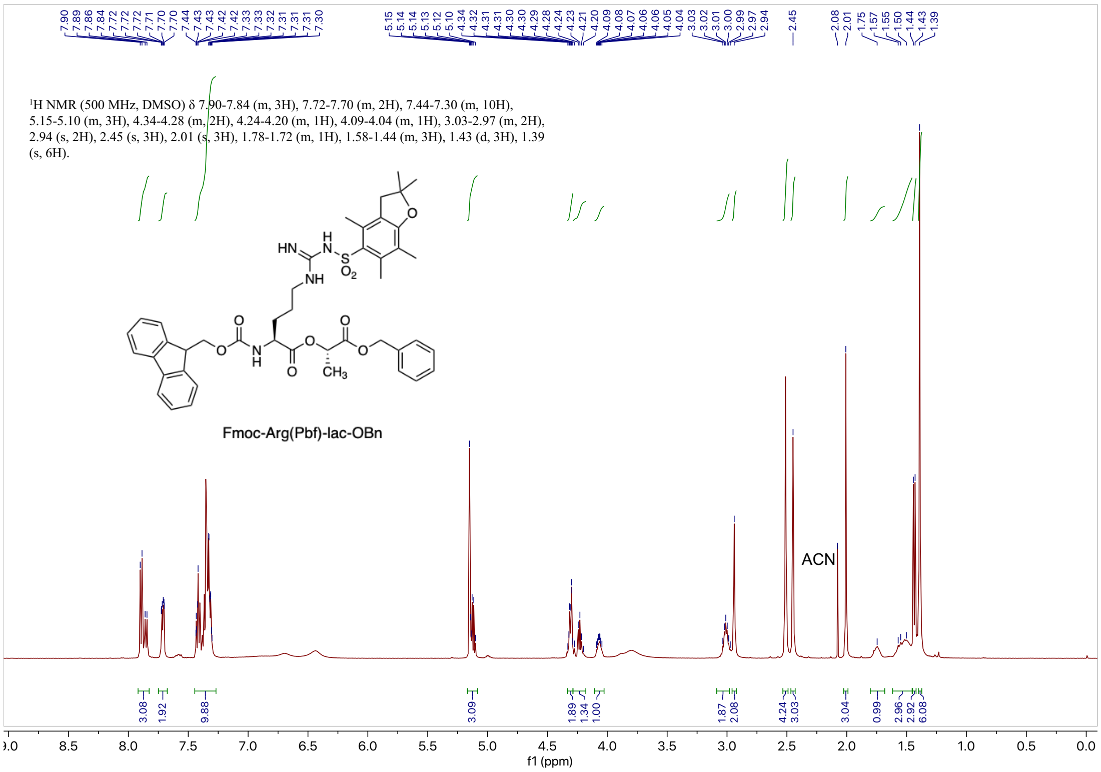

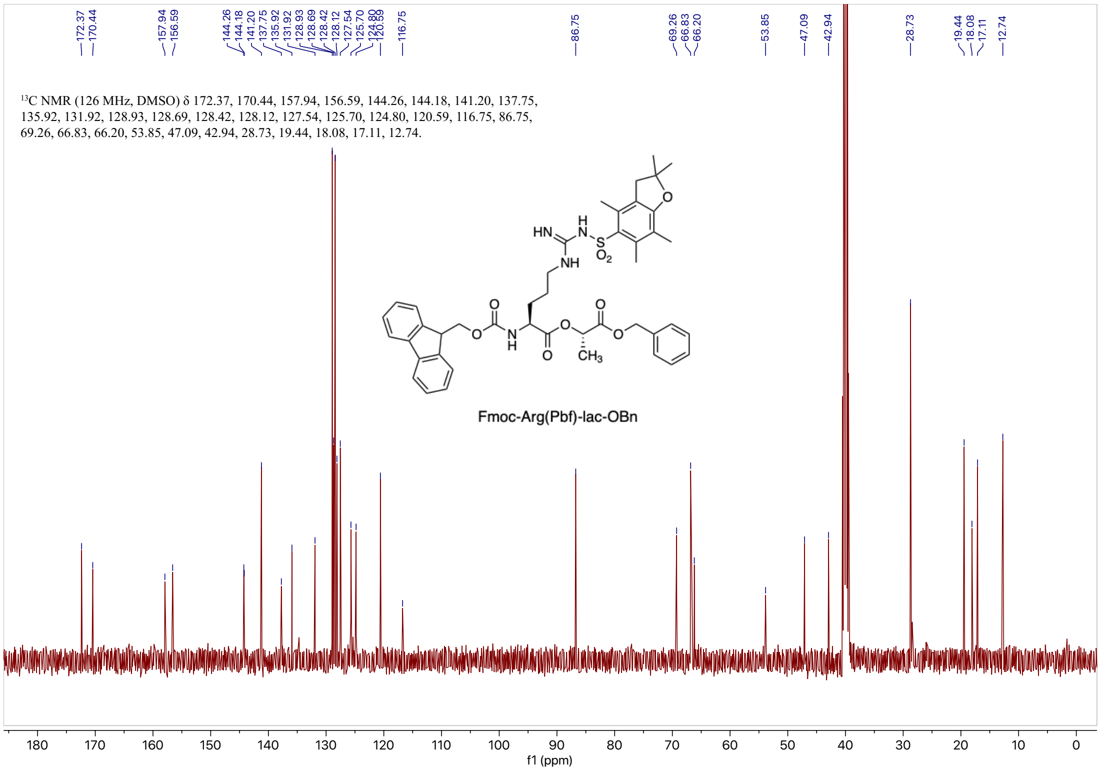

Supplementary Figure 17: ^{1}H- and ^{13}C-NMR spectra of Fmoc-Arg(Pbf)-lac-OBn.

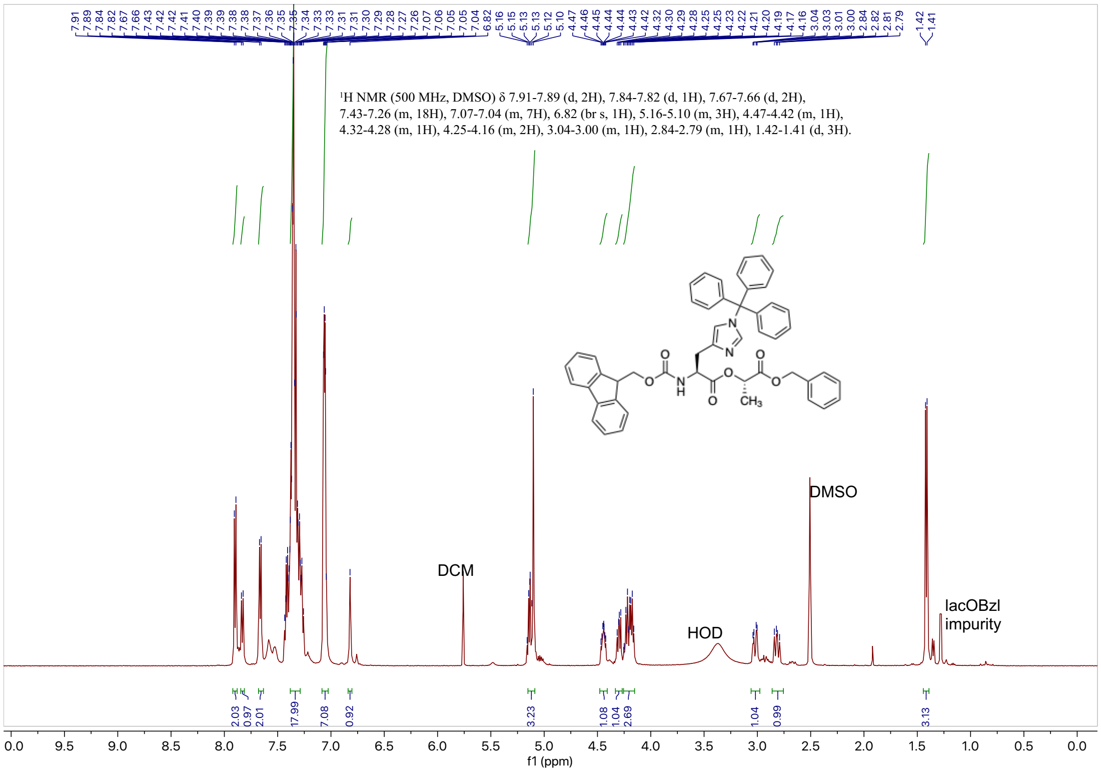

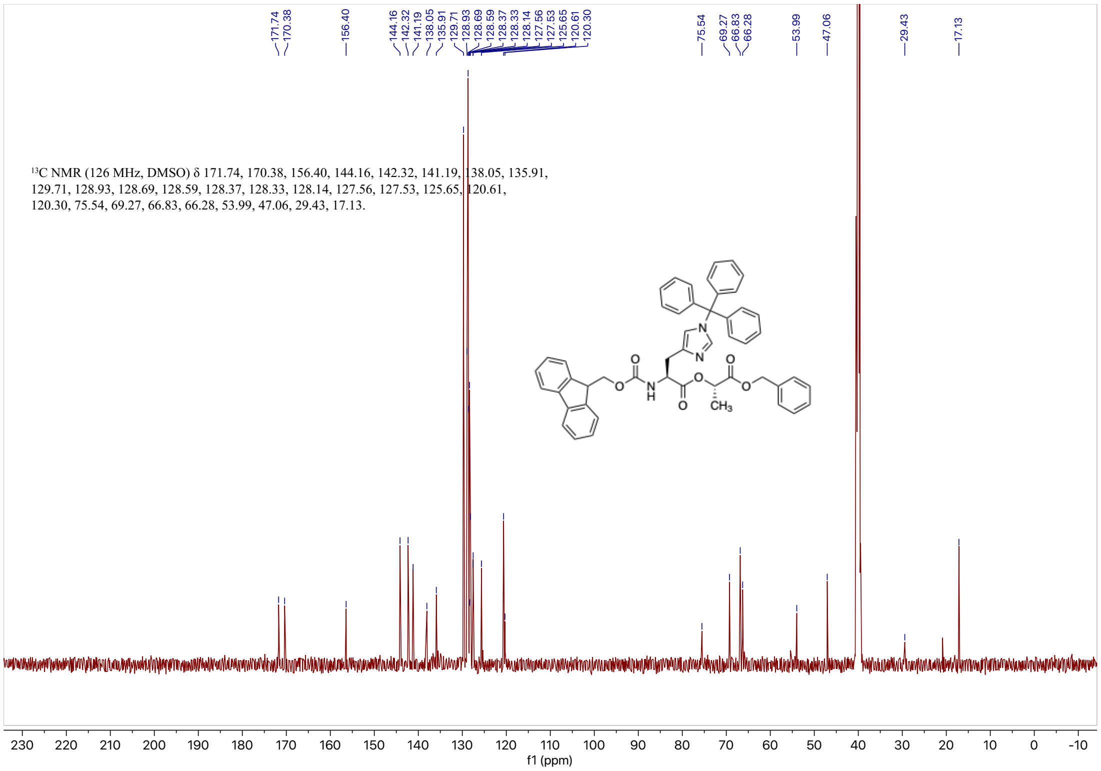

Supplementary Figure 18. ^{1}H- and ^{13}C-NMR spectra of Fmoc-His(Trt)-lac-OBn.

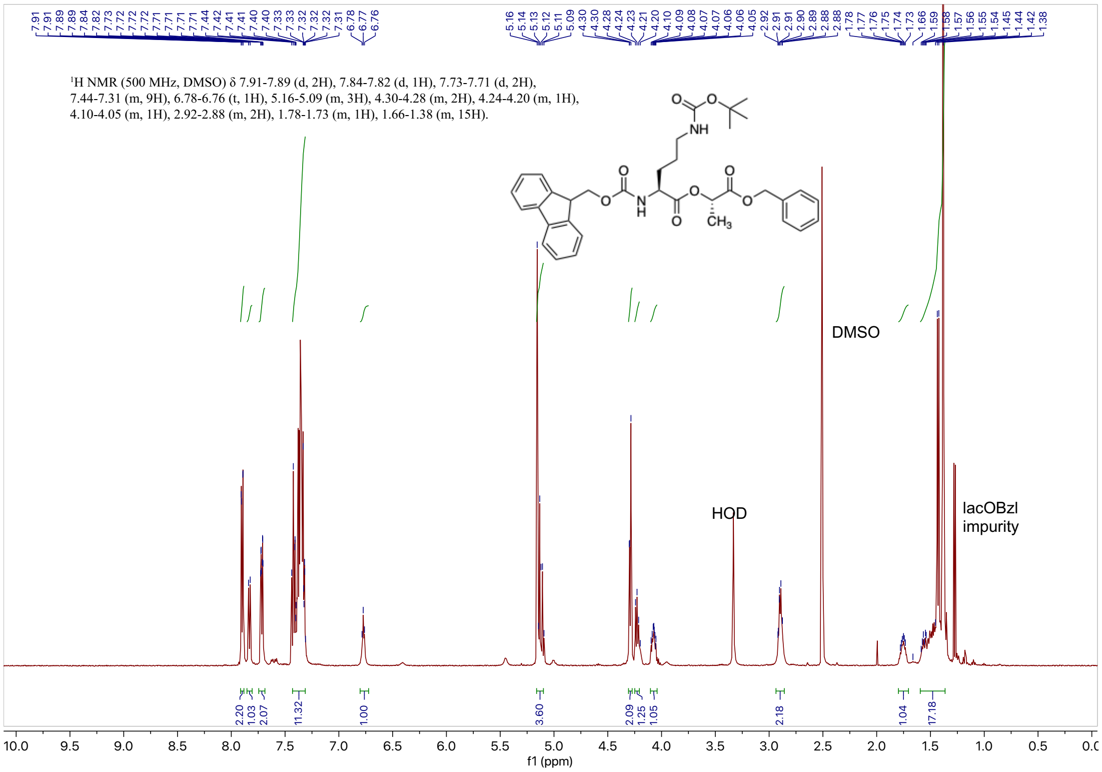

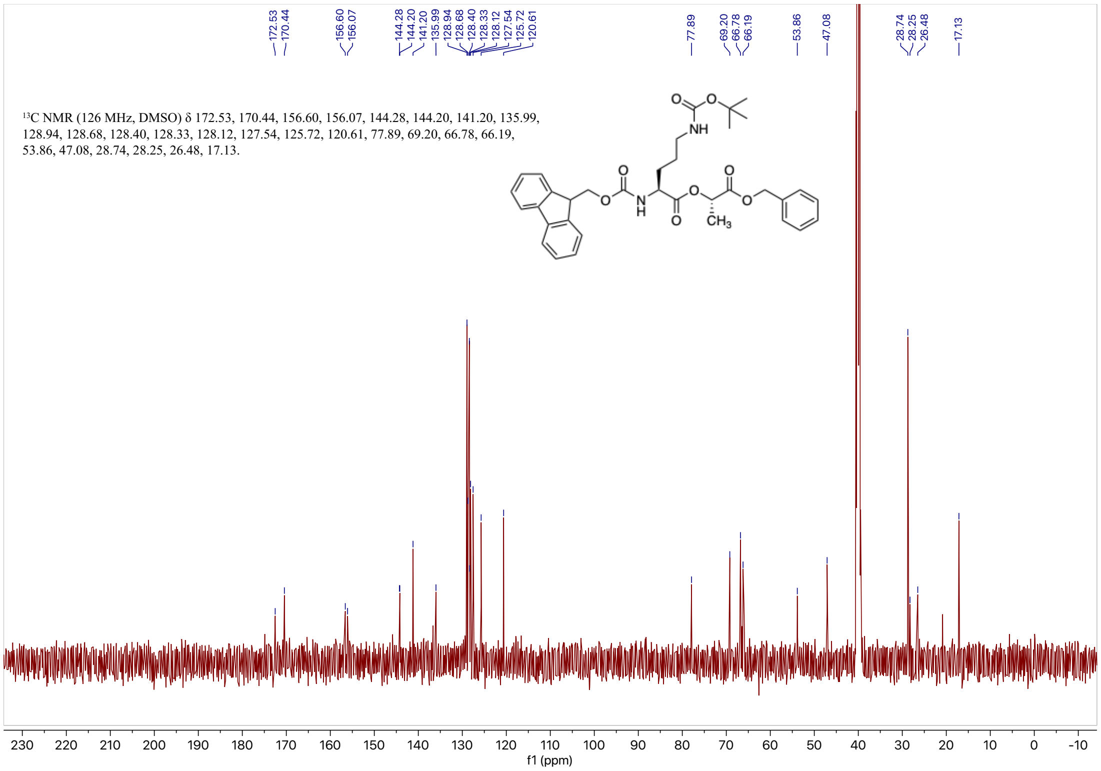

Supplementary Figure 19: ^{1}H- and ^{13}C-NMR spectra of Fmoc-Orn(Boc)-lac-OBn.

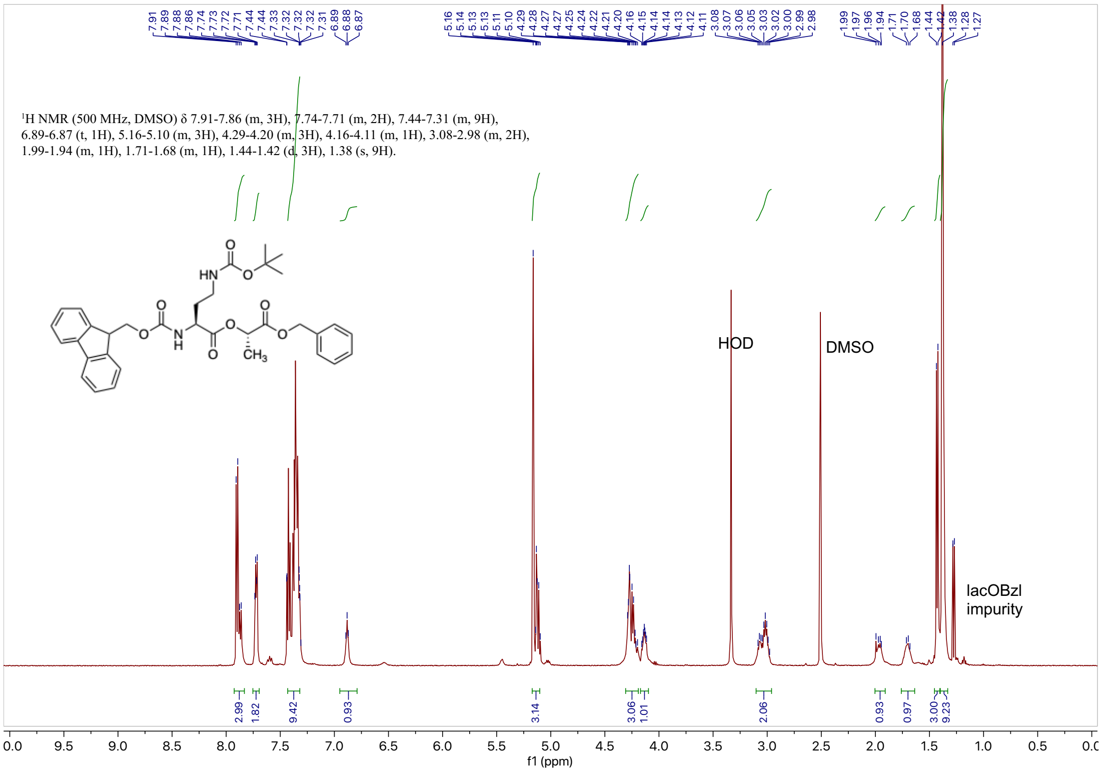

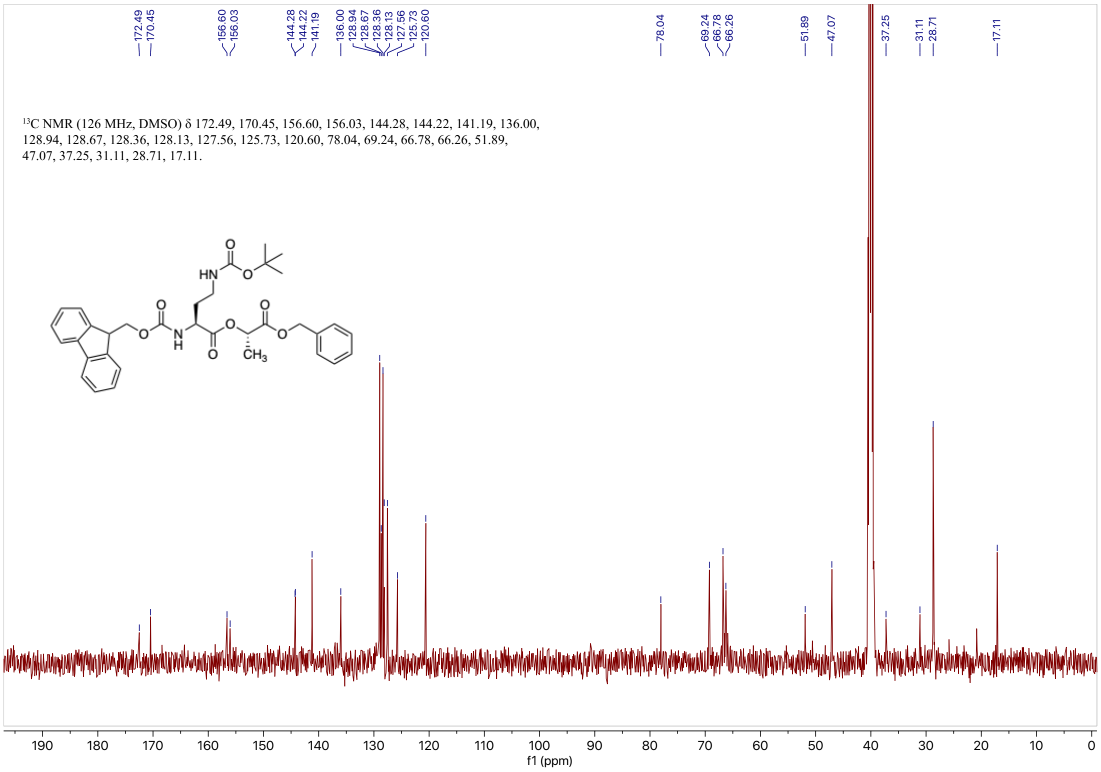

Supplementary Figure 20: ^{1}H- and ^{13}C-NMR spectra of Fmoc-Dab(Boc)-lac-OBn.

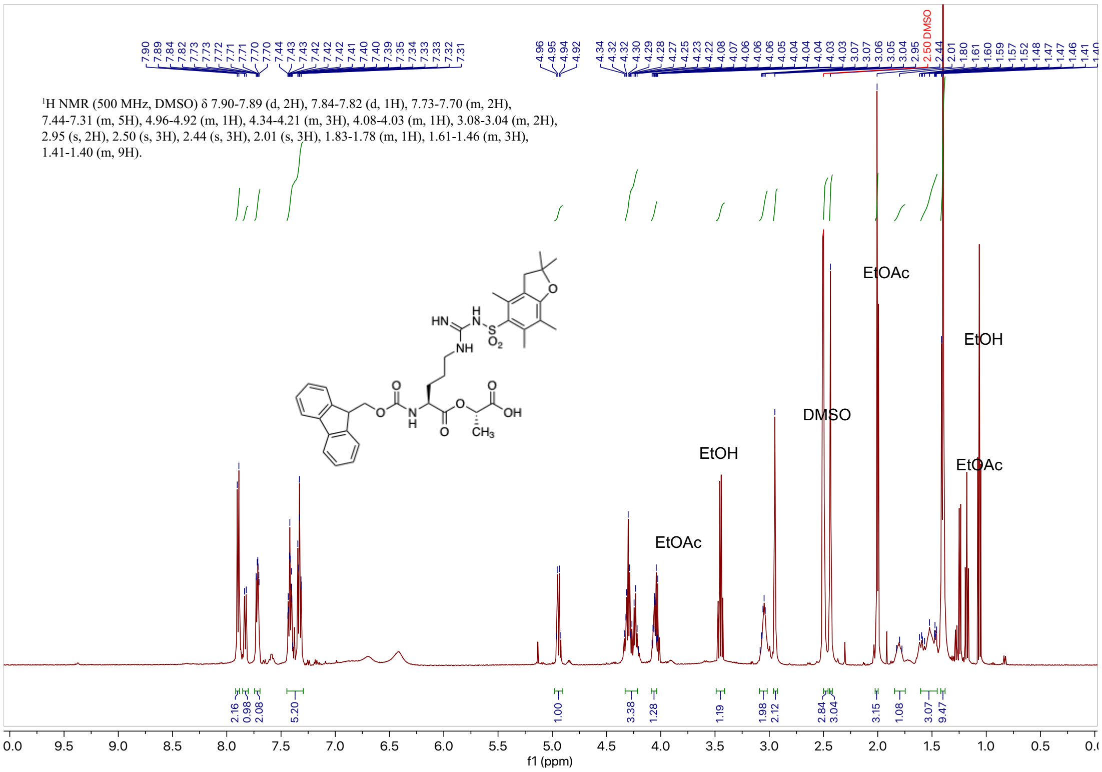

¹H NMR (500 MHz, DMSO) δ 7.90-7.89 (d, 2H), 7.84-7.82 (d, 1H), 7.73-7.70 (m, 2H), 7.44-7.31 (m, 5H), 4.96-4.92 (m, 1H), 4.34-4.21 (m, 3H), 4.08-4.03 (m, 1H), 3.08-3.04 (m, 2H), 2.95 (s, 2H), 2.50 (s, 3H), 2.44 (s, 3H), 2.01 (s, 3H), 1.83-1.78 (m, 1H), 1.61-1.46 (m, 3H), 1.41-1.40 (m, 9H).

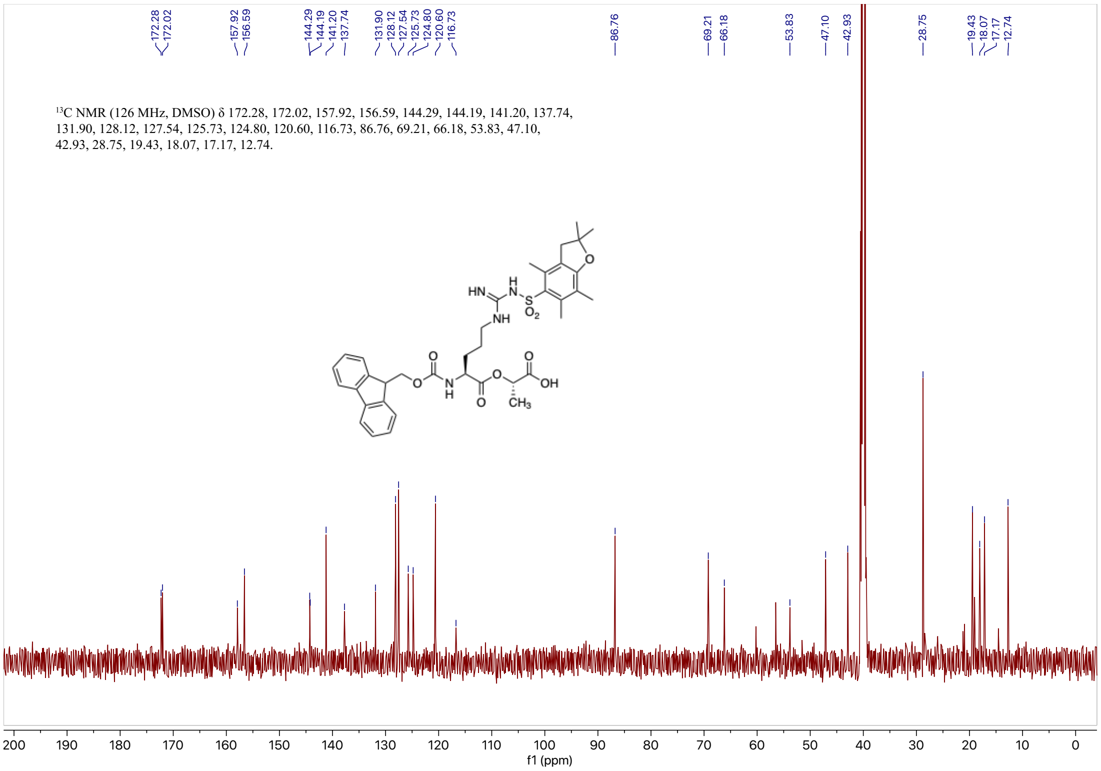

¹³C NMR (126 MHz, DMSO) δ 172.28, 172.02, 157.92, 156.59, 144.29, 144.19, 141.20, 137.74, 131.90, 128.12, 127.54, 125.73, 124.80, 120.60, 116.73, 86.76, 69.21, 66.18, 53.83, 47.10, 42.93, 28.75, 19.43, 18.07, 17.17, 12.74.

Supplementary Figure 21: ¹H- and ¹³C-NMR spectra of Fmoc-Arg(Pbf)-lac-OH.

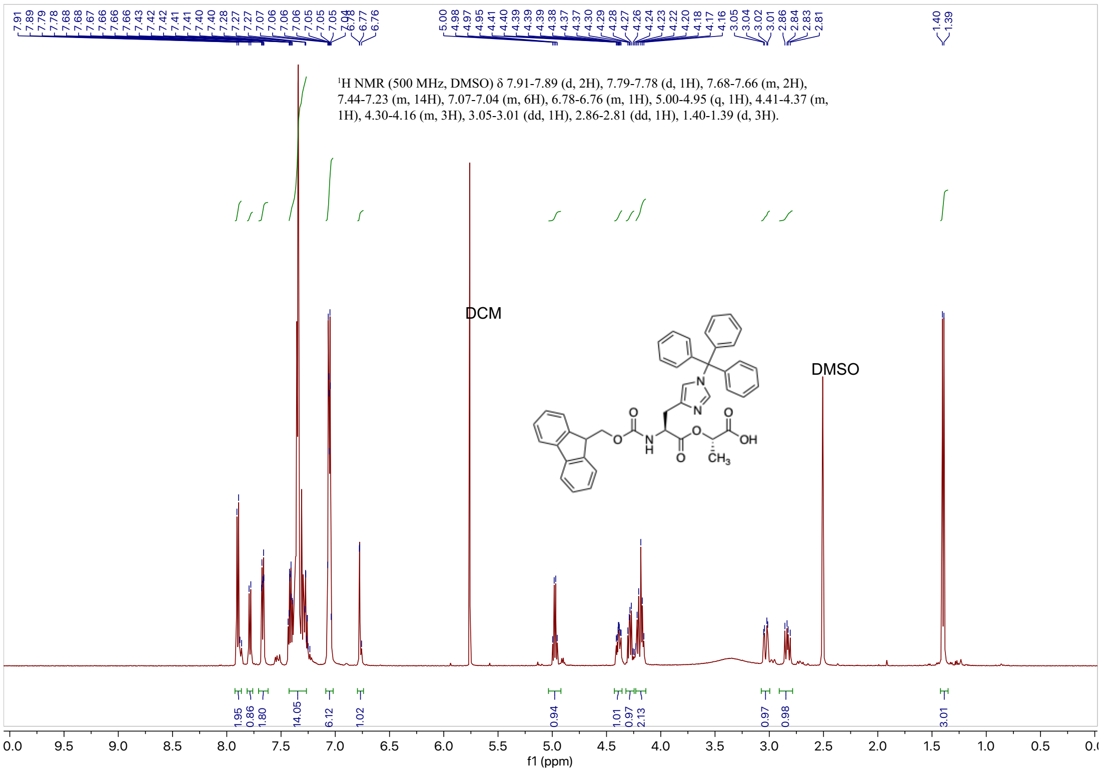

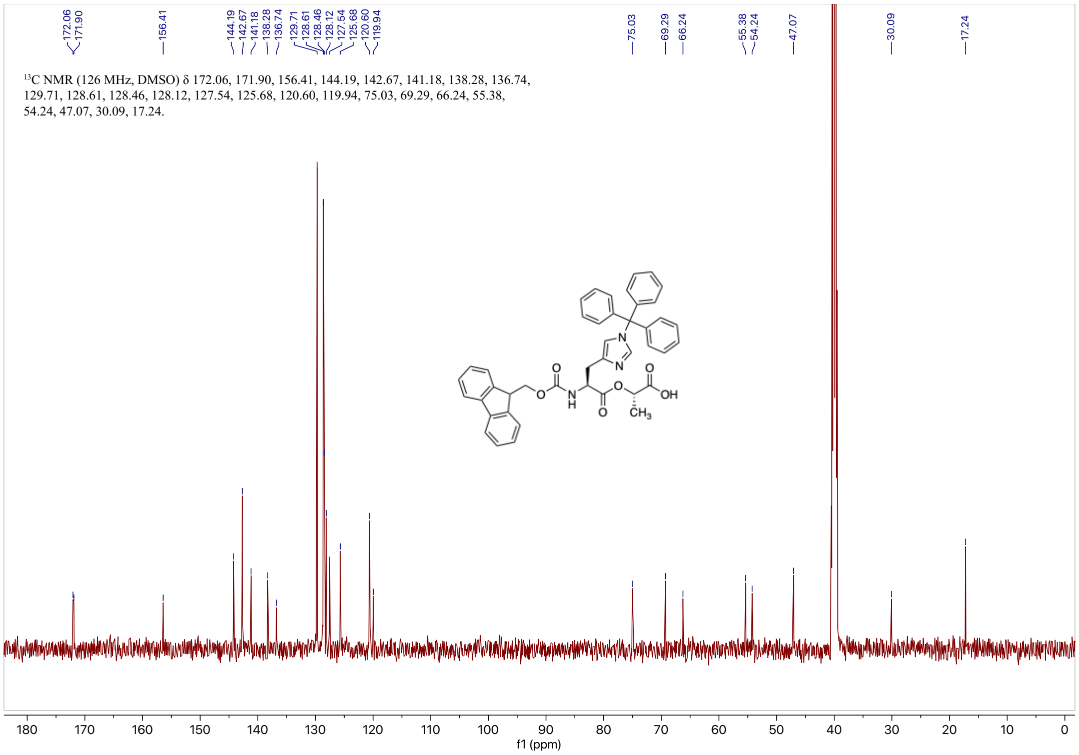

Supplementary Figure 22: ^{1}H- and ^{13}C-NMR spectra of Fmoc-His(Trt)-lac-OH.

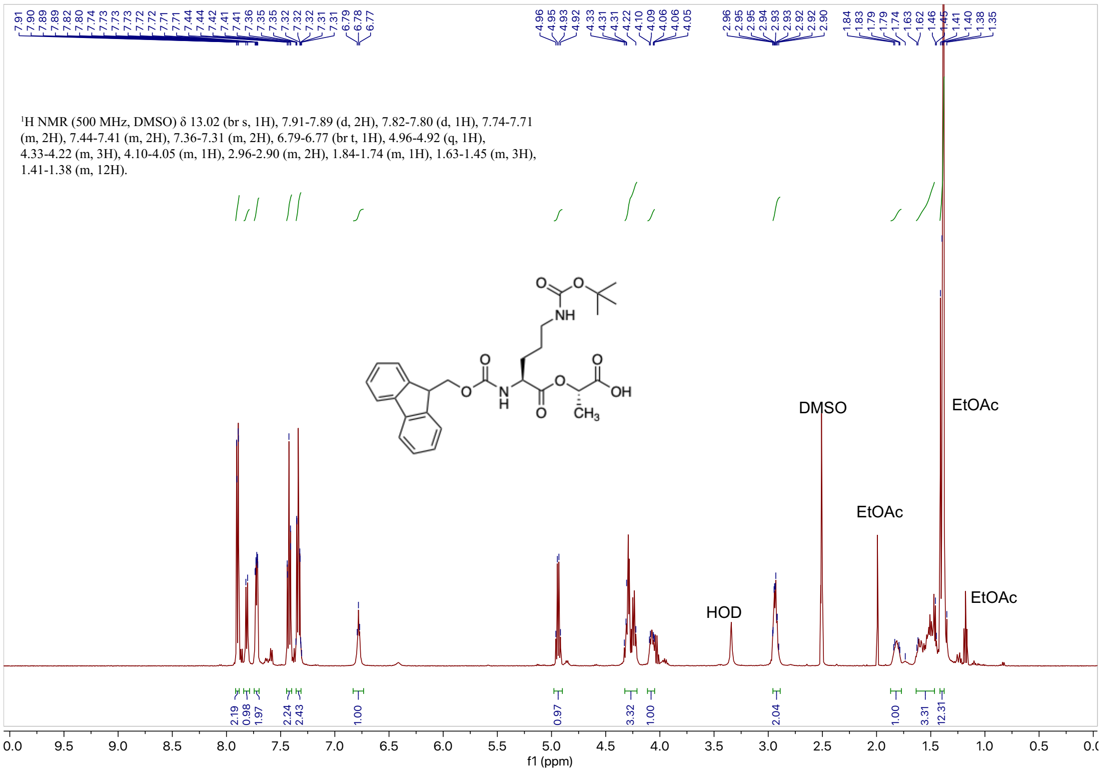

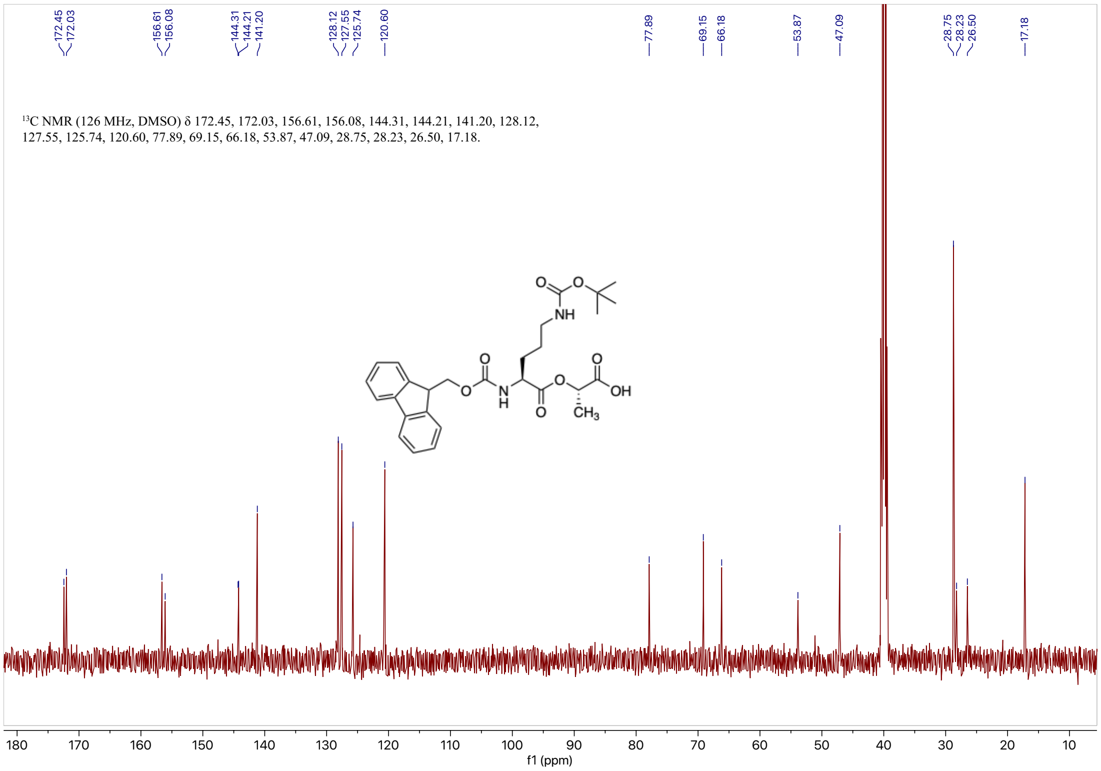

Supplementary Figure 23: ^{1}H- and ^{13}C-NMR spectra of Fmoc-Orn(Boc)-lac-OH.

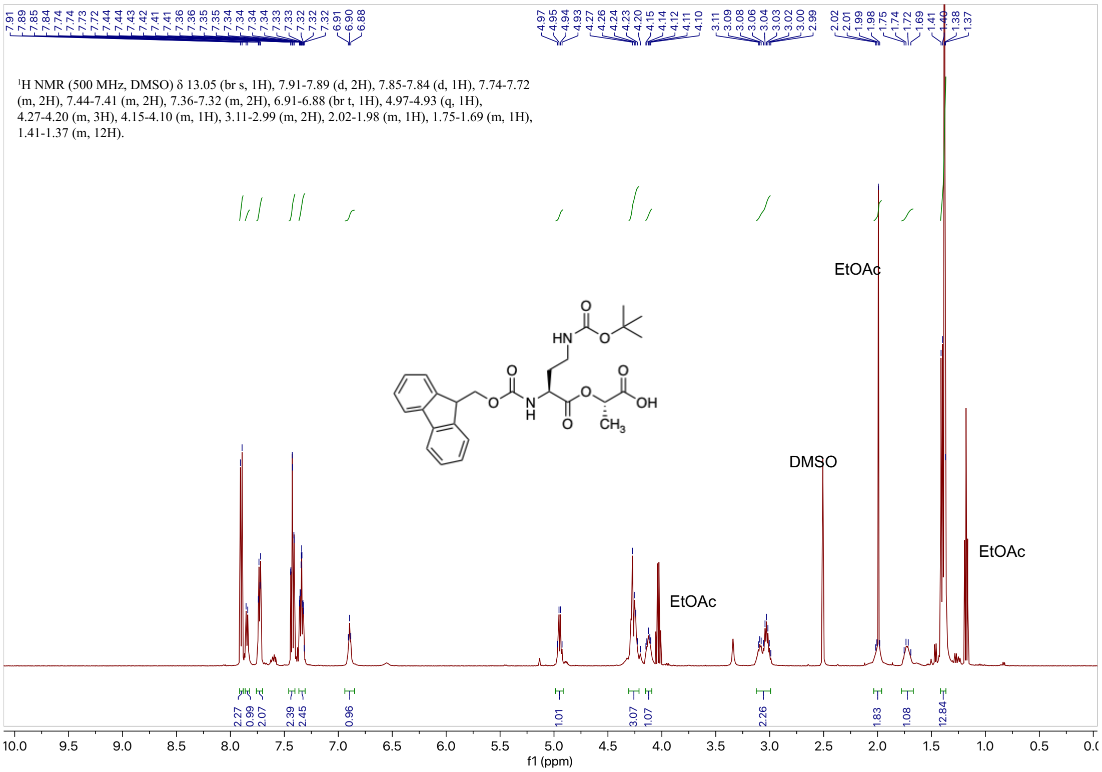

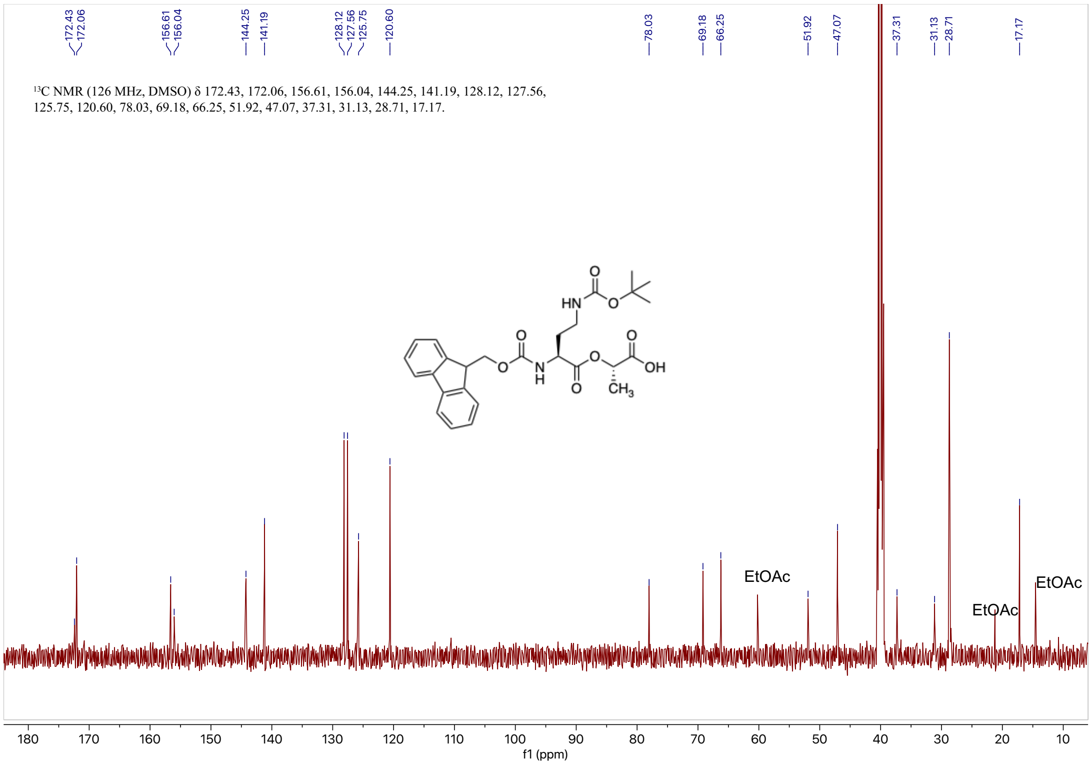

Supplementary Figure 24: ¹H- and ¹³C-NMR spectra of Fmoc-Dab(Boc)-lac-OH.

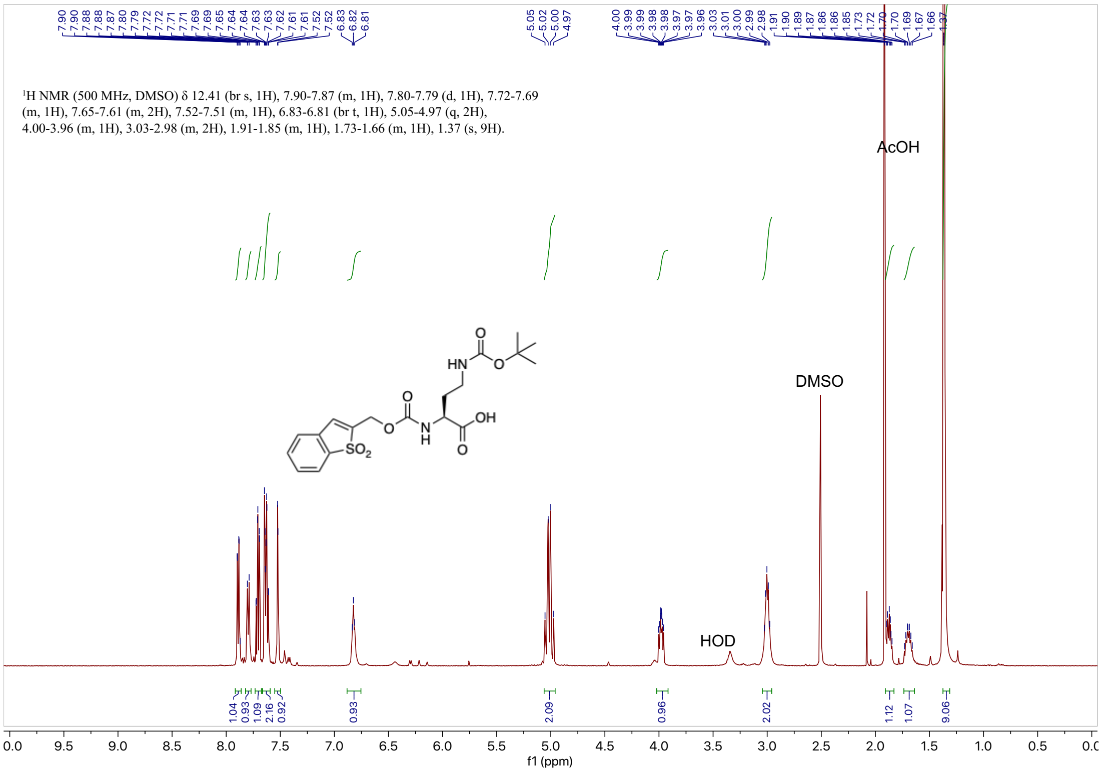

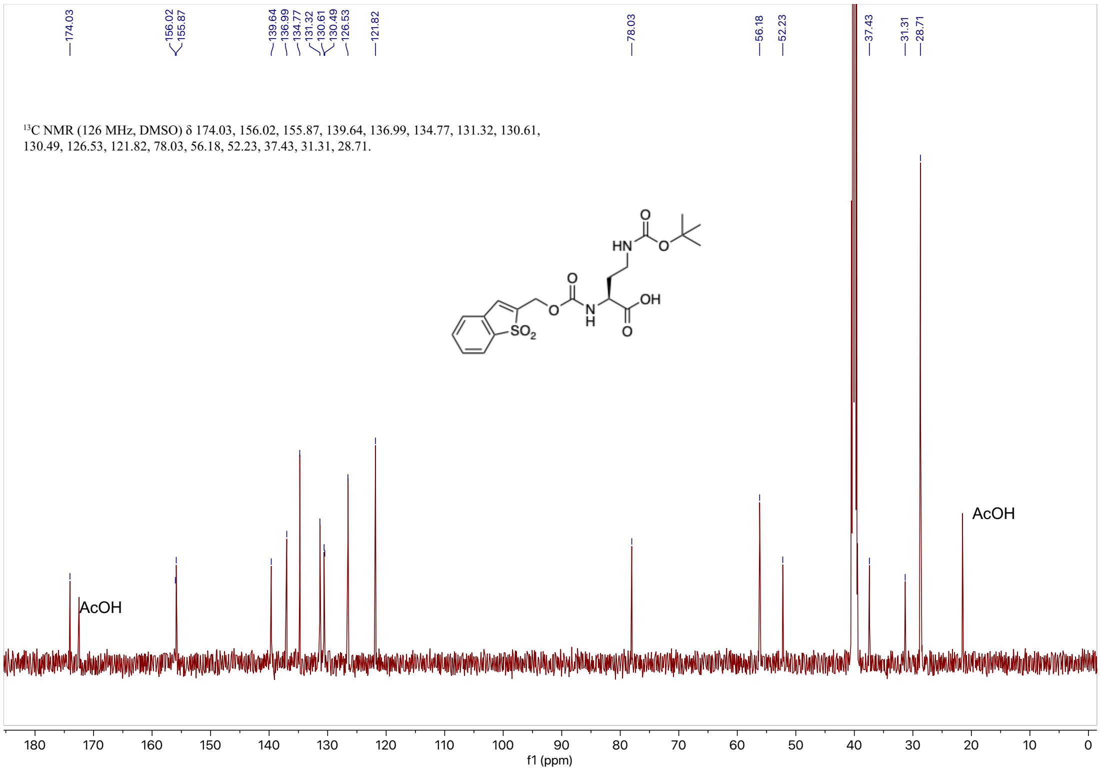

Supplementary Figure 25: ¹H- and ¹³C-NMR spectra of Bsmoc-Dab(Boc)-OH.
